# Applying the CiPA approach to evaluate cardiac proarrhythmia risk of some antimalarials used off‐label in the first wave of COVID‐19

**DOI:** 10.1111/cts.13011

**Published:** 2021-04-09

**Authors:** Annie Delaunois, Matthew Abernathy, Warren D. Anderson, Kylie A. Beattie, Khuram W. Chaudhary, Julie Coulot, Vitalina Gryshkova, Simon Hebeisen, Mark Holbrook, James Kramer, Yuri Kuryshev, Derek Leishman, Isabel Lushbough, Elisa Passini, Will S. Redfern, Blanca Rodriguez, Eric I. Rossman, Cristian Trovato, Caiyun Wu, Jean‐Pierre Valentin

**Affiliations:** ^1^ UCB Biopharma SRL Braine‐l’Alleud Belgium; ^2^ Eli Lilly and Company Lilly Corporate Center Indianapolis Indiana USA; ^3^ Center for Public Health Genomics University of Virginia Charlottesville Virginia USA; ^4^ GlaxoSmithKline Stevenage UK; ^5^ GlaxoSmithKline Collegeville Pennsylvania USA; ^6^ B’SYS GmbH Witterswil Switzerland; ^7^ Certara UK Ltd Sheffield UK; ^8^ Charles River Cleveland Ohio USA; ^9^ Department of Computer Science University of Oxford Oxford UK

## Abstract

We applied a set of in silico and in vitro assays, compliant with the Comprehensive In Vitro Proarrhythmia Assay (CiPA) paradigm, to assess the risk of chloroquine (CLQ) or hydroxychloroquine (OH‐CLQ)‐mediated QT prolongation and Torsades de Pointes (TdP), alone and combined with erythromycin (ERT) and azithromycin (AZI), drugs repurposed during the first wave of coronavirus disease 2019 (COVID‐19). Each drug or drug combination was tested in patch clamp assays on seven cardiac ion channels, in in silico models of human ventricular electrophysiology (Virtual Assay) using control (healthy) or high‐risk cell populations, and in human‐induced pluripotent stem cell (hiPSC)‐derived cardiomyocytes. In each assay, concentration‐response curves encompassing and exceeding therapeutic free plasma levels were generated. Both CLQ and OH‐CLQ showed blocking activity against some potassium, sodium, and calcium currents. CLQ and OH‐CLQ inhibited *I*_Kr_ (half‐maximal inhibitory concentration [IC_50_]: 1 µM and 3–7 µM, respectively) and *I*_K1_ currents (IC_50_: 5 and 44 µM, respectively). When combining OH‐CLQ with AZI, no synergistic effects were observed. The two macrolides had no or very weak effects on the ion currents (IC_50_ > 300–1000 µM). Using Virtual Assay, both antimalarials affected several TdP indicators, CLQ being more potent than OH‐CLQ. Effects were more pronounced in the high‐risk cell population. In hiPSC‐derived cardiomyocytes, all drugs showed early after‐depolarizations, except AZI. Combining CLQ or OH‐CLQ with a macrolide did not aggravate their effects. In conclusion, our integrated nonclinical CiPA dataset confirmed that, at therapeutic plasma concentrations relevant for malaria or off‐label use in COVID‐19, CLQ and OH‐CLQ use is associated with a proarrhythmia risk, which is higher in populations carrying predisposing factors but not worsened with macrolide combination.

**Study Highlights**

**WHAT IS THE CURRENT KNOWLEDGE ON THE TOPIC?**

The antimalarials chloroquine (CLQ) or hydroxychloroquine (OH‐CLQ) used off‐label in combination with a macrolide antibiotic (erythromycin [ERT] or azithromycin [AZI]) during the first wave of coronavirus disease 2019 (COVID‐19) have been long associated with cardiovascular side effects, namely QT prolongation. There is a paucity of nonclinical data, meeting the new Comprehensive In Vitro Proarrhythmia Assay (CiPA) paradigm, to properly assess the proarrhythmic risk of these drugs, used alone as antimalarials or combined with a macrolide, such as in some COVID‐19 clinical trials.

**WHAT QUESTION DID THIS STUDY ADDRESS?**

What is the propensity of CLQ and OH‐CLQ to affect cardiac ion currents and to delay ventricular repolarization using the set of in vitro and in silico assays proposed by the CiPA approach? What is the associated safety margin compared with therapeutic plasma levels? Does the combination with AZI or ERT aggravate this risk?

**WHAT DOES THIS STUDY ADD TO OUR KNOWLEDGE?**

The risk of QT prolongation and Torsades de Pointes (TdP) liability was consistently demonstrated in all CiPA assays for both CLQ and OH‐CLQ at exposure levels relevant for malaria treatment or off‐label use in COVID‐19 and is primarily due to *I*_Kr_ current blockade. This risk is higher in populations carrying predisposing factors. Combination with AZI or ERT does not worsen the CLQ/OH‐CLQ TdP risk in virtual ventricular myocyte models or human‐induced pluripotent stem cell‐derived cardiomyocytes.

**HOW MIGHT THIS CHANGE CLINICAL PHARMACOLOGY OR TRANSLATIONAL SCIENCE?**

Our integrated nonclinical dataset supports the US Food and Drug Administration (FDA)/European Medicines Agency (EMA) recommendations to apply strong caution in CLQ and OH‐CLQ use, in particular with high‐dose regimens and/or in patients with additional risk factors, such as in COVID‐19. The combination of these drugs with a macrolide does not worsen the QT prolongation/TdP risk.

## INTRODUCTION

The concept of the Comprehensive In Vitro Proarrhythmia Assay (CiPA) paradigm was proposed jointly by the US Food and Drug Administration (FDA), the Health and Environmental Sciences Institute (HESI), and the Cardiac Safety Research Consortium (CSRC) in 2013 to improve on prediction of risk of Torsade de Pointes (TdP) for small molecule pharmaceuticals.^1^, ^2^, ^3^, ^4^ It comprises both nonclinical and clinical components. The three nonclinical elements of CiPA are (1) optimization and standardization of automated cardiac ion channel assays, (2) in silico modeling of the effects on action potential shape from the known interactions of the compound across key cardiac ion channels, and (3) effects of the compound on action potential and contractility biomarkers in human‐induced pluripotent stem cell (hiPSC)‐derived cardiomyocytes. Although the CiPA paradigm is still being refined and validated, we took the opportunity to “road‐test” this approach with some repurposed drugs that have recently been under intense clinical, media, and public scrutiny.

Chloroquine (CLQ) and hydroxychloroquine (OH‐CLQ) are long‐established medicines used for prophylaxis and treatment of uncomplicated malaria or some autoimmune diseases. Their use has been associated with cardiovascular side effects, in particular QT interval prolongation and TdP.^5^ CLQ and OH‐CLQ were developed and marketed long before the introduction of the ICH S7B^6^ and E14^7^ guidelines, which provide recommendations for the nonclinical and clinical assessment of the potential of a new drug to delay ventricular repolarization and prolong QT interval. Consequently, the availability of high quality, nonclinical and clinical cardiovascular data, meeting current and emerging scientific best practices and regulatory framework,^8^, ^9^ is relatively limited. A few former publications reported that CLQ inhibits *I*_Kr_ current with a half‐maximal inhibitory concentration (IC_50_) value of 2.5 µM^10^ and prolongs QTc interval in ex vivo and in vivo rabbit models,^11^ as well as in healthy subjects.^12^

When the new coronavirus disease 2019 (COVID‐19) appeared in December 2019, early in vitro studies reported antiviral activity of CLQ and OH‐CLQ against the severe acute respiratory syndrome‐coronavirus 2 (SARS‐CoV‐2).^13^, ^14^ These findings triggered the initiation of several clinical trials and their use in the hospital setting to treat severe COVID‐19 cases, usually combined with a macrolide antibiotic, such as azithromycin (AZI) or erythromycin (ERT). Although preliminary results showed reduced viral carriage in a small sample of patients with COVID‐19,^15^ these promising findings were not confirmed subsequently by larger studies, such as the RECOVERY trial.^16^ Moreover, an increased risk of cardiovascular mortality and heart failure was reported when OH‐CLQ was co‐administered with AZI.^17^ Another study conducted in 73 patients with SARS‐CoV‐2 infection found an average QT prolongation of 23 ms after 48 h of OH‐CLQ/AZI combined therapy.^18^

In the context of COVID‐19, the TdP liability of these medications used alone or in combination could aggravate the clinical state of the patients and explain the increased risk of mortality.

Consequently, the objective of the current work was to assess the proarrhythmic profile of CLQ, OH‐CLQ, AZI, and ERT, alone or in combination, using the full battery of in silico and in vitro assays proposed by the CiPA paradigm, which included cardiac ion current patch clamp assays, in silico drug simulations on virtual human ventricular myocytes, and hiPSC‐derived cardiomyocytes.

## METHODS

### Patch clamp assays on cardiac ion currents

Chinese hamster ovary cells stably transfected with the human cardiac isoforms of hERG, Na_V_1.5, Ca_V_1.2 (coexpressed with β2/α2δ1), K_V_4.3 (coexpressed with KChiP2.2), K_V_7.1/minK, or K_ir_2.1 were cultured in HAM/F12, whereas HEK 293 cells stably expressing HCN4 were cultured in a 1:1 mix of HAM/F12 and DMEM/F12 medium. All media were supplemented with 10% fetal bovine serum (FBS) and 1% penicillin/streptomycin at 37°C, 5% CO_2_, and saturated humidity.

For dataset #1, shortly before experimentation, the cells were harvested with Detachin 2–3 days after plating at a confluence of 70% and resuspended in phosphate‐buffered saline (PBS). All experiments were performed with automated patch‐clamp using QPatch (16X and HTX; Sophion Bioscience) at room temperature.

For dataset #2, before testing, cells in culture dishes were washed twice with Hank’s Balanced Salt Solution (HBSS) and treated with Accutase for ~ 20 min. Immediately before use in SP384PE, the cells were washed in HBSS to remove the Accutase and resuspended in HB‐PS. The drug formulations were loaded in a 384‐well polypropylene compound plate using an automated liquid handling system (Integra Assist Plus; Integra), and placed in the plate well of SyncroPatch 384PE (SP384PE; Nanion Technologies) immediately before application to the cells. The hERG and Na_V_1.5 experiments were run at 37°C, whereas Ca_V_1.2, K_V_7.1/minK, and HCN4 were run at room temperature.

The composition of extracellular and intracellular solutions, and the voltage protocols, as well as the positive controls used for each current assay are described in the Supplementary Methods. The hERG, Na_V_1.5, Ca_V_1.2, and K_V_4.3‐mediated currents were recorded and analyzed as recommended by the FDA.^19^

CLQ, OH‐CLQ, ERT, and AZI were supplied by Sigma‐Aldrich or Tocris. For CLQ and OH‐CLQ, stock solutions were prepared in water, and ERT and AZI were prepared in DMSO. The maximal final DMSO concentration was 0.3% in dataset #1 and 0.6% in dataset #2.

Five (dataset #1) or eight (dataset #2) concentrations of each drug were tested in three (dataset #) or four (dataset #2) replicates and are given in the Supplementary Methods.

Concentration‐response curves were analyzed and constructed with a sigmoidal two parameter equation:

$$\%\;\text{current}=100/\left[1+{\left(X/{\text{IC}}_{50}\right)}^{H}\right]$$

where *X* is the drug concentration, IC_50_ is the concentration producing half‐maximal inhibition, and *H* is the Hill coefficient.

For hERG experiments testing potential interaction between two drugs, the first drug was perfused until steady‐state current amplitude was reached and then increasing concentrations of the second drug were added, whereas maintaining the fixed concentration of the first drug. Drug combinations blocking 50% of *I*_Kr_ current were analyzed in an isobologram.^20^

### Human in silico action potential models

Human in silico drug trials were performed using the software Virtual Assay (version 3.2 2014 Oxford University Innovation Ltd.), which implements the population of cell model methodology,^21^, ^22^ similar to previous works.^23^, ^24^, ^25^

Three computational models of human ventricular electrophysiology were used: (i) the recently published ToR‐ORd model^26^; (ii) the model developed by the FDA within the CiPA initiative (ORd‐CiPA), and optimized for drug testing^27^; (iii) the O’Hara‐Rudy (ORd) model,^28^ starting from which both ToR‐ORd and ORd‐CiPA were developed. For each model, a control (“healthy”) population (~400 cells) and a high‐risk population (~100 cells) were constructed, for a total of six in silico populations of virtual human ventricular cells. All cell populations implement biological variability by randomly varying the main ionic current conductances of the AP models (Table S1), and are able to capture different drug‐induced phenotypes (i.e., repolarization abnormalities [RA] and depolarization abnormalities [DA]).^23^However, the high‐risk population has been specifically designed with a low repolarization reserve, to maximize the risk to develop RA.^24^

Simulations were performed for CLQ, OH‐CLQ, AZI, and ERT at multiple concentrations (from 0.5 to 1000 µM), using the two IC_50_ and Hill coefficient (H) datasets listed in Table 1 as input of a simple pore‐block model.^29^ Four drug combinations were also simulated (CLQ + AZI, CLQ + ERT, OH‐CLQ + AZI, and OH‐CLQ + ERT), by considering equal concentrations of each drug (from 0.5 to 1000 µM) and assuming a cumulative effect of their ion channel blocks.

**TABLE 1:** IC_50_ values (µM) of CLQ, OH‐CLQ, ERT, and AZI in patch clamp assays conducted on seven cardiac ion channels

Ion channel	hERG		Na_V_1.5 peak		Na_V_1.5 late		Ca_V_1.2 peak		Ca_V_1.2 ramp		Kir2.1	K_V_7.1/minK	HCN4		K_V_4.3/KChIP2
Current	*I*_Kr_		*I*_Na,peak_		*I*_Na, L_		*I*_CaL_		*I*_CaL_		I_K1_	*I*_Ks_	*I*_f_		*I*_to_
Dataset #	1	2	1	2	1	2	1	2	1	2	1	2	1	2	1
Temperature	RT	37°C	RT	37°C	RT	37°C	RT	RT	RT	RT	RT	RT	RT	RT	RT
CLQ	0.97 (1.10)	1.03 (1.01)	64.22 (0.80)	13.95 (0.77)	109.18 (0.69)	11.57 (0.73)	102.47 (0.72)	17.95 (0.70)	>1000 (NC)	>300 (NC)	4.98 (1.80)	>300 (NC)	227.95 (0.85)	96.19 (0.85)	>300 (NC)
OH‐CLQ	6.97 (0.96)	2.66 (0.94)	44.15 (0.85)	16.99 (0.80)	92.87 (0.82)	21.43 (0.82)	66.29 (0.88)	23.90 (0.65)	>1000 (NC)	>300 (NC)	44.15 (2.05)	>300 (NC)	372.28 (0.94)	>300 (NC)	>300 (NC)
ERT	393 (1.05)	137 (1.18)	>1000 (NC)	>1000 (NC)	NT	>1000 (NC)	>1000 (NC)	>1000 (NC)	>1000 (NC)	856 (0.83)	NT	>1000 (NC)	NT	>1000 (NC)	NT
AZI	763 (1.15)	439 (2.32)	1111 (0.87)	612 (1.32)	>1000 (NC)	>1000 (NC)	1391 (2.14)	771 (2.04)	>1000 (NC)	710 (1.02)	>1000	>1000 (NC)	>1000 (NC)	>1000 (NC)	>1000 (NC)

Note

Values in brackets are Hill coefficients.

Abbreviations: AZI, azithromycin; CLQ, chloroquine; ERT, erythromycin; IC_50_, half‐maximal inhibitory concentration; NC, not calculated; NT, not tested; OH‐CLQ, hydroxychloroquine; RT, room temperature.

Thirteen AP and calcium transient (CT) biomarkers were computed for the cells not displaying RA nor DA: AP duration at 40%, 50%, and 90% of repolarization (APD_40_, APD_50_, and APD_90_); AP triangulation, defined as the difference between APD_90_ and APD_40_ (Tri_90‐40_); maximum upstroke velocity (d*V*/d*t*_MAX_); peak voltage (*V*_peak_); resting membrane potential (RMP); CT duration at 50% and 90% of repolarization (CTD_50_ and CTD_90_); CT peak (CT_peak_); CT amplitude (CT_Amp_); diastolic intracellular Ca^2+^ concentration (Ca_i,dias_); and electromechanical window (EMw), defined as the difference between APD_90_ and CTD_90_.^24^ For ORd‐CiPA, we also computed the net charge carried by ionic currents (qNet), as described in ref. 27. However, it is worth noting that drugs’ effects on the hERG channel were represented with a simple pore‐block model, because the experimental data needed to parametrize the dynamic hERG model were not available. AP traces displaying a positive derivative after 150 ms were classified as RA, whereas AP traces with a *V*_peak_ < 0 mV were classified as DA. A more detailed description of the in silico drug trial methods is included in the Supplementary Methods.

### Human‐induced pluripotent stem cell‐derived cardiomyocytes

Experiments on hiPSC cardiomyocytes were adapted from a protocol previously described.^30^ E‐Plates for the xCELLigence RTCA CardioECR (ACEA Biosciences, Inc.) were coated using 10 μg/ml bovine fibronectin (Sigma Aldrich) dissolved in PBS (Thermofisher/Gibco 14040‐083 DPBS) for 1 h at 37°C or overnight at 4°C. Fibronectin was removed and immediately replaced with cellular media. A background recording was performed with media prior to seeding the cells. The hiPSC‐CM, iCell^2^ Cardiomyocytes (FujiFilm CDI) were stored as a frozen stock with at least 2.5 × 10^6^ cells/1 ml vial in liquid nitrogen (LN2). Cells were thawed and seeded at the density 50,000/well in iCell Plating Medium (FujiFilm CDI) according to the manufacturer’s recommendations. From 4 h post‐plating, cardiomyocytes were maintained on the E‐Plates in the Maintenance Medium (FujiFilm CDI), which was replaced every 48 h.

CLQ diphosphate salt, OH‐CLQ sulfate, AZI dihydrate, and ERT were purchased from Sigma Aldrich and dissolved in water. Four to 5 days after plating the cells, cells were incubated for 24 h with the drugs or drug combinations presented in Table 2. Each treatment was performed in triplicates. For combined treatment, we selected concentrations of CLQ (1 and 3 µM) and OH‐CLQ (3 and 10 µM) to be in the range of their *I*_Kr_ IC_50_. Dofetilide (6 nM), doxorubicin (1 µM), and isoproterenol (30 nM) were used as positive controls, and DMSO 0.1% as negative control along with the drug treatments in each plate. Acquisition of the data was performed according to RTCA CardioECR Instrument Operator’s Guide. Impedance data, cell index (CI), beat rate, and CI amplitude as well ECR data, sodium spike, and field potential duration (FPD) were recorded and analyzed by RTCA CardioECR software at baseline, 30 min, 1 h, 6 h, 12 h, and 24 h post‐treatment.

**TABLE 2:** Effects of CLQ, OH‐CLQ, AZI, and ERT, alone and in combination, on a selection of cardiovascular biomarkers, in a control (“healthy”) population of virtual human cardiomyocytes (Virtual Assay), and in hiPSC cardiomyocytes (RTCA‐ECR Cardio platform)

	CLQ	OH‐CLQ	AZI	ERT	CLQ + AZI	CLQ + ERT	OH‐CLQ + AZI	OH‐CLQ + ERT
In silico prediction in virtual human cardiomyocytes								
APD_90_	↑ 1 µM (33%)	↑ 5 µM (25%)	↑ 1 mM (37%)	↑ 300 µM (29%)	↑ 1 µM (34%)	↑ 1 µM (33%)	↑ 5 µM (25%)	↑ 5 µM (26%)
Tri_90‐40_	↑ 0.5 µM (28%)	↑ 3 µM (25%)	↑ 1 mM (26%)	↑ 300 µM (45%)	↑ 0.5 µM (29%)	↑ 0.5 µM (29%)	↑ 3 µM (25%)	↑ 3 µM (26%)
dV/dt_MAX_	↓ 10 µM (−29%)	↓ 30 µM (−29%)	↓ 1 mM (−33%)	<20%	↓ 10 µM (−31%)	↓ 10 µM (−30%)	↓ 30 µM (−31%)	↓ 30 µM (−29%)
CTD_90_	↑ 10 µM (20%)	↑ 30 µM (24%)	<20%	<20%	↑ 10 µM (20%)	↑ 10 µM (20%)	↑ 30 µM (24%)	↑ 30 µM (25%)
CT_peak_	↓ 10 µM (−28%)	↓ 30 µM (−41%)	↓ 1 mM (−35%)	<20%	↓ 10 µM (−28%)	↓ 10 µM (−28%)	↓ 30 µM (−41%)	↓ 30 µM (−41%)
EMw	↓ 0.5 µM (−29%)	↓ 3 µM (−24%)	↓ 300 µM (−24%)	↓ 300 µM (−49%)	↓ 0.5 µM (−29%)	↓ 0.5 µM (−29%)	↓ 3 µM (−25%)	↓ 3 µM (−25%)
In vitro hiPSC cardiomyocytes								
Test conc. (µM)	1–3–10–30	1–3–10–30	1–10–30–100	1–10–30–100	1–3 + AZI 10	1–3 + ERT 10	3 −10 + AZI 10	3–10 + ERT 10
EAD	3–10–30 µM	3–10–30 µM	None detected	30–100 µM	None detected	1–3 µM	None detected	3–10 µM
FPDc	↓ 1 µM (−19%)	↓ 1 µM (−24%)	↓ 30 µM (−20%)	↑10 µM (11%) ↓ 1 µM (−9%)	No change	NA	No change	NA
CI	↓ 30 µM (45%)	↓ 30 µM (40%)	↓ 30 µM (37%)	No change	No change	No change	No change	No change
Beat rate	↓ 3 µM (31%)	↓ 3 µM (24%)	↑ 10 µM (51%)	↓ 30 µM (46%)	↑ 1 µM (44%)	↓ 3 µM (31%)	↑ 3 µM (47%)	↓ 3 µM (37%)
CI amplitude	↓ 10 µM (42%)	↓ 3 µM (30%)	↓ 10 µM (56%)	Beat stop 100 µM	↓ 1 µM (48%)	↓ 3 µM (31%)	↓ 3 µM (58%)	↓ 10 µM (30%)
Spike amplitude	↓ 1 µM (43%)	↓ 3 µM (76%)	↓ 10 µM (43%)	↓ 30 µM (79%)	↓ 1 µM (35%)	↓ 1 µM (39%)	↓ 3 µM (48%)	↓ 3 µM (71%)

Note

Data are presented as lowest concentration with effect (i.e., at which changes higher than 20% compared with control conditions were observed). Percentage change observed at this concentration is indicated in brackets. For in silico populations, concentrations between 0.5 and 1000 µM were tested; median values were considered. See main text for AP and CT biomarker descriptions. For hiPSC cardiomyocytes, all concentrations producing EADs are listed; for FPDc, effects are only reported at concentrations without EADs.

Abbreviations: ↓, decrease; ↑, increase; AZI, azithromycin; CI, cell index; CLQ, chloroquine; EADs, early afterdepolarizations; ERT, erythromycin; EMw, electromechanical window; FPDc, field potential duration corrected for beat rate (Fridericia's formula used); hiPSC, human‐induced pluripotent stem cell; NA, not available; NC, not calculated; NT, not tested; OH‐CLQ, hydroxychloroquine.

### Integrated QT risk assessment of CLQ, OH‐CLQ, ERT, and AZI

Composite plots (QT‐ograms) were constructed using free concentrations of each drug and percentage change in QT‐related biomarkers generated in the different assays (i.e., *I*_Kr_ current from both patch clamp datasets and APD_90_ as well as EMw simulations in control population of virtual human cardiomyocytes; from Table 2). For CLQ, the percentage of corrected QT (QTc) increase in man were extracted from the clinical study GSK protocol TAF106491 (clinicaltrials.gov identifier NCT00871156)^31^ and were calculated assuming a baseline QTc of 400 ms. Free plasma concentration (FPC) of each drug was estimated based on pharmacokinetic data retrieved from the PharmaPendium database^32^ or literature references^33^, ^34^, ^35^ and was considered to be 0.4 µM for a 500 mg dose of CLQ, 0.5 µM for a 400 mg dose of OH‐CLQ, and 0.5 µM for a 500 mg dose of AZI and ERT.

## RESULTS

### Effects of CLQ, OH‐CLQ, ERT, and AZI and their combinations on cardiac ion currents

Table 1 presents the IC_50_ values of CLQ, OH‐CLQ, ERT, and AZI in patch clamp assays conducted on seven cardiac ion currents under different experimental conditions (datasets #1 and #2). CLQ and OH‐CLQ inhibited hERG‐mediated *I*_Kr_ current with IC_50_ values around 1 µM for CLQ, and between 3 and 7 µM for OH‐CLQ. Both drugs also inhibited Na_V_1.5 and Ca_V_1.2‐mediated currents, with IC_50_ values ranging from 12 to 109 µM, although more variability was observed between the two datasets than for *I*_Kr_. A blockade of K_ir_2.1‐mediated current *I*_K1_ was also noted, CLQ being more potent (IC_50_: 5 µM) than OH‐CLQ (IC_50_: 44 µM). Very weak if no activity was observed on the other ion currents (IC_50_ >100 to 1000 µM).

AZI and ERT exhibited only weak activity on the different cardiac ion currents, most IC_50_ values being greater than 300 or 1000 µM. The most pronounced inhibitory activity was noted on *I*_Kr_ current for ERT (IC_50_: 137 µM).

**FIGURE 1:**
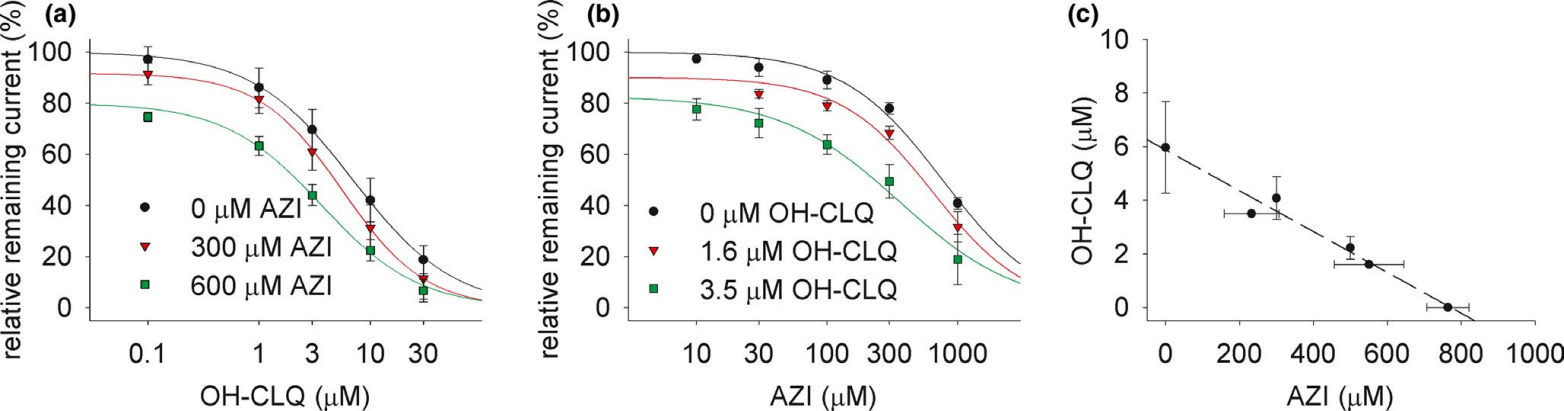
Concentration‐response curves of hERG‐mediated current (*I*_Kr_) amplitude for hydroxychloroquine (OH‐CLQ) (a) and azithromycin (AZI) (b) tested alone or in combination. Isobologram for drug combinations of AZI and OH‐CLQ (c) was constructed. Data best fit with a linear equation, indicating no synergistic or antagonistic interaction. Values are mean ± SD

When testing drug combinations of OH‐CLQ and AZI, OH‐CLQ IC_50_ values in the presence of 0, 300, or 600 µM AZI and AZI IC_50_ values in the presence of 0, 1.6, or 3.5 µM OH‐CLQ were determined (Figure 1,a,b). Combinations of AZI/OH‐CLQ concentrations were plotted in an isobologram and fit with a linear equation, indicating an additive, but no synergistic or antagonistic effect of both drugs (Figure 1,c).

### Effects of CLQ, OH‐CLQ, ERT, and AZI and their combinations on human in silico drug trials

**FIGURE 2:**
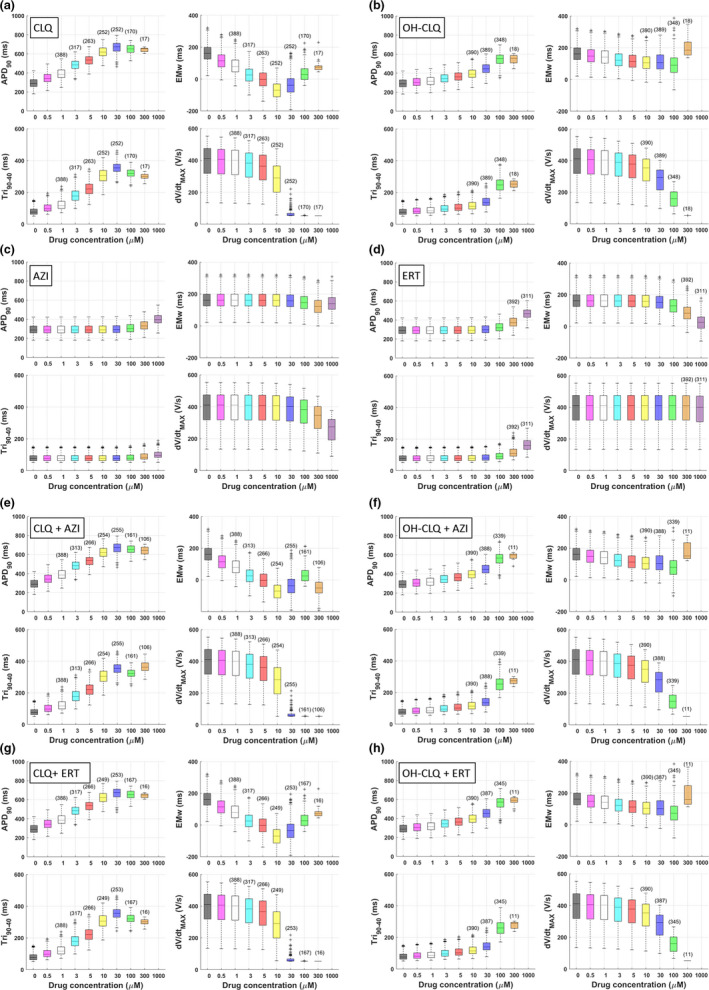
Concentration‐response curves of a selection of AP and calcium transient (CT) electrophysiological biomarkers, simulated in a control (healthy) population of virtual cell models treated with chloroquine (CLQ), hydroxychloroquine (OH‐CLQ), azithromycin (AZI), and erythromycin (ERT), and their combinations. See the main text for biomarker descriptions. In each boxplot, the central mark is the median of the population, box limits are the 25th and 75th percentiles, and whiskers extend to the most extreme data points not considered outliers, which are plotted individually as separate crosses. Only the models not displaying repolarization abnormalities (RAs) or depolarization abnormalities (DAs) are plotted for each concentration (the number of cells is listed in brackets on top of each boxplot). CLQ and, with smaller magnitude, OH‐CLQ affected all biomarkers even at low concentrations, whereas AZI and ERT only showed effects at high concentrations. Combinations of an antimalarial with an antibiotic did not worsen the response. EMw, electromechanical window

**FIGURE 3:**
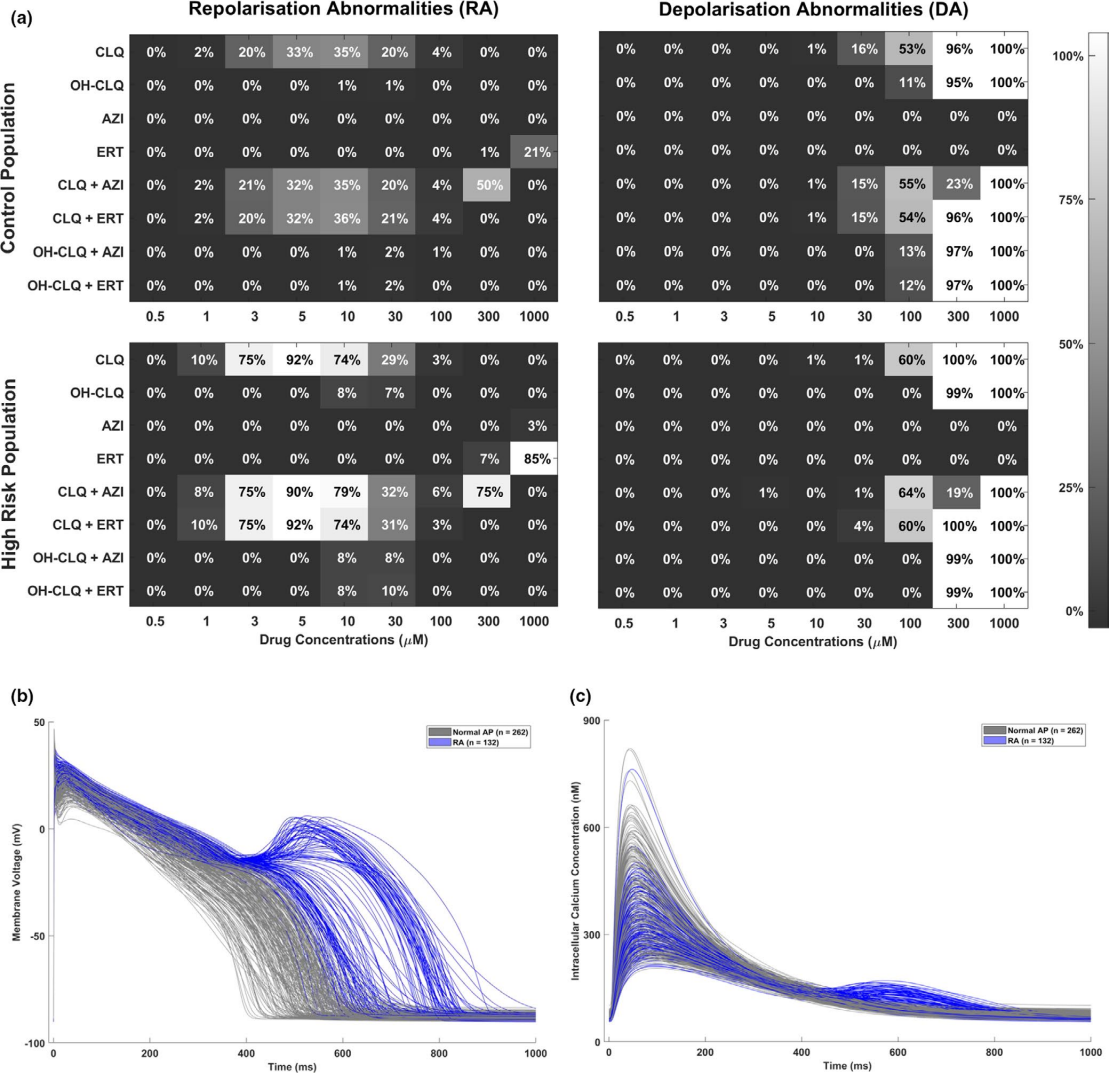
(a) Summary of the drug‐induced abnormalities (repolarization abnormalities [RAs], left; depolarization abnormalities [DAs], right) observed in silico for each of the tested drugs and combinations, in the control (top) and high‐risk (bottom) populations. RAs appear at low concentrations of chloroquine (CLQ), the RA prevalence is not increased by combination with an antibiotic but is higher in the high‐risk population. DAs only occur at higher concentrations. (b, c) AP and calcium transient (CT) traces obtained for the control population when simulating CLQ at 5 µM. The in silico models displaying RAs are highlighted in blue, whereas the ones showing normal—even though prolonged—repolarization, are plotted in gray. About half of the cells showed RA. AZI, azithromycin; ERT, erythromycin; OH‐CLQ, hydroxychloroquine

**FIGURE 4:**
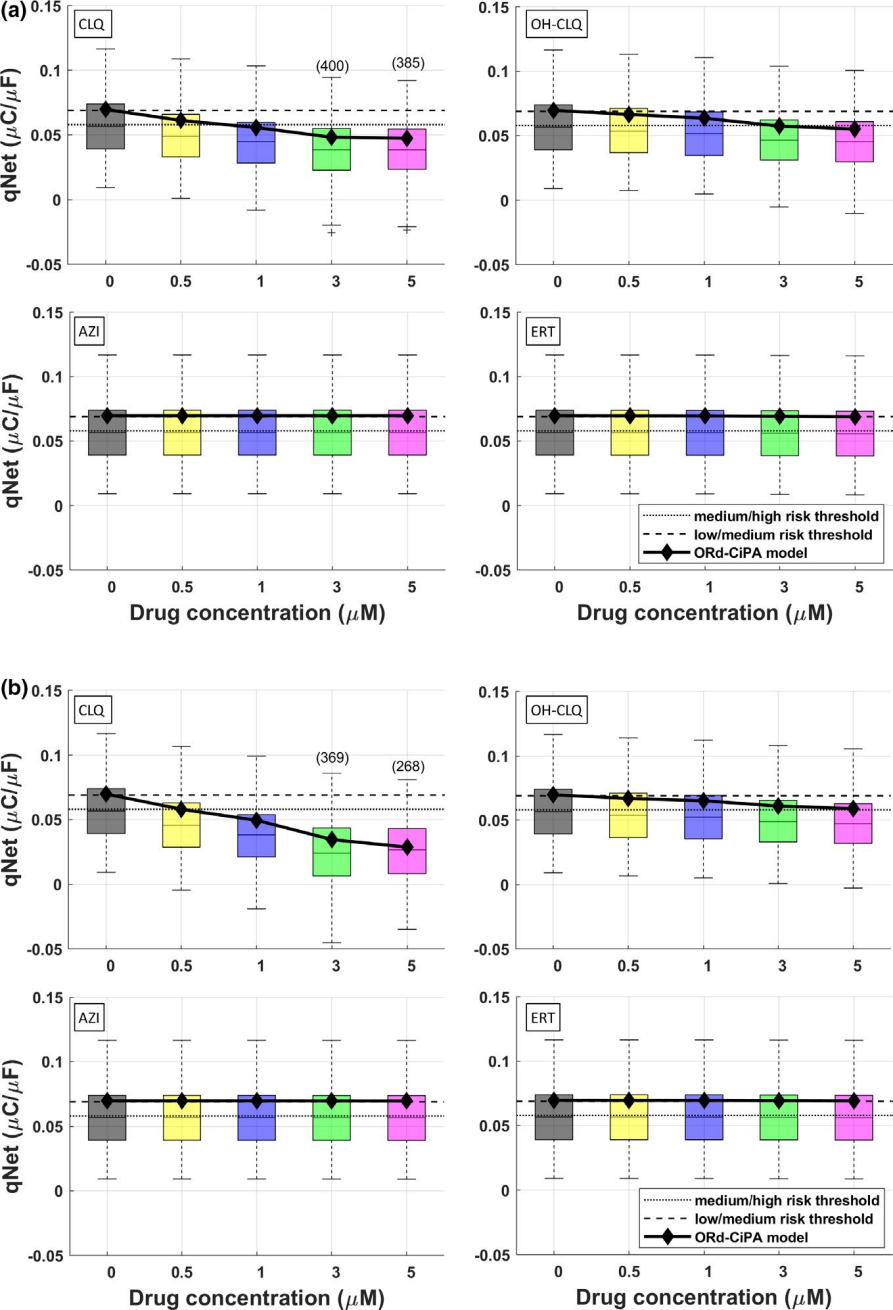
Changes observed in the qNet biomarker following CLQ, OH‐CLQ, AZI, and ERT application. Simulation results are shown for the ORd‐CiPA model (black diamonds) and the corresponding control population (boxplots). (a) Simulation results obtained using the half‐maximal inhibitory concentration (IC_50_)/Hill coefficients of dataset #1 from Table 1. (b) Simulation results obtained using the IC_50_/Hill coefficients of dataset #2 from Table 1. In each panel, the thresholds suggested by Li et al.^36^ to separate low/medium/high risk drugs are shown as black dotted and dashed lines. CLQ would be classified as high‐risk, OH‐CLQ as medium risk, and AZI and RT as low risk drug. AZI, azithromycin; CLQ, chloroquine; ERT, erythromycin; OH‐CLQ, hydroxychloroquine; ORd‐CiPA, O’Hara‐Rudy Comprehensive In Vitro Proarrhythmia Assay

Simulation results are overall consistent across the six in silico cell populations and the two IC_50_/H datasets, both for drug‐induced changes in AP and CT biomarkers, and occurrence of RA and DA. Figures 2, 3, 4 and Table 2 show a selection of the results obtained with the ToR‐ORd model and the dataset #1 (from Table 1). The full set of results obtained with the three computational models, the two datasets, and the two population types (control and high‐risk) is given in Table S2 and Figure S1.

CLQ‐induced noticeable concentration‐dependent changes in most AP (in particular APD_90_ and Tri_90‐40_) and CT biomarkers, even at the lower tested concentrations (Figure 2,a), while reducing the EMw. The d*V*/d*t*_MAX_ and *V*_peak_ (not shown) were substantially decreasing at high concentrations (>30 μM), due to the large I_Na_ block that compromises the AP upstroke, thus causing a slight inversion in all biomarker trends. Similar effects were observed when simulating OH‐CLQ (Figure 2,b), even though of a smaller magnitude. Both AZI and ERT had very little effect at lower concentrations tested (<30 μM), but showed increased APDs, Tri_90‐40_, and CTDs, and decreased EMw at higher ones (Figure 2,c,d), with ERT showing a larger effect than AZI. The addition of AZI or ERT to either CLQ or OH‐CLQ did not greatly affect the biomarker changes observed for CLQ and OH‐CLQ alone (Figure 2,e–h). These results are confirmed in Table 2, showing the minimum tested concentration at which drug‐induced changes for a selection of in silico AP and CT biomarkers were greater than 20% compared with control conditions.

Figure 3a summarizes the occurrence of RA and DA in the in silico populations. CLQ induced RA from 1 μM, in up to 35% and 92% of the cells, for the control and high‐risk populations, respectively. OH‐CLQ also induced RA, but from 10 μM and in a smaller fraction of the population (up to 8%). AZI only showed RA in the high‐risk population, at the maximum tested concentration (1000 μM). ERT showed RA from 300 μM, in up to 85% of the cells in the high‐risk population. DA were observed only for CLQ and OH‐CLQ, at high concentrations (>300 μM), with no relevant differences between the control and high‐risk populations. Results for drug combinations were similar to the ones obtained for CLQ and OH‐CLQ alone. As representative example, AP and CT traces for 5 μM CLQ are shown in Figure 3b,c. The CiPA qNet biomarker showed a notable reduction for CLQ and OH‐CLQ, while remaining almost unchanged for AZI and ERT (Figure 4). Based on the thresholds suggested in ref. 36, CLQ could be classified as high risk, OH‐CLQ as medium risk, and AZI and ERT as low risk drugs.

### Effects of CLQ, OH‐CLQ, ERT, and AZI, and their combinations on hiPSC‐CM function

Table 2 shows the changes observed in human iPSC‐cardiomyocytes in all recorded parameters in response to CLQ, OH‐CLQ, AZI, and ERT, alone or in combination. Minimum concentrations that produced more than 20% change (considered as threshold for biological relevance in this model)^37^ as compared with DMSO are reported. CLQ and OH‐CLQ caused concentration‐dependent beat rate decrease. Negative chronotropic effects were also observed with ERT, whereas AZI exhibited positive chronotropic effects. CLQ and OH‐CLQ were associated with early afterdepolarizations (EADs) at nearly all concentrations tested, whereas for ERT, EADs were only noted at high concentrations. No EADs were noted with AZI. For the concentrations where EADs were not present, the change in FPD was not higher than 20% as compared with DMSO. Combining CLQ with 10 µM ERT showed similar effects on cardiomyocytes as with CLQ applied alone, but with EADs observed even at lower concentrations than with CLQ alone. When CLQ was combined with AZI, EADs were not observed any longer and instead of a negative, a positive chronotropy was observed. Similar changes were observed when OH‐CLQ was combined with AZI or ERT.

### Integrated QT risk assessment of CLQ, OH‐CLQ, ERT, and AZI

**FIGURE 5:**
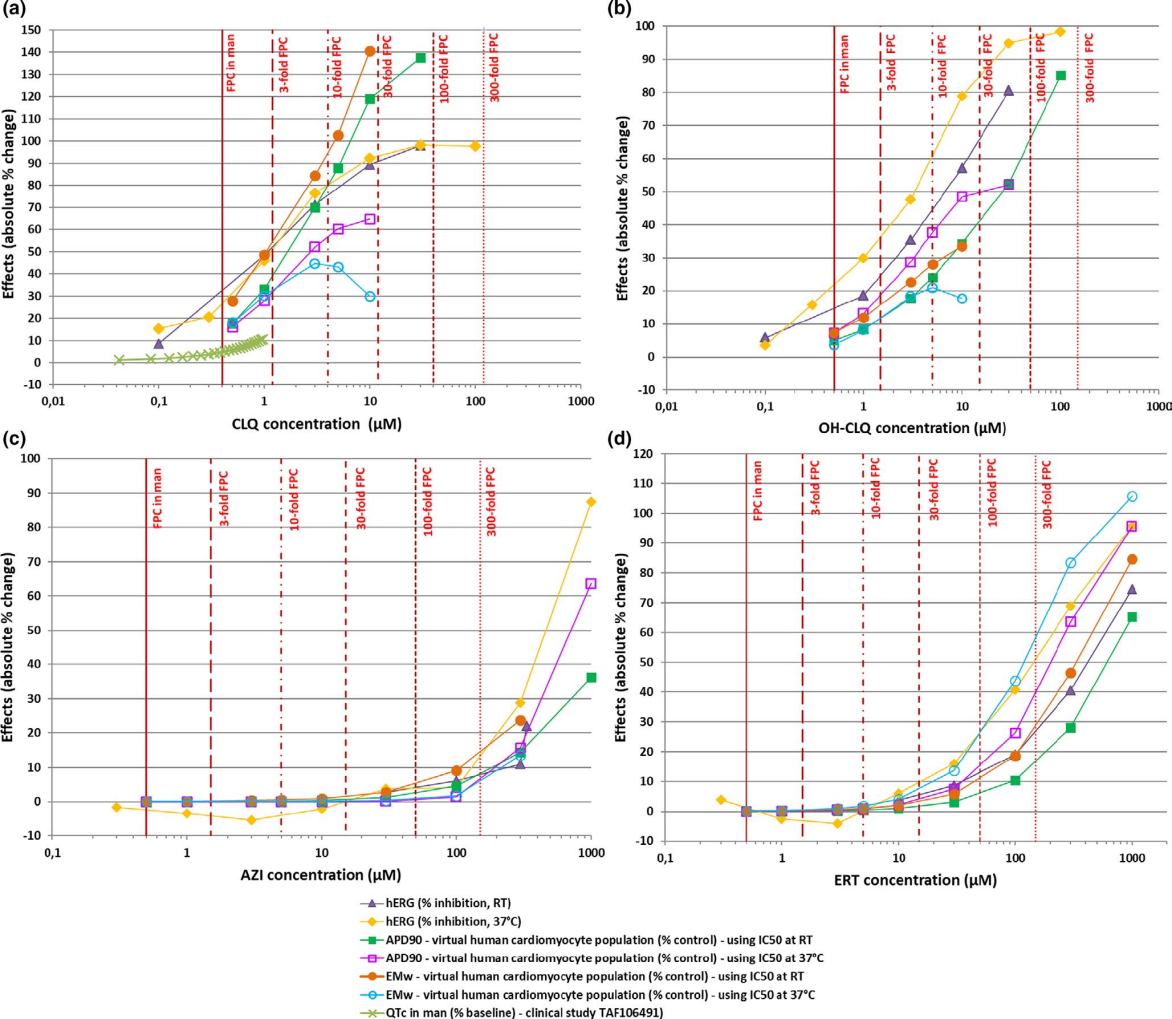
Composite “QT‐ograms” integrating concentration‐responses to CLQ, OH‐CLQ, AZI, and ERT generated in hERG patch clamp assays at room temperature (dataset #1, triangles) or 37°C (dataset #2, diamonds), in virtual human ventricular myocyte population using ion channel half‐maximal inhibitory concentration (IC_50_) values from dataset #1 (plain squares for APD_90_, plain circles for electromechanical window [EMw]) or dataset #2 (open squares for APD_90_, open circles for EMw). Effects are expressed as absolute percentage change from control conditions (percentage inhibition for hERG, percentage increase in APD_90_, or percentage decrease in EMw for virtual cardiomyocytes). For CLQ, percentage QTc increase in man (crosses) are extracted from the clinical study GSK protocol TAF106491 (clinicaltrials.gov identifier NCT00871156) and were estimated assuming a baseline corrected QT (QTc) of 400 ms. Vertical bars represent therapeutic free plasma concentration (FPC) of each drug and multiples. AZI, azithromycin; CLQ, chloroquine; ERT, erythromycin; OH‐CLQ, hydroxychloroquine; RT, room temperature

The CLQ “QT‐ogram” (Figure 5a) illustrates that biologically relevant changes (>10–20%) in QT‐related biomarkers can occur below or close to the FPC (<3‐fold). All nonclinical data are consistent together and also fit with the QT prolongation observed in man in the same concentration range. Similar profile is noted for OH‐CLQ (Figure 5b), with slightly larger safety ratios based on in silico simulations. In contrast, AZI and ERT QT‐ograms show more favorable cardiac safety profiles, the first relevant effects appearing around 30‐fold the FPC for ERT (Figure 5d) and above 300‐fold the FPC for AZI (Figure 5c).

## DISCUSSION

The present work “road‐tested” the new CiPA paradigm,^1^, ^2^, ^3^, ^4^ aiming at assessing the proarrhythmic risk of drugs using human‐based models, with CLQ and OH‐CLQ, two long‐established antimalarials that were repurposed for treatment of COVID‐19 during the first wave of the pandemic in 2020. The experiments consisted of a panel of in silico and in vitro assays (i.e., patch clamp assays on human cardiac ion currents, integration of these data within in silico reconstructions of human ventricular electrophysiology, and confirmation of proarrhythmic effects in hiPSC ventricular myocytes). The benefit of generating this set of robust nonclinical data is that it can be used to inform clinicians on the proarrhythmic risk under specific clinical circumstances, such as in patients at higher risk, and calculate an accurate safety margin associated with such a risk.

Patch clamp assays conducted on mammalian cells transfected with genes encoding for human ion channels confirmed that both CLQ and OH‐CLQ are *I*_Kr_ current inhibitors. Our IC_50_ values in the micromolar range are in agreement with the previously published value for CLQ (2.5 µM).^10^ Our data also confirm that CLQ can inhibit *I*_K1_ current regulating the late phase of ventricular repolarization, with an IC_50_ of 4.98 µM fitting with the value previously reported (8.7 µM).^38^ Blockade of *I*_K1_ current has also been associated with long QT^39^ and could therefore exacerbate *I*_Kr_ blockade‐related risk at higher CLQ dose regimens, as have been deployed in some COVID‐19 clinical trials.^40^ In contrast, *I*_K1_ current inhibition by OH‐CLQ would only occur at concentrations 10‐fold higher than CLQ, far above clinically relevant exposures. Both drugs also appear to block *I*_Na_ and *I*_CaL_ currents, although some variability was noted between the two datasets (IC_50_ values: 14–64 µM for *I*_Na_, 12–109 µM for *I*_CaL_). Blockade of sodium and calcium currents have been shown to potentially mitigate/attenuate the risk of QT prolongation resulting from *I*_Kr_ inhibition.^2^, ^41^ However, this potential mitigating effect would occur at free plasma concentrations that are above the therapeutic levels for malaria treatment (0.4–0.6 µM), and therefore, is unlikely to play any significant role. Overall, OH‐CLQ appeared slightly less potent than CLQ in terms of ion channel inhibitory activities, across all currents evaluated. In contrast with the two antimalarial drugs, the two macrolides, ERT and AZI, exhibited only weak if no inhibitory action on the ion currents. We also demonstrated the absence of synergistic interaction of AZI and OH‐CLQ on hERG current. We acknowledge some deviations from the current “best practice” considerations^9^ in our ion channel experiments, such as the lack of drug concentration solution analyses or the use of room temperature for channels other than hERG and Na_V_1.5. However, it was previously demonstrated that drug formulation analysis did not affect hERG potency estimation for the majority of a set of 99 drugs tested.^42^ Temperature sensitivity of ion channel assays is variable and generally depends on physicochemical properties of each compound. As observed in our work, ERT hERG activity was previously shown to be dependent on temperature, with higher potency noted at near physiological temperature.^43^ As the influence of temperature and possibly other experimental conditions on the other currents have been less studied, we cannot exclude a potential modest underestimation of the potencies reported in this work.

The IC_50_ values generated in the patch clamp assays were then used to generate in silico simulations of electrophysiological effects of CLQ, OH‐CLQ, AZI, and ERT, in 6 different populations of virtual human ventricular myocytes, using the Virtual Assay tool, which offers the advantage of testing drugs in a population of cell models mimicking the interindividual variability inherent to a population of patients.^21^ Moreover, the cell models can be calibrated to represent patients at higher cardiovascular risk. Using Virtual Assay, despite IC_50_ differences between the two patch clamp datasets, both antimalarial drugs consistently increase action potential durations, an in vitro surrogate biomarker of QT, and affect several TdP indicators, such as EMw, qNet, and trigger repolarization abnormalities. This is particularly true for high‐risk populations. No major effects were observed for AZI nor ERT. Using qNet, we were able to classify the four drugs in terms of their TdP risk. However, it is worth noting that the qNet biomarker is highly sensitive to variation in the model parameters (distribution in the population compared with single model results). Therefore, thresholds based on percent changes rather than absolute values might be more appropriate for risk predictions. In addition, the qNet biomarker was also observed to be highly sensitive to the stimulation protocol and number of beats (e.g., variations up to 200% were observed when simulating virtual cells at 500 vs. 50 beats).

The electrophysiological changes predicted by the in silico simulations were further confirmed in hiPSC‐derived cardiomyocytes. In this in vitro assay, the observation of EADs from 3 µM of CLQ and OH‐CLQ confirms the in silico predictions obtained in Virtual Assay and indicates that both drugs have the potential to trigger proarrhythmic events like TdP. The presence of these EADs rendered difficult the extraction of the field potential duration, the surrogate marker of QT in this model. Interestingly, when CLQ or OH‐CLQ was combined with AZI, EADs were no longer observed; the reason for this phenomenon is unknown. The sodium spike amplitude decrease, also noted with both antimalarial drugs from 3 µM, could be related to the sodium blockade and is in agreement with the d*V*/d*t*_MAX_ and *V*_peak_ decrease predicted in silico. Overall, the data obtained with CLQ and OH‐CLQ in hiPSC cardiomyocytes in our study are in accordance with the QT and QRS interval increases previously reported in in vitro and in vivo rabbit studies.^11^

Based on the QT‐ograms, the risk of QT prolongation and TdP events appears clear for both CLQ and OH‐CLQ at the achieved free plasma concentrations in the clinical studies reported.^31^, ^32^, ^33^, ^34^, ^35^ This risk is revealed through reports of prolonged QT and/or TdP in patients treated with CLQ or OH‐CLQ in the FDA Adverse Events Reporting System database.^32^ Moreover, clinical trials conducted with CLQ showed QTc prolongation in healthy subjects^12^ as well as with OH‐CLQ/AZI in patients with COVID‐19.^18^ Because ERT, and more importantly AZI, show larger safety margins with regard to QT prolongation risk, their contribution to QT prolongation or TdP events are expected to be minimal when combined with CLQ or OH‐CLQ, as applied in the first wave of COVID‐19 or if considered for malaria treatment.^44^ This was confirmed in virtual ventricular cell models and hiPSC‐derived cardiomyocytes, in which the combination of ERT or AZI with CLQ or OH‐CLQ did not seem to aggravate the proarrhythmic indicators at clinically relevant concentrations.

It is important to consider that our integrated risk assessment is based on peak plasma levels obtained after single administration (i.e., maximum plasma concentration [*C*_max_]), and that the drugs could potentially accumulate into cardiac tissue after repeat administration of high doses of CLQ or OH‐CLQ over a few successive days, as applied in the treatment of malaria or in patients with COVID‐19. Moreover, in addition to the well‐known increased cardiovascular risk of some patient populations, such as elderly people, diabetics, or subjects with pre‐existing heart disease, it has now been clearly demonstrated that COVID‐19 itself triggers, even in young subjects, pathological situations, such as electrolyte imbalance^45^ and cardiovascular disorders, such as myocardial injury, arrhythmias, and acute coronary syndrome.^46^

Although we acknowledge the limitations of our work, because no in vivo QT data were generated as required by the ICH S7B guidance and because some experimental conditions for patch clamp assays slightly deviated from the current and emerging best practice considerations, all in vitro and in silico data generated are consistent together as well as with available clinical findings. In conclusion, our integrated nonclinical CiPA dataset confirmed that, at therapeutic plasma concentrations relevant for malaria or off‐label use in COVID‐19, CLQ and OH‐CLQ use is associated with a risk of QT prolongation and TdP, which is higher in populations carrying predisposing factors but not worsened with macrolide combination. This assessment reinforces the FDA/European Medicines Agency (EMA) recommendations issued during the first wave of COVID‐19 for using these drugs only in the context of a clinical trial or in a hospital setting, where this risk is more optimally managed through close cardiac monitoring and precautionary measures, whereas their use outside this context may not offer a favorable benefit‐risk balance in patients with COVID‐19.

## CONFLICT OF INTEREST

A.D., V.G., and J.‐P.V. are employees and stockholders of UCB Biopharma SRL. D.L. and M.A. are employees and stakeholders of Eli Lilly and Company. E.I.R., K.W.C., and K.A.B. are employees and stakeholders of GSK. Y.K., C.W., and J.K. are employees of Charles River Laboratories. S.H. and J.C. are employees of B’SYS GmbH. W.S.R. and M.H. are employees and stakeholders of Certara. E.P. and B.R. have worked to develop the Virtual Assay software, which is available for both commercial and academic research through Oxford University Innovation (OUI). All other authors declared no competing interests for this work.

## AUTHOR CONTRIBUTIONS

A.D., K.A.B., S.H., J.K., E.P., E.I.R., and W.S.R. wrote the manuscript. K.W.C., V.G., S.H., J.K., B.R., D.L., and J.‐P.V. designed the research. W.D.A., J.C., Y.K., I.L., E.P., C.T., and C.W. performed the research. M.A., W.D.A., V.G., M.H., and S.H. analyzed data.

## Supporting information

Supplementary Material

Table S1

Table S2

Figure S1
