# Supplementary material for: Applying the CiPA approach to evaluate cardiac proarrhythmia risk of some antimalarials used off‐label in the first wave of COVID‐19
Source: Clin Transl Sci. 2021 Apr 9;14(3):1133–46. doi: 10.1111/cts.13011 (PMC8014548; doi:10.1111/cts.13011)
Supplement: Supplementary file 1 — Supplementary Material [file CTS-14-1133-s003.docx]

**SUPPLEMENTARY METHODS**

***Patch clamp assays on cardiac ion currents***

Cell lines:

All cell lines used used in Dataset #1 were stably transfected with cDNA encoding for the human isoform of the different tested ion channels. Transfection was done by lipofection, and integration of the genes into the host cell’s genome was proven by PCR.

For Dataset #2, cell lines stably expressing the different tested ion channels were constructed as described previously^1^. Linearized plasmids were transfected by nucleofection into HEK293 or CHO-K1 cells. Cells containing chromosomally integrated expression construct were selected with antibiotics (Blasticidin/Zeocin for hERG, K_V_7.1/mink and HCN4; G418 for Na_V_1.5; Blasticidin/G418/Zeocin/Hygromycin for Ca_V_1.2). The absence of mycoplasma species in these cell lines were confirmed with the MycoAlert Kit (Lonza Rockland, Inc.). Single cells were deposited in 96-well plates by flow sorting. Clones were selected in an electrophysiology screen and final clone was verified by western blot and functionally validated by electrophysiological assays.

Voltage protocols for patch clamp assays were conducted as follows:

| ***Current*** | ***Dataset #1*** | | ***Dataset #2*** | |
| --- | --- | --- | --- | --- |
| hERG | 500-ms step pulse to +40 mV and 100-ms Ramp from +40 mV to -80 mV from the holding potential -80 mV  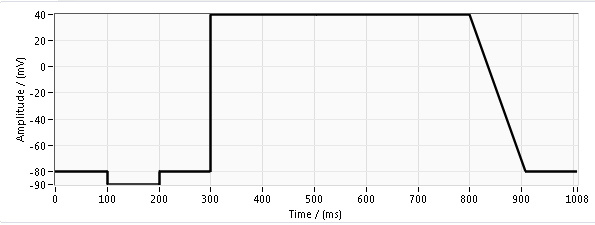 | | | |
| Na_V_1.5 | 200-ms conditioning pulse to -120 mV, 40-ms test pulse to -15 mV, 200-ms pulse to +40 mV and 100-ms Ramp from +40 mV to -95 mV from the holding potential -95 mV  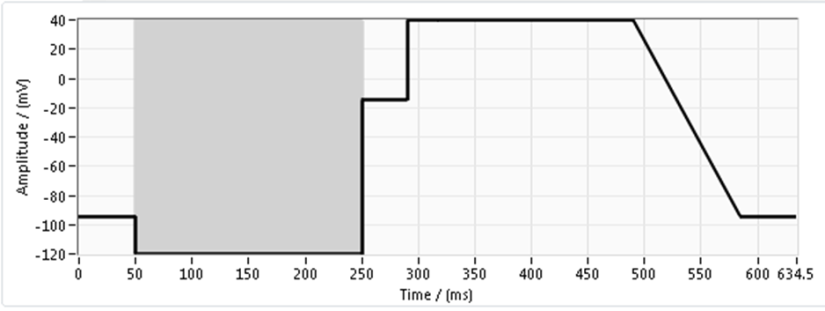 | | | |
| Ca_V_1.2 | 40-ms test pulse to 0 mV, 200-ms pulse to +30 mV and 100-ms Ramp from +30 mV to -80 mV from the holding potential -80 mV  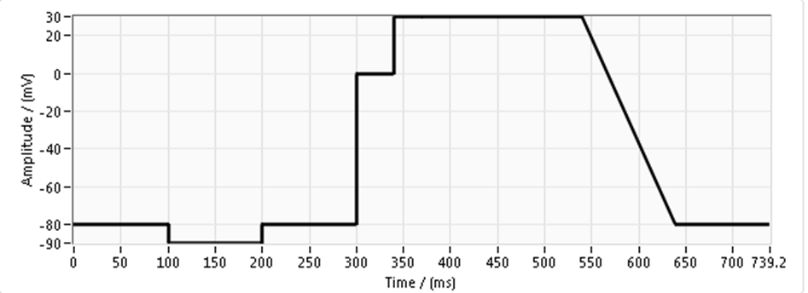 | | | |
| K_V_7.1/minK | Not done | 1000-ms step pulse to +40 mV and 500-ms step to -40 mV from the holding potential -80 mV  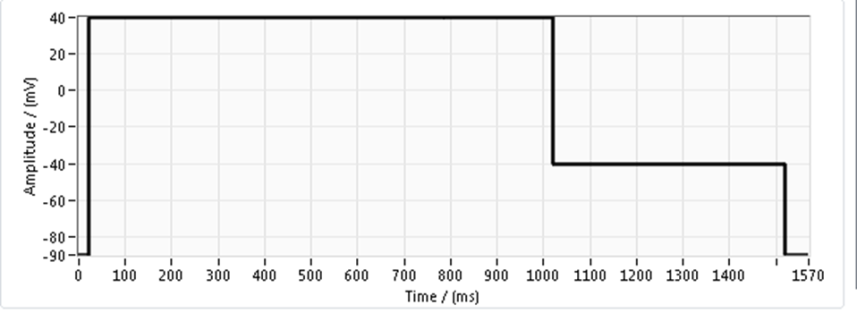 | | |
| HCN4 | 2000 ms step pulse to -120 mV from the holding potential -40 mV  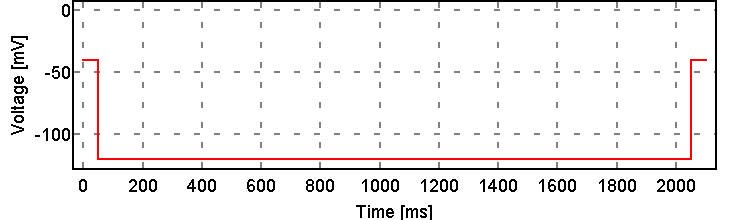 | | 2000-ms step pulse to -120 mV and 500-ms step pulse to +20 mV from the holding potential -30 mV  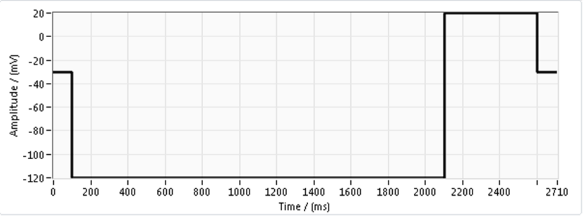 | |
| Kir2.1 | 1000 ms ramp from -120 mV to +60 mV from the holding potential -60 mV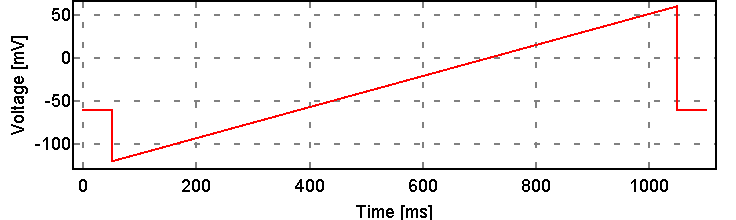 | | | Not done |
| K_V_4.3 | 500 ms step pulse to +20 mV from the holding potential -80 mV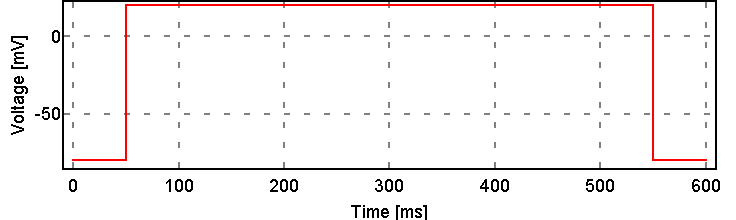 | | | Not done |

Composition of the extracellular and intracellular solutions for both Datasets are described below:

|  | **Dataset #1** | **Dataset #2** |
| --- | --- | --- |
| **Extracellular solution** | | |
| ***Chemical*** | ***Concentration (mM)*** | ***Concentration (mM)*** |
| NaCl | hERG, Na_V_1.5, K_ir_2.1, HCN4: 137  Ca_V_1.2: 100  K_V_4.3: 140 | hERG, Na_V_1.5, Ca_V_1.2, K_V_7.1/minK: 137  HCN4: 100 |
| KCl | hERG, Na_V_1.5, Ca_V_1.2, K_ir_2.1, HCN4: 4  K_V_4.3: 1 | hERG, Na_V_1.5, Ca_V_1.2, K_V_7.1/minK: 4  HCN4: 40 |
| CaCl_2_ | hERG, Na_V_1.5, K_ir_2.1, HCN4, K_V_4.3: 1.8  Ca_V_1.2: 5 | hERG, Na_V_1.5, K_V_7.1/minK: 3.8  Ca_V_1.2: 2  HCN4: 4.8 |
| MgCl_2_ | All channels: 1 | All channels: 1 |
| HEPES | All channels: 10 | All channels: 10 |
| D-Glucose | hERG, Na_V_1.5, K_ir_2.1, HCN4, K_V_4.3: 10  Ca_V_1.2: 5 | All channels: 10 |
| Sorbitol | Ca_V_1.2 only: 2.5 | - |
| NMDG | Ca_V_1.2 only: 40 | - |
| pH | hERG, Na_V_1.5, K_ir_2.1, HCN4, K_V_4.3: adjusted to 7.4 with NaOH  Ca_V_1.2: adjusted to 7.4 with HCl | Up to reach pH 7.4 |
| **Intracellular solution** | | |
| KCl | hERG, K_ir_2.1: 130  K_V_4.3: 30 | hERG, K_V_LQT1/minK, HCN4: 70 |
| NaCl | Na_V_1.5, HCN4: 10  Ca_V_1.2: 2.5  K_V_4.3: 7 | - |
| CaCl_2_ | hERG, K_ir_2.1: 2  Ca_V_1.2: 0.05  HCN4: 0.2 | - |
| KF | - | hERG, K_V_7.1/minK, HCN4: 70 |
| CsCl | - | Na_V_1.5, Ca_V_1.2: 50 |
| CsF | Na_V_1.5, HCN4: 135  K_V_4.3: 100  Ca_V_1.2: 33.75 | Na_V_1.5, Ca_V_1.2: 90 |
| CsMeS03 | Ca_V_1.2: 81 | - |
| MgCl_2_ | hERG, K_ir_2.1: 4  Ca_V_1.2: 3.375  K_V_4.3: 1 | All channels: 2 |
| EGTA | hERG, Na_V_1.5, K_ir_2.1, HCN4, K_V_4.3: 5  Ca_V_1.2: 8.75 | hERG, K_V_7.1/minK, HCN4: 2.5  Na_V_1.5, Ca_V_1.2: 5 |
| Na_2_ATP | hERG, K_ir_2.1: 4  Ca_V_1.2: 3 | - |
| Pyruvate, oxaloacetate, creatine, phosphocreatine | Ca_V_1.2 only: 3.75 of each | - |
| cAMP | HCN4 only: 0.01 | - |
| HEPES | hERG, Na_V_1.5, K_ir_2.1, HCN4: 10  Ca_V_1.2: 20.5  K_V_4.3: 5 | All channels: 10 |
| pH | Na_V_1.5: adjusted to 7.3 with KOH  HCN4: adjusted to 7.3 with CsOH  hERG, K_ir_2.1, K_V_4.3: adjusted to 7.2 with KOH  Ca_V_1.2: adjusted to 7.2 with CsOH | 7.2 (adjusted with KOH or CsOH for Na_V_1.5 and Ca_V_1.2) |

Positive controls used in each assay are given below:

| **Ion channel assay** | **Dataset # 1** | **Dataset # 2** |
| --- | --- | --- |
| hERG | E-4031 (100nM) | Cisapride (0.001, 0.003, 0.01, 0.03, 0.1, 0.3, 1 and 3µM) |
| Na_V_1.5 | Quinidine (60µM) | Lidocaine (1, 3, 10, 30, 100, 300, 1000 and 3000µM) |
| Ca_V_1.2 | Nifedipine (1µM) | Nifedipine (0.001, 0.003, 0.01, 0.03, 0.1, 0.3, 1 and 3µM). |
| K_V_4.3 | Dapoxetine (30µM) | NA |
| K_V_7.1/minK | NA | Chromanol (0.1, 0.3, 1, 3, 10, 30, 100 and 300µM) |
| K_ir_2.1 | ML 133 (10µM) | NA |
| HCN4 | Ivabradine (10µM) | ZD-7288 (0.03, 0.1, 0.3, 1, 3, 10, 30 and 100µM) |

Test concentrations used for each drug were as below (in µM):

| **Drug** | **Dataset # 1** | **Dataset # 2** |
| --- | --- | --- |
| CLQ | hERG : 30, 10, 3, 1, 0.1  Na_V_1.5, Kir2.1, HCN4, K_V_4.3: 300, 100, 30, 10, 3  Ca_V_1.2 : 1000, 300, 100, 30, 10 | All assays :  300, 100, 30, 10, 3, 1, 0.3, 0.1 |
| OH-CLQ | hERG : 30, 10, 3, 1, 0.1  Na_V_1.5, Ca_V_1.2: 1000, 300, 100, 30, 10  Kir2.1, HCN4, K_V_4.3 : 300, 100, 30, 10, 3 | All assays :  300, 100, 30, 10, 3, 1, 0.3, 0.1 |
| AZI | All assays : 1000, 300, 100, 30, 10 | All assays :  1000, 300, 100, 30, 10, 3, 1, 0.3 |
| ERT | All assays : 1000, 300, 100, 30, 10 | All assays :  1000, 300, 100, 30, 10, 3, 1, 0.3 |

***Human in Silico Drug Trials***

The three computational models used to produce our *in silico* results are available online: ToR-Ord ^2^ (https://github.com/elifesciences-publications/torord), ORd-CiPA^3^ (https://github.com/FDA/CiPA), and Ord^4^ (<https://rudylab.wustl.edu/code-downloads/>). Full details about the populations of models used in this study are included in Table S1, to ensure reproducibility of the *in silico* results. These populations were constructed by varying the main ionic current conductances of the three human ventricular action potential (AP) models considered (ToR-ORd, ORd-CiPA, and ORd): fast and late Na^+^ current (I_Na_ and I_NaL_), transient outward K^+^ current (I_to_), rapid and slow delayed rectifier K^+^ current (I_Kr_ and I_Ks_), inward rectifier K^+^ current (I_K1_), Na^+^–Ca^2+^ exchanger (I_NCX_), Na^+^–K^+^ pump (I_NaK_), and L‐type Ca^2+^ current (I_CaL_).

The ranges of variation of these parameters with respect to their baseline values were different for the control (‘healthy’) and high-risk populations. In the control populations, all conductances were varied from 30% to 200% of their baseline values, and a larger number of cells were created (1,500). In the high-risk populations, ranges were designed to maximise the likelihood of cell displaying drug-induced repolarisation abnormalities (RA), similar to what previously done in ^5^ (I_Na_ and I_K1_, 50-200%; I_CaL_, I_NaL_ and I_NCX_, 100-200%; I_to_, 0-200%; I_Kr_ and I_NaK_, 50-100%; I_Ks_, 0-100%), and a smaller number of cells were created (200). ﻿Each initial population was paced at 1 Hz for 500 beats, and the last beat was used to compute the set of AP and Ca^2+^ transient (CT) biomarkers listed in the main text. Only the models with AP and CT biomarkers within physiological ranges – based on experimental recordings from human ventricular myocytes, as in ^5^ – were kept in the final experimentally-calibrated populations. Since all models are randomly generated, each experimentally-calibrated population has a slightly different size: ToR_ORd (394 and 72), ORd-CiPA (406 and 92) and ORd (393 and 118), control and high-risk, respectively.

﻿Following drug application, cell models were paced at 1 Hz for additional 150 beats, and the last simulated AP and CT were considered. All AP traces were automatically checked for repolarisation and depolarisation abnormalities (RA and DA, respectively), as in ^6^. For models not displaying abnormalities, all AP and CT biomarkers were computed, and compared with the control conditions.

**References**

1. Wible, B.A., et al. An ion channel library for drug discovery and safety screening on automated platforms. *Assay Drug Dev. Technol.* 6, 765–780 (2008).
2. Tomek, J., et al. Development, calibration, and validation of a novel human ventricular myocyte model in health, disease, and drug block. *ELife* 8, 1–47 (2019).
3. Dutta, S., et al. Optimization of an in silico cardiac cell model for proarrhythmia risk assessment. *Front. Physiol.* 8, 616 (2017). doi: 10.3389/fphys.2017.00616.
4. O’Hara, T., et al. Simulation of the undiseased human cardiac ventricular action potential: Model formulation and experimental validation. *PLoS Comput. Biol.* *7*(5), e1002061 (2011).
5. Passini, E., et al. Drug-induced shortening of the electromechanical window is an effective biomarker for in silico prediction of clinical risk of arrhythmias. *Br. J. Pharmacol.* 176(19), 3819–3833 (2019). https://doi.org/10.3389/fphys.2017.00668.
6. Passini, E., et al. Human in silico drug trials demonstrate higher accuracy than animal models in predicting clinical pro-arrhythmic cardiotoxicity. *Front. Physiol.* 8, 1–15 (2017). https://doi.org/10.3389/fphys.2017.00668.
