# Supplementary material for: Applying the CiPA approach to evaluate cardiac proarrhythmia risk of some antimalarials used off‐label in the first wave of COVID‐19
Source: Clin Transl Sci. 2021 Apr 9;14(3):1133–46. doi: 10.1111/cts.13011 (PMC8014548; doi:10.1111/cts.13011)
Supplement: Supplementary file 2 — Table S1 [file CTS-14-1133-s002.pdf]

Table S1. Ionic conductances for the control and high-risk populations of human ventricular models used in this study. The values shown (between 0 and 2) represent the scaling factors of all models in the experimentally-calibrated populations, compared to their baseline values.

$\mathbf{G_{Na}}$: fast Na^+ current; $\mathbf{G_{NaL}}$: late Na^+ current; $\mathbf{G_{to}}$: transient outward K^+ current; $\mathbf{G_{Kr}}$: rapid delayed rectifier K^+ current; $\mathbf{G_{Ks}}$: slow delayed rectifier K^+ current; $\mathbf{G_{K1}}$: inward rectified K^+ current; $\mathbf{G_{NCX}}$: Na^+-Ca^{2+} exchanger; $\mathbf{G_{NaK}}$: Na^+-K^+ pump; $\mathbf{G_{CaL}}$: L-type Ca^{2+} current.

ToR-ORd - Control Population - 394 Human Ventricular Cell Models

G_{Na}	G_{NaL}	G_{to}	G_{Kr}	G_{Ks}	G_{K1}	G_{NCX}	G_{NaK}	G_{CaL}
1,80	0,51	0,39	0,50	1,26	0,67	1,33	2,00	1,39
1,11	1,87	1,16	1,68	1,41	1,57	0,80	1,39	1,21
0,76	0,61	0,83	1,90	0,77	0,56	1,50	1,55	1,75
1,13	0,85	1,83	1,31	0,31	1,50	1,88	1,84	1,49
0,83	0,61	1,75	1,26	0,63	1,57	1,55	1,18	1,72
1,05	0,50	1,39	0,67	0,49	1,32	1,03	1,60	1,42
1,87	1,28	1,42	1,18	0,60	1,95	1,07	1,82	1,20
0,35	0,73	1,77	0,51	1,67	0,68	1,77	0,31	0,92
1,79	0,39	1,79	1,18	1,22	1,63	1,68	0,91	1,55
1,89	1,13	0,95	0,84	1,47	0,41	1,52	1,45	1,19
1,90	1,52	0,64	1,26	0,63	1,52	1,63	0,42	1,29
0,76	1,52	1,98	1,65	1,89	0,80	1,73	0,43	1,45
1,63	1,33	1,64	1,16	0,33	1,03	1,18	0,54	1,40
1,74	1,71	1,76	1,38	1,84	1,64	1,49	0,65	0,90
1,90	0,48	1,66	1,09	0,66	0,43	1,50	1,12	1,55
1,18	1,58	0,68	1,81	1,42	0,77	0,67	1,16	1,03
0,91	1,94	1,67	1,42	0,91	0,65	1,45	1,27	1,04
1,32	1,37	0,54	0,85	0,70	0,55	1,59	0,32	1,14
1,93	1,82	1,93	1,70	1,41	1,98	1,04	1,65	1,25
1,68	1,29	1,88	1,92	0,38	1,09	0,94	1,50	1,53
1,48	1,81	1,44	0,93	0,57	1,03	1,23	0,48	0,76
0,41	0,55	1,63	1,28	1,91	0,88	0,93	0,45	0,87
1,32	1,26	1,34	1,06	0,54	2,00	1,00	1,40	1,02
0,35	0,61	1,13	1,63	1,28	0,55	1,48	0,49	1,35
1,51	1,98	1,83	1,30	0,31	0,55	0,73	2,00	1,08
1,23	1,01	0,40	1,17	0,49	1,96	1,40	0,51	1,64
1,11	1,01	1,67	0,66	1,90	1,23	1,12	0,53	0,93
1,27	1,84	0,59	1,12	1,81	1,93	1,90	1,59	1,52
1,45	1,77	0,67	1,63	1,99	0,85	1,34	0,74	1,05
1,67	1,61	1,83	1,15	1,73	0,39	1,73	1,40	1,35
1,40	0,55	1,37	1,15	0,76	1,06	1,63	1,03	1,16
1,22	1,00	0,86	0,88	1,18	1,83	1,44	0,85	1,15
0,67	0,54	0,94	1,54	0,86	1,05	1,63	1,21	1,87
1,87	1,41	1,43	1,04	1,68	1,91	1,04	1,86	1,08
1,81	0,83	1,21	0,77	0,66	1,36	1,22	1,28	1,28
1,48	1,54	0,70	0,90	1,55	1,25	1,76	1,35	1,30
0,87	1,83	1,99	0,97	0,85	1,24	1,81	1,11	1,46
1,04	1,85	1,70	1,87	0,93	1,47	1,51	1,13	1,54
0,41	1,91	1,01	1,18	1,42	1,16	1,50	0,34	1,05
1,74	0,77	1,81	0,82	0,47	1,48	1,97	1,79	1,47
1,54	0,54	1,24	0,73	1,97	1,67	0,72	1,96	1,12
0,92	1,29	0,69	1,13	0,84	1,97	1,08	1,12	1,38
1,13	1,75	1,99	1,74	1,19	1,90	1,79	0,69	1,41
0,88	1,31	1,42	1,22	1,41	1,16	1,13	1,38	1,08

1,53	0,82	0,36	0,63	1,17	1,55	1,55	1,96	1,58
1,58	1,93	1,26	1,86	1,22	1,57	0,80	0,49	0,95
1,36	0,66	1,05	0,61	1,23	1,47	1,46	0,53	1,14
1,29	0,93	0,79	0,81	2,00	0,51	1,89	1,56	1,42
1,10	1,06	0,34	0,99	0,62	0,63	1,03	0,74	1,11
1,30	1,12	0,67	1,61	1,28	1,55	0,74	1,64	1,24
0,86	0,91	1,38	1,26	1,52	1,93	1,35	0,78	1,59
0,30	0,64	1,38	1,35	1,17	0,34	1,61	1,42	1,69
0,97	1,47	0,64	1,32	1,35	1,68	1,64	1,63	1,96
0,56	1,05	1,37	1,03	1,62	0,89	1,85	1,68	1,75
0,63	0,57	0,45	0,42	1,19	1,99	1,14	0,83	1,38
1,01	1,30	1,71	0,85	0,70	1,11	0,34	1,17	0,57
1,49	0,71	1,54	1,39	0,55	0,44	0,55	1,73	0,98
0,63	1,14	0,93	1,40	0,39	1,17	0,46	0,86	0,71
1,25	1,73	1,66	0,83	0,90	1,59	1,38	1,87	1,27
1,50	1,42	1,12	1,39	1,99	1,14	1,24	1,95	1,50
1,37	1,79	1,17	2,00	1,64	0,98	0,44	1,73	0,48
1,99	1,73	1,38	1,45	1,20	0,88	0,62	0,45	0,60
0,76	1,07	1,29	0,58	0,95	1,69	0,76	0,52	0,77
0,67	1,19	0,72	1,62	0,37	1,62	1,78	0,58	1,70
1,47	1,63	1,47	1,22	0,46	0,41	0,39	1,71	0,54
1,77	1,63	1,77	1,16	1,55	1,83	0,82	1,40	1,12
1,51	1,51	0,61	1,03	1,64	0,95	1,91	1,51	1,80
1,57	0,68	0,87	1,30	1,64	1,82	1,40	1,13	1,82
1,76	0,76	0,90	0,67	0,62	1,95	1,27	0,81	0,92
1,01	0,42	0,75	0,62	0,31	1,70	1,26	0,50	0,99
0,64	0,71	0,70	0,42	1,74	1,33	1,21	1,08	0,88
1,59	1,82	1,68	1,69	0,74	0,62	1,28	1,29	1,03
1,53	1,71	1,13	1,46	1,75	1,77	1,61	0,43	1,00
1,34	1,60	1,94	1,87	0,38	1,32	0,51	1,07	0,64
0,61	0,94	0,38	1,01	1,26	0,84	1,36	0,82	0,92
1,85	0,65	0,59	1,24	1,20	1,32	1,60	0,87	1,85
1,17	1,53	1,24	1,49	1,12	0,62	1,51	0,71	0,96
1,76	1,21	1,15	0,61	1,80	0,68	0,70	1,90	1,20
0,96	1,66	0,55	1,58	0,94	1,75	1,87	0,95	1,31
1,69	0,30	1,72	0,89	0,34	1,30	0,78	1,65	1,18
0,83	1,82	1,59	1,91	0,97	0,58	0,71	1,87	0,94
2,00	1,89	1,25	1,13	1,87	0,96	1,80	1,48	1,48
1,35	1,13	0,46	1,09	0,77	0,44	1,00	1,23	1,35
0,92	1,19	0,39	0,42	1,69	1,40	0,90	1,59	1,05
0,71	1,96	1,89	1,36	1,80	0,59	1,49	0,92	1,39
1,55	1,60	1,18	1,61	1,62	1,86	1,23	1,44	1,66
1,53	1,64	0,31	1,23	1,47	0,76	1,55	1,09	1,21
0,62	1,36	1,52	0,77	1,65	1,81	0,56	0,69	0,48
1,76	0,48	1,78	0,89	0,46	0,51	1,05	0,64	1,31
0,62	0,70	1,18	0,53	0,94	1,16	1,53	1,86	1,44
1,43	0,40	1,93	0,62	1,05	0,97	1,84	1,49	1,47

0,37	1,17	1,97	0,76	0,72	0,35	0,89	1,87	0,94
1,14	0,74	1,56	1,39	1,99	1,99	1,52	0,72	1,45
1,93	0,94	0,43	1,41	1,53	0,56	1,70	0,37	1,51
1,24	1,88	1,20	1,10	1,13	1,52	0,94	1,33	1,12
1,62	0,68	1,70	1,52	0,96	1,69	1,44	0,92	1,40
1,76	1,72	0,91	1,23	1,54	1,21	0,50	1,70	0,51
1,70	1,57	0,92	1,63	0,41	1,66	1,43	1,10	1,52
1,82	1,45	0,32	0,99	0,42	0,43	0,60	1,74	0,74
0,92	1,27	0,44	1,19	0,61	0,47	0,31	1,27	0,48
0,80	0,41	0,69	1,37	1,44	1,18	1,69	1,90	1,68
1,08	0,98	1,65	1,10	1,81	1,40	1,42	0,58	1,00
1,71	1,66	1,26	1,64	0,72	1,49	1,20	1,12	1,63
1,62	1,71	0,98	1,26	1,52	1,61	1,56	1,67	1,62
1,51	1,74	1,68	1,74	1,17	1,43	1,77	0,62	1,02
0,60	1,23	1,29	1,16	1,88	1,81	1,16	0,42	1,02
0,89	0,94	1,88	1,21	1,81	0,55	1,32	0,89	1,49
0,73	1,57	1,12	0,94	0,42	0,75	1,87	1,33	1,60
1,94	1,67	1,97	0,89	1,29	0,62	0,80	1,12	0,66
1,31	0,72	1,56	1,31	0,68	0,38	1,97	1,15	1,49
0,58	0,60	0,86	0,90	1,44	0,96	1,37	1,11	1,00
1,18	0,44	1,13	0,43	0,44	1,63	1,01	0,76	1,16
1,64	1,53	0,39	1,18	0,52	1,64	1,70	1,86	1,82
1,18	0,63	1,06	1,79	1,16	0,56	0,85	1,76	1,50
1,20	1,51	1,00	0,32	1,11	1,54	0,75	0,84	0,70
1,11	1,67	1,52	1,02	0,48	0,87	0,49	1,74	0,62
1,33	1,71	1,72	1,75	2,00	0,36	1,07	1,58	1,17
1,65	0,65	1,19	1,45	1,80	1,04	1,67	0,46	1,63
0,38	0,79	1,64	0,88	1,07	0,69	1,96	1,02	1,60
1,81	1,96	1,83	0,81	0,83	1,30	1,65	1,18	1,66
1,92	1,40	1,71	0,78	0,70	1,13	1,10	1,23	0,89
0,66	1,27	1,38	1,92	0,51	0,94	1,88	0,33	1,53
1,13	1,57	0,79	1,16	0,36	1,53	1,27	1,81	1,79
0,58	0,71	1,56	1,61	1,93	0,37	1,39	0,56	1,51
0,83	1,07	1,06	0,63	0,72	0,85	1,11	1,17	1,06
0,56	1,87	0,84	0,94	1,10	0,85	0,41	1,05	0,43
1,96	1,26	1,74	1,57	0,92	0,45	1,00	1,91	1,61
0,79	1,61	1,81	0,94	0,73	1,51	1,65	0,34	1,08
1,61	1,38	0,79	0,70	0,43	1,34	1,46	0,79	1,38
1,39	1,43	0,96	0,76	1,42	0,79	1,36	1,32	1,05
1,70	1,88	0,60	1,78	1,27	1,47	0,99	1,68	1,65
1,90	1,03	1,91	1,72	0,60	0,41	1,28	1,67	1,24
1,15	0,33	0,35	0,93	0,52	1,12	1,51	0,72	1,44
1,75	0,84	1,51	0,90	1,79	0,33	1,16	0,65	1,17
1,52	1,11	1,03	1,80	1,94	1,60	1,18	0,40	0,97
1,54	0,55	1,25	0,43	1,73	1,80	0,64	0,40	0,88
0,33	0,82	0,62	1,57	0,65	0,39	1,88	1,72	1,49
1,25	0,67	1,75	0,47	1,99	1,02	0,96	1,59	1,33

0,43	0,96	1,60	1,06	1,94	1,74	1,67	1,91	1,97
1,75	0,80	0,73	0,89	1,12	0,41	0,91	1,85	1,11
1,80	0,63	0,93	0,77	1,36	0,47	1,95	1,44	1,81
1,50	1,83	0,73	1,04	1,52	1,10	1,54	2,00	1,96
0,82	1,82	1,06	1,08	1,12	0,35	0,97	0,59	0,95
0,59	0,62	1,94	1,23	1,23	0,77	1,36	1,41	1,16
1,87	0,75	0,85	1,41	1,78	0,73	0,97	0,81	1,34
1,54	0,39	1,62	1,28	0,82	0,31	0,88	1,34	1,18
1,71	1,11	1,30	1,59	0,35	0,96	1,40	1,10	1,37
1,59	0,94	1,84	0,51	1,98	0,52	1,13	1,13	1,01
1,11	0,65	1,95	0,34	1,68	1,56	0,85	1,04	1,06
1,49	1,54	0,75	1,71	0,68	0,86	0,71	1,43	0,76
0,52	0,75	1,12	0,82	0,69	1,42	1,25	0,86	1,37
1,28	1,12	0,50	1,09	0,97	1,77	0,85	1,50	1,15
0,82	1,29	1,56	1,14	1,32	1,14	0,43	1,69	0,46
0,44	1,23	0,45	1,40	1,43	1,15	1,27	1,03	1,33
1,50	0,65	1,92	1,46	1,90	0,38	1,01	1,04	1,34
1,67	0,79	1,97	0,54	1,03	1,40	1,40	1,29	1,68
1,14	0,56	1,90	0,62	1,80	1,87	1,05	1,37	1,08
1,99	1,69	0,78	1,00	0,50	0,86	1,98	1,24	1,59
0,83	1,30	1,26	0,69	0,66	1,23	1,66	1,11	1,00
0,33	0,60	1,00	0,35	1,21	0,33	0,64	1,64	0,86
1,00	1,42	1,83	1,07	1,53	1,03	1,53	1,17	1,35
1,07	0,89	1,88	1,54	0,53	1,71	1,62	1,30	1,79
1,47	1,43	0,83	0,45	1,90	1,39	0,65	1,98	1,07
1,93	1,16	1,67	1,21	1,02	1,42	1,97	0,61	1,30
0,52	1,85	1,39	1,36	0,97	0,88	1,33	1,00	1,44
0,81	1,11	0,41	1,73	0,81	0,52	1,69	1,23	1,30
1,96	1,65	0,83	1,44	1,40	0,98	1,86	1,43	1,84
1,34	0,68	1,94	0,82	1,90	1,56	1,33	1,01	1,20
1,57	0,64	1,73	1,13	1,73	0,62	1,37	1,29	1,69
0,64	0,38	1,27	1,31	1,03	0,92	1,79	0,82	1,54
1,49	1,53	0,60	1,29	0,86	1,65	1,72	1,16	1,74
1,23	1,44	1,05	1,49	1,02	1,08	1,96	1,52	1,62
0,84	0,83	1,82	1,24	1,92	0,66	1,00	1,84	1,15
1,47	1,39	0,79	1,42	1,52	1,61	1,76	0,49	1,09
1,44	1,40	1,41	0,93	1,47	0,53	0,56	0,83	0,66
0,78	1,95	1,75	1,05	0,32	1,79	1,34	1,42	0,84
1,49	0,96	1,09	1,96	1,70	0,65	1,87	0,53	1,22
1,24	1,26	0,83	1,34	1,91	0,89	1,57	1,33	1,92
0,54	1,55	0,45	1,30	1,66	0,57	1,11	0,99	1,09
0,89	1,34	1,27	1,23	1,10	0,36	1,71	0,84	1,12
0,60	1,75	0,30	1,09	0,68	1,78	1,61	1,63	1,35
1,38	1,74	0,53	1,71	1,19	1,38	0,63	1,18	1,10
1,74	1,98	1,09	1,23	0,56	0,79	1,06	0,68	0,89
1,76	0,60	1,64	0,70	1,32	0,34	1,50	1,61	1,78
1,37	1,60	1,38	1,02	0,47	1,06	1,97	2,00	1,88

1,19	0,63	0,47	0,89	1,72	0,86	0,56	0,73	0,70
0,91	1,93	0,73	1,97	1,44	0,42	0,81	0,50	0,95
0,55	0,56	1,68	1,05	1,93	0,84	0,72	1,61	1,03
1,25	0,94	0,33	1,19	0,62	1,97	0,61	1,03	0,82
0,72	0,40	0,91	0,48	1,44	1,93	1,54	0,97	1,14
1,53	0,76	0,52	0,78	1,99	0,58	1,71	0,57	1,57
0,51	0,34	0,49	1,68	1,47	0,85	1,81	1,09	1,83
0,91	0,50	1,09	1,07	0,44	0,71	1,77	1,95	1,90
1,23	1,44	0,66	1,80	1,19	0,91	0,39	1,74	0,72
1,49	0,71	1,52	0,96	1,94	1,93	1,83	1,10	1,96
0,69	0,81	0,55	1,36	1,14	0,58	1,57	1,14	1,60
1,84	1,18	0,67	1,29	1,34	0,40	0,45	1,97	1,02
1,94	1,24	1,61	1,75	1,15	0,83	1,15	0,99	0,99
1,03	1,01	0,54	1,35	0,60	1,91	1,52	1,93	1,94
1,22	1,79	0,87	1,24	1,36	0,53	1,36	0,54	1,48
1,08	0,47	1,55	1,54	1,81	0,55	1,00	0,97	0,98
1,92	0,90	1,05	1,03	1,01	1,67	1,57	1,90	1,82
1,57	1,79	1,00	0,94	1,18	0,66	1,87	0,77	1,42
0,35	1,08	0,51	1,52	1,28	0,34	1,24	1,51	1,65
0,65	1,34	0,99	1,56	0,99	0,40	0,99	1,21	1,30
1,85	1,47	0,67	1,41	1,85	0,79	1,42	0,42	1,36
1,86	1,73	0,42	1,24	1,14	0,50	1,45	0,91	1,31
1,32	1,72	0,59	1,59	1,68	1,89	1,77	1,36	1,81
1,65	1,00	0,93	1,67	0,42	1,77	1,29	1,82	1,26
1,64	0,94	1,80	1,04	1,30	1,66	1,09	1,20	1,26
1,36	0,52	1,39	0,93	1,36	1,99	1,87	0,76	1,55
1,21	1,76	1,89	1,52	0,58	1,45	0,98	1,92	1,36
0,46	0,67	0,33	0,97	0,67	0,69	0,39	1,32	0,62
0,97	1,73	0,66	1,76	1,82	0,40	0,70	1,64	1,18
1,62	0,91	1,33	1,34	1,85	0,46	1,32	1,77	1,74
1,94	1,48	1,90	1,67	1,74	1,35	1,03	0,67	1,32
1,09	0,99	0,66	1,97	1,77	0,65	1,74	0,68	1,54
1,30	1,71	1,23	0,72	1,23	0,79	1,37	1,25	1,18
1,18	1,25	1,08	0,87	1,50	0,38	0,53	1,61	0,90
1,08	0,66	1,91	0,43	0,78	0,74	1,14	0,46	0,85
1,45	0,69	0,32	1,11	0,64	1,41	1,71	1,61	1,94
0,46	0,72	0,81	0,48	0,54	0,77	0,76	0,87	0,85
0,39	0,76	1,04	0,76	1,86	1,68	1,06	1,37	1,13
0,49	1,68	1,45	1,84	1,01	0,33	0,77	0,39	0,81
1,29	1,22	1,96	0,92	0,57	1,62	1,14	1,89	1,47
2,00	0,77	1,95	0,38	1,65	1,09	1,29	1,01	0,95
1,42	0,31	0,96	0,83	1,25	1,50	1,78	1,22	1,52
1,34	0,82	0,86	0,59	0,78	1,97	0,46	1,28	0,79
1,30	0,62	1,50	1,86	0,58	1,04	1,14	0,43	1,46
1,02	1,37	0,60	1,56	1,48	0,64	1,40	1,27	1,25
1,72	1,74	0,48	1,71	1,25	0,35	0,75	1,30	1,36
1,17	1,12	1,19	0,65	1,93	0,95	0,66	1,06	0,68

1,37	0,68	1,17	1,59	1,60	0,71	1,82	0,33	1,91
1,85	0,67	0,60	1,17	1,42	0,36	1,22	1,68	1,86
1,31	1,81	0,48	1,03	1,31	0,62	1,38	1,68	1,29
0,57	1,26	1,30	1,02	1,94	1,65	0,96	1,31	0,77
0,56	0,32	1,91	0,96	0,78	0,50	0,63	1,99	0,71
1,41	1,00	0,77	0,38	1,81	1,10	0,58	1,47	0,97
0,52	1,06	1,45	0,97	0,99	1,71	0,40	0,80	0,61
1,19	0,59	1,69	0,79	1,49	0,82	1,12	1,53	1,54
1,94	0,85	0,79	1,13	0,99	0,49	1,21	1,29	1,28
1,82	1,36	0,38	1,71	1,63	1,99	0,33	1,39	0,78
1,63	1,81	1,77	0,92	0,62	1,01	1,11	1,77	0,96
1,68	0,86	1,20	1,25	1,53	0,34	0,65	1,67	0,76
1,12	1,01	1,06	1,47	1,43	0,60	1,84	1,35	1,86
1,84	1,35	1,96	1,61	0,67	0,53	1,93	0,73	1,47
1,56	1,78	0,55	1,11	0,65	0,95	1,92	0,58	1,57
1,33	1,42	0,58	1,73	1,41	0,70	1,35	1,40	1,52
0,43	0,45	0,37	1,19	0,37	0,72	0,91	0,36	1,38
0,66	0,38	0,79	1,07	0,33	1,55	1,77	1,53	1,23
1,01	1,53	1,39	0,86	1,04	1,02	1,24	0,42	1,22
1,63	1,50	1,08	1,33	1,82	1,41	0,92	0,47	0,83
1,61	1,70	1,59	0,78	1,50	0,67	0,61	1,84	0,82
0,48	0,84	1,50	0,98	0,43	0,69	1,37	1,48	1,20
1,29	1,56	0,84	1,78	1,82	0,45	1,16	1,89	1,43
0,97	0,36	0,76	0,97	0,41	1,01	1,32	0,82	1,31
1,87	0,70	1,80	0,96	0,70	1,43	1,45	1,07	1,80
1,04	1,78	0,37	1,73	1,25	1,30	1,55	0,99	1,11
1,35	1,58	1,87	0,87	1,31	0,74	1,39	1,51	1,29
1,78	0,69	1,54	0,61	0,51	1,90	1,73	0,53	1,53
0,45	1,14	0,69	1,46	0,76	0,87	0,33	1,66	0,59
0,75	0,44	1,17	0,80	0,50	1,36	0,89	1,44	1,12
1,69	1,67	1,86	0,86	1,60	1,91	1,41	1,78	1,68
1,54	1,44	0,62	1,21	0,75	0,57	0,72	0,64	0,79
1,23	1,95	1,22	1,14	1,76	1,15	1,08	1,94	1,21
0,99	0,74	1,35	1,67	0,35	0,41	1,09	1,63	1,65
1,93	1,69	1,82	1,32	1,60	1,69	1,22	0,97	1,06
1,32	1,66	1,22	1,90	1,34	1,64	1,89	0,39	1,58
1,08	1,07	0,46	1,56	0,94	1,58	1,94	1,26	1,42
1,68	0,81	1,44	1,39	0,35	1,98	1,80	1,06	1,74
1,20	0,82	0,75	1,03	1,46	1,07	0,92	1,04	0,87
1,54	0,32	1,61	0,73	0,95	1,67	1,64	0,41	1,73
1,44	1,79	1,41	1,35	1,24	1,65	1,28	0,86	1,33
0,32	0,55	1,42	0,51	0,64	0,92	0,72	1,33	0,62
0,36	1,09	0,42	0,68	0,36	1,12	1,50	1,41	1,49
1,28	0,87	1,14	1,63	1,03	1,19	1,35	1,21	1,57
1,70	1,27	1,97	1,48	1,98	0,51	1,79	0,77	1,24
1,21	1,59	1,45	0,84	0,42	0,43	1,14	0,47	1,21
0,53	1,62	1,64	1,00	1,31	1,38	1,12	1,42	1,22

1,35	1,68	0,87	1,93	0,75	0,96	1,69	1,83	1,89
1,25	0,44	0,52	0,63	0,37	1,20	1,68	1,35	1,65
1,99	0,88	1,92	0,88	0,89	1,24	1,39	0,99	1,25
0,80	0,89	1,94	0,48	1,84	0,44	0,72	1,98	1,07
1,71	1,51	1,54	1,77	1,35	0,93	0,88	0,87	0,90
0,58	0,69	0,31	1,50	1,34	1,29	1,89	1,24	1,97
1,44	0,82	0,43	1,30	0,42	1,16	1,40	1,77	1,28
1,02	0,34	1,08	1,39	1,36	1,46	1,99	1,02	1,82
1,94	0,53	1,93	1,62	0,76	1,33	1,68	1,95	1,94
0,31	0,44	1,87	1,88	1,46	0,64	1,81	0,50	1,42
1,97	1,99	1,11	1,94	1,94	1,55	1,95	0,46	1,28
1,00	0,46	1,87	1,54	1,98	1,96	1,92	0,53	1,82
1,88	1,33	1,00	1,48	1,70	0,42	1,79	0,91	1,89
1,54	1,63	0,67	1,35	0,69	1,63	1,59	1,86	1,96
1,61	1,63	0,59	1,44	1,80	0,46	0,86	1,26	1,18
0,50	0,38	0,44	0,30	1,63	1,25	0,74	0,36	0,92
0,63	0,68	1,64	0,31	0,39	1,80	1,27	0,48	0,93
1,04	1,95	0,55	1,84	1,10	1,39	1,74	0,98	1,79
1,02	0,77	0,91	1,70	1,95	0,32	1,56	0,93	1,37
1,43	1,18	1,03	1,83	0,64	0,76	0,68	1,47	0,77
0,42	1,11	1,46	0,69	1,22	1,08	0,86	1,17	0,93
0,73	1,32	1,76	1,10	0,58	1,06	2,00	1,19	1,62
1,75	1,74	1,98	1,36	0,74	0,70	0,90	0,87	0,72
0,74	0,87	1,89	1,26	0,71	0,63	1,26	1,23	0,93
1,97	0,89	1,19	0,89	0,65	0,44	1,58	1,57	1,28
1,10	0,59	0,86	0,90	0,93	0,66	1,21	1,23	1,14
1,35	1,09	1,13	1,11	1,04	0,83	0,81	1,19	0,78
1,85	1,27	0,84	1,60	0,84	1,63	0,34	1,96	0,49
0,75	1,49	0,73	1,21	1,00	0,39	1,74	1,16	1,61
0,87	1,92	1,07	1,88	0,64	1,39	1,72	1,08	1,76
1,24	1,26	1,52	0,70	1,37	1,47	1,78	1,60	1,17
1,57	1,42	1,82	1,47	0,94	1,20	1,41	0,74	1,32
1,89	0,80	0,78	0,84	1,13	1,35	1,75	0,48	1,24
1,23	0,41	1,61	1,07	1,09	1,56	1,58	0,41	1,67
0,48	1,55	0,66	1,86	0,89	0,60	1,70	1,14	1,63
1,13	1,42	1,48	1,29	0,75	1,53	1,09	0,83	1,26
0,83	0,65	0,62	0,83	1,02	0,95	1,49	1,47	1,93
1,64	0,91	0,70	0,63	1,80	1,43	1,59	1,97	1,32
1,09	1,16	0,86	1,99	1,71	0,46	1,86	0,63	1,35
1,91	0,32	1,13	1,01	1,04	1,76	1,29	1,24	1,11
1,56	0,84	1,25	0,59	0,64	0,50	1,38	1,44	1,37
1,80	1,65	0,72	1,19	1,04	1,99	1,79	0,38	1,07
1,85	0,46	0,38	1,77	0,50	1,35	1,72	1,43	1,94
1,99	1,08	1,84	0,99	1,15	1,59	1,38	1,58	1,45
1,96	0,98	0,50	1,96	1,54	1,59	1,71	0,80	1,94
1,88	1,97	1,07	1,53	0,75	0,43	1,48	0,57	1,59
1,32	1,67	0,84	1,10	1,13	1,25	1,25	0,68	0,96

1,12	0,92	2,00	1,42	1,85	1,20	1,15	0,45	1,07
0,57	1,93	0,75	1,42	1,43	0,91	1,67	1,72	1,23
1,55	1,82	0,30	1,05	0,97	1,40	1,56	1,72	1,97
1,82	0,62	0,32	1,56	1,61	1,73	1,69	0,95	1,70
1,42	1,67	1,89	1,12	0,60	0,45	0,54	1,96	0,52
0,60	1,58	0,31	1,01	0,87	1,43	1,56	0,48	1,19
0,41	0,81	1,60	1,91	1,89	0,46	1,22	1,46	1,33
1,34	1,13	1,05	1,89	1,58	0,54	1,36	1,05	1,50
0,36	1,23	1,49	1,46	0,57	0,70	0,71	0,39	1,08
0,84	1,06	1,36	1,51	0,88	0,63	0,90	1,90	1,43
0,59	0,46	1,48	1,38	1,18	0,39	1,72	1,21	1,46
0,37	1,86	0,41	1,28	0,68	1,09	0,52	1,64	0,54
1,25	1,02	1,18	1,19	1,09	1,20	1,17	1,55	1,51
1,99	1,21	0,45	0,82	0,36	1,26	1,68	0,64	1,53
0,56	0,78	0,79	1,57	1,63	1,82	1,34	0,31	1,57
1,78	1,94	0,95	1,35	0,52	0,98	0,51	1,00	0,78
1,67	0,86	1,48	0,50	0,38	1,07	1,15	1,98	1,02
0,54	1,28	1,29	1,20	0,54	0,51	1,83	0,66	1,27
1,17	1,78	0,98	1,41	1,96	0,44	0,70	1,57	1,13
0,34	0,75	1,14	1,29	0,82	0,69	1,27	0,89	1,33
1,71	0,66	1,10	1,11	1,01	0,81	1,83	0,61	1,32
1,15	1,00	0,74	1,25	0,37	1,43	1,19	0,95	1,31
1,82	1,16	0,33	0,91	1,96	1,61	0,81	1,38	1,34
0,78	1,09	1,36	1,46	1,12	0,84	1,47	1,64	1,76
0,95	1,60	0,91	0,96	0,75	0,62	0,93	0,61	1,13
0,55	1,16	1,66	1,41	1,75	0,65	1,43	1,25	1,63
1,95	1,72	1,46	1,11	0,87	1,95	1,89	0,61	1,56
0,93	1,97	1,20	1,49	1,76	0,77	1,61	1,05	1,39
1,71	1,04	1,02	1,09	1,00	1,76	1,25	1,24	1,32
1,60	1,58	0,48	1,63	0,50	1,58	1,80	1,22	1,77
1,65	1,47	0,91	1,98	1,85	0,61	1,46	1,86	1,61
1,88	1,92	0,37	1,21	1,68	1,19	0,92	0,44	1,06
1,89	1,12	1,67	1,54	1,45	1,50	1,27	0,66	1,52
1,27	0,35	0,71	0,70	0,72	1,22	1,10	0,79	1,59
2,00	0,40	0,56	1,61	0,79	1,20	0,88	1,20	1,03
1,04	1,25	1,54	0,51	0,79	0,74	0,40	0,65	0,39
1,75	0,51	1,58	0,75	1,76	1,09	1,48	1,44	1,27
0,96	1,33	0,96	1,99	1,39	0,39	1,47	1,54	1,69
1,86	1,49	0,98	1,33	1,72	1,62	1,64	0,91	1,73
0,51	1,11	0,71	1,26	1,68	1,06	1,86	0,71	1,34
0,95	0,61	1,42	1,37	1,07	1,69	1,97	1,45	1,67
1,82	1,45	1,45	0,71	1,64	0,86	0,96	1,95	1,18
1,04	0,78	1,01	1,29	1,36	1,02	1,85	1,31	1,25
1,09	1,69	1,77	0,88	1,74	0,49	1,88	1,41	1,05
1,70	0,60	1,80	0,64	0,85	1,64	1,16	1,25	1,07
0,68	0,52	1,53	1,08	1,60	0,56	1,25	1,33	1,62
0,55	1,75	0,96	1,44	0,74	0,85	1,77	1,85	1,83

1,78	0,88	1,16	0,98	1,46	0,93	1,13	0,81	1,13
0,95	0,36	0,39	0,36	1,23	1,93	1,46	1,91	1,38
1,95	1,15	1,11	0,98	0,43	0,74	1,73	1,17	1,31
1,97	1,85	1,14	1,41	1,03	0,52	1,42	1,10	1,45
1,89	1,25	0,81	1,36	0,61	0,80	1,38	0,46	0,96
1,83	1,36	1,51	0,64	1,62	1,22	0,89	0,33	0,69
1,01	1,83	1,08	1,31	1,39	1,87	1,83	1,87	1,69
1,84	0,43	1,51	1,12	0,40	0,58	1,08	1,52	1,78
1,62	1,20	1,84	1,75	1,77	0,70	1,08	1,23	1,38
1,12	0,31	0,85	0,74	1,71	1,73	1,64	1,69	1,89
0,41	1,79	2,00	0,45	1,00	0,75	0,52	0,95	0,53
1,42	1,68	0,89	1,47	1,59	1,93	1,09	1,18	1,16
0,30	1,82	1,04	1,36	1,43	0,71	1,17	1,30	0,89
1,98	1,98	1,22	1,74	1,25	1,24	1,99	1,34	1,41
0,82	1,80	1,12	1,24	1,75	0,60	1,49	1,97	1,75
1,47	0,74	1,50	1,55	1,25	0,88	1,55	0,53	1,60
1,20	1,21	1,90	1,26	1,29	0,37	1,85	0,76	1,09
0,74	1,49	0,73	1,20	0,64	1,51	0,81	1,08	1,01
0,82	1,21	1,36	0,54	1,21	1,17	0,45	1,28	0,50
0,38	1,54	1,25	1,13	0,85	1,05	1,51	1,66	1,30
1,87	1,00	1,14	0,69	1,07	1,52	0,67	0,93	0,82

ORd2-CiPA - Control Population - 406 Human Ventricular Cell Models

G_{Na}	G_{NaL}	G_{to}	G_{Kr}	G_{Ks}	G_{K1}	G_{NCX}	G_{NaK}	G_{CaL}
1,88	0,96	1,28	0,74	1,92	0,86	0,30	1,74	0,48
1,89	1,65	0,61	1,10	1,72	0,66	0,64	1,62	0,84
1,32	1,26	1,61	0,78	0,70	0,77	1,53	0,55	0,85
0,98	1,09	0,69	0,49	1,83	0,43	1,36	1,80	1,65
1,38	0,67	0,65	1,09	1,18	0,73	1,22	0,47	1,18
0,63	1,58	1,57	1,27	1,02	1,10	1,43	1,92	1,34
0,96	0,44	1,41	0,43	0,33	1,13	1,01	0,38	0,89
1,68	1,70	1,49	1,42	1,18	1,60	1,39	1,45	1,76
1,13	1,17	0,50	0,99	0,60	0,41	1,81	0,57	1,86
1,67	0,70	1,09	0,86	0,50	1,04	1,50	0,67	1,47
0,92	1,65	1,36	1,58	0,64	0,96	1,60	1,76	1,47
1,11	0,87	1,53	1,59	0,53	0,74	1,67	1,96	1,68
1,88	1,83	1,20	1,60	1,17	1,21	1,87	0,69	1,97
0,63	0,56	0,62	0,56	1,33	1,94	1,84	1,37	1,54
0,73	0,87	0,60	1,45	0,90	0,87	1,97	0,60	1,69
0,89	1,07	1,13	0,82	1,30	0,78	0,70	1,62	1,33
1,17	1,42	0,31	1,29	1,16	0,67	0,60	1,59	0,78
1,49	1,14	1,44	1,24	1,53	1,79	1,45	1,04	1,56
0,47	0,36	1,42	0,79	1,32	0,86	0,83	1,49	0,84
1,51	1,19	0,53	0,83	1,68	1,09	1,52	1,40	1,53
1,69	0,41	0,60	1,11	0,57	1,32	1,12	1,16	1,21
0,99	1,52	1,53	1,17	1,52	0,58	1,45	1,06	1,54
1,53	1,86	1,70	0,88	1,21	1,88	1,46	0,55	1,23
0,38	0,40	1,29	1,37	0,72	0,85	0,69	1,89	1,08
0,47	0,73	1,38	1,20	1,63	0,46	1,26	0,90	1,72
0,95	0,54	1,68	1,26	1,15	0,62	0,72	1,13	1,06
1,89	0,39	1,87	0,73	0,31	0,60	1,32	1,98	1,43
1,99	1,24	1,08	0,79	1,31	0,33	1,14	0,35	0,87
1,92	1,38	0,82	1,82	1,56	0,38	1,68	1,39	1,85
0,35	1,77	0,66	0,64	1,85	0,58	0,93	1,63	1,02
1,67	1,70	1,33	1,21	0,56	0,96	0,71	0,84	0,97
1,24	0,39	0,82	0,75	0,94	0,40	1,50	0,93	1,18
1,42	0,48	1,56	1,21	1,48	0,62	0,74	1,19	1,51
0,54	2,00	1,55	1,36	1,13	1,00	0,78	1,37	1,32
1,23	1,65	0,54	0,96	0,58	0,94	0,34	1,37	0,44
1,55	1,09	1,69	1,26	1,30	1,50	1,95	0,81	1,95
0,38	0,78	0,61	1,04	1,94	0,41	0,50	0,80	0,69
1,70	1,40	1,40	1,03	0,35	1,31	1,34	0,71	0,80
1,27	0,46	1,14	0,70	0,59	0,89	1,41	0,73	0,97
0,62	0,87	0,84	0,81	0,31	1,20	0,87	0,42	0,78
0,82	0,66	0,96	1,19	1,61	0,99	1,57	1,53	1,80
1,35	1,10	0,48	1,23	1,34	0,50	1,18	1,83	1,88
0,73	1,30	1,14	1,32	1,75	1,48	0,83	1,31	0,86
1,95	1,39	1,36	0,67	0,85	1,36	0,60	1,40	0,63

1,80	1,52	0,68	1,07	1,05	1,64	0,60	0,43	0,42
0,80	1,23	0,34	1,26	1,46	1,26	0,63	0,43	0,82
1,26	0,41	0,98	0,66	0,76	0,57	0,60	1,49	0,76
1,66	1,23	0,39	0,31	1,80	1,57	1,18	1,32	1,22
0,54	0,82	1,73	1,60	1,94	0,31	0,88	0,94	1,57
0,36	0,48	0,87	1,41	0,35	0,52	1,21	0,55	1,26
0,65	1,36	1,35	1,22	0,78	1,89	1,32	1,27	1,64
1,95	1,95	1,73	1,44	0,52	0,91	1,82	0,72	1,84
0,38	0,97	1,28	1,12	1,26	0,94	1,53	0,37	0,93
0,61	1,21	1,23	1,36	1,69	1,36	0,86	1,36	1,31
1,08	0,30	1,80	0,90	0,80	0,92	1,73	1,00	1,77
1,35	2,00	1,50	1,28	1,85	0,61	0,44	0,89	0,67
0,88	1,34	1,15	0,86	0,54	1,58	1,87	1,54	1,65
0,30	1,11	1,95	1,28	0,80	0,51	1,05	0,62	1,40
1,53	0,77	0,41	1,20	1,78	0,63	1,16	0,82	1,57
1,83	1,42	1,26	1,30	0,45	1,88	1,62	1,00	1,45
0,53	1,07	0,94	0,56	1,22	0,48	0,86	0,81	0,66
0,92	1,73	1,67	1,51	1,68	0,32	0,83	0,81	0,99
1,43	1,04	1,32	0,65	1,43	0,77	1,44	1,73	1,36
1,36	0,71	1,11	0,48	1,53	1,74	1,88	0,78	1,70
1,46	0,36	1,91	1,02	0,83	0,88	0,86	1,55	1,67
0,88	1,36	1,30	1,02	1,12	1,05	0,88	1,72	1,55
1,75	0,80	1,41	0,36	1,93	1,31	1,93	1,69	1,59
0,97	1,91	1,35	1,67	0,38	0,92	0,46	1,36	0,88
0,31	1,14	1,68	0,35	2,00	1,11	1,84	0,54	1,48
0,74	0,50	0,44	1,19	1,09	0,93	1,24	0,76	1,71
1,95	0,70	1,63	1,19	1,86	0,74	0,40	1,09	0,61
0,54	1,80	0,82	1,71	0,53	0,36	1,18	0,75	1,83
0,65	1,46	1,77	1,51	0,46	0,42	1,23	0,78	1,13
1,32	1,33	1,93	0,68	1,47	1,47	1,36	1,29	1,91
1,47	0,78	1,45	0,63	1,75	0,36	1,43	1,44	1,29
0,57	0,48	1,42	0,44	1,73	1,43	0,92	1,73	0,95
1,41	0,61	0,53	1,24	1,37	0,65	0,95	1,29	1,45
0,63	0,46	1,22	0,60	1,10	1,46	1,23	0,60	0,96
1,10	0,91	0,77	1,47	1,97	0,84	0,92	0,91	1,60
0,31	0,94	1,38	1,20	0,34	0,97	1,35	0,99	1,87
1,67	1,21	1,23	1,73	1,16	0,45	1,75	0,99	1,94
1,48	0,37	1,86	0,69	1,78	1,84	1,55	1,80	1,97
1,96	1,96	0,78	0,58	1,02	1,78	0,58	0,51	0,57
0,53	1,74	1,71	1,77	1,70	0,43	0,85	1,02	1,38
1,81	1,05	1,53	0,96	0,96	1,26	1,47	0,59	1,43
1,17	1,55	1,50	1,53	0,40	1,20	1,61	0,86	1,38
1,66	0,86	0,80	0,76	1,61	1,35	1,54	0,47	1,06
1,67	0,95	1,31	1,12	0,49	1,31	0,96	0,66	0,78
0,78	1,12	1,96	0,93	0,51	0,95	1,91	0,51	1,07
0,60	1,51	0,38	1,30	0,98	0,78	1,64	0,75	1,74
0,95	1,00	1,16	0,63	1,47	0,51	0,76	1,66	1,42

1,59	0,85	0,36	0,74	1,09	1,23	1,14	0,86	1,06
1,94	0,39	1,84	0,57	1,18	1,20	0,97	1,65	1,62
1,37	1,33	1,97	0,44	1,62	1,78	1,72	0,66	0,99
1,85	1,83	1,61	1,48	1,66	1,36	1,70	0,40	1,54
1,04	0,96	0,93	0,54	0,95	1,10	1,43	1,94	1,40
1,74	1,41	1,99	1,44	1,23	1,03	1,85	1,59	1,83
1,91	0,94	0,64	1,58	0,55	0,39	0,80	0,46	1,05
1,81	1,76	1,18	0,52	1,23	1,77	1,95	0,58	1,04
0,39	1,13	1,09	1,07	1,40	0,40	1,67	1,11	1,58
0,80	0,33	1,74	0,44	1,12	0,92	0,73	0,91	0,82
1,24	1,25	0,75	1,14	1,59	1,96	1,01	0,67	0,83
0,52	0,49	1,43	1,07	0,74	1,78	1,34	0,77	1,51
0,68	1,42	1,30	0,98	0,38	1,60	0,43	1,59	0,45
0,92	1,23	1,17	0,34	1,22	1,18	0,92	0,38	0,85
1,68	1,18	0,39	0,60	1,09	1,96	1,06	0,88	1,53
1,66	0,67	1,78	0,74	0,80	0,63	1,71	0,88	1,85
1,03	1,23	1,34	0,49	1,40	0,44	1,23	1,79	1,39
0,55	0,50	0,56	0,93	0,43	1,79	1,23	0,73	1,57
1,07	1,27	0,87	0,79	0,55	1,53	0,42	0,31	0,45
1,66	1,37	1,01	0,67	1,53	0,78	1,01	0,77	0,71
0,35	0,53	0,90	0,42	0,75	1,23	0,58	1,28	1,05
0,91	0,40	1,40	0,56	0,92	1,91	1,90	1,18	1,74
1,41	0,38	0,41	0,53	0,55	1,73	0,72	0,92	1,10
1,24	0,95	0,33	0,81	1,48	1,60	1,02	1,48	1,49
0,41	0,64	0,40	1,35	0,48	1,11	0,98	0,93	1,73
1,30	0,39	1,27	0,78	0,67	0,66	0,43	1,49	0,69
0,45	0,34	0,51	0,32	1,85	1,98	0,83	0,89	1,18
0,99	0,85	1,63	1,34	1,37	1,04	1,82	1,94	1,84
0,35	0,93	0,47	0,51	1,54	1,79	1,74	1,54	1,66
1,72	0,99	1,12	1,21	1,79	1,63	1,42	0,49	1,28
1,82	0,91	1,02	0,99	0,58	0,55	1,43	0,89	1,27
0,85	1,94	1,54	1,57	0,60	1,21	0,47	1,37	0,50
0,60	0,36	1,65	0,97	1,08	1,14	1,10	1,53	1,45
0,87	1,01	1,69	0,98	1,82	1,95	0,85	0,81	0,90
1,67	1,72	0,84	1,04	1,62	1,61	1,22	1,57	1,34
0,71	0,76	0,59	0,66	1,08	1,09	1,47	0,95	1,13
1,90	1,12	1,80	0,59	0,94	1,16	0,41	0,36	0,38
1,46	1,22	0,55	1,15	1,92	1,66	1,49	1,12	1,55
0,80	1,62	1,67	1,22	0,42	1,15	1,46	1,91	1,90
1,69	1,26	0,56	1,26	0,72	1,09	1,23	0,85	1,65
1,17	0,73	1,32	0,50	0,89	1,80	1,12	1,64	1,37
1,75	0,49	1,60	1,29	1,81	0,87	0,93	1,70	1,45
1,15	1,71	1,67	1,39	0,71	1,44	1,43	0,96	1,52
1,54	0,84	0,83	1,33	0,52	1,20	1,92	1,86	1,80
1,14	0,88	0,62	0,46	1,14	0,89	1,43	1,72	1,82
0,54	1,92	1,56	1,96	0,87	0,60	1,27	1,19	1,97
0,33	1,79	1,70	1,49	1,56	0,61	0,56	1,45	1,01

0,88	0,67	0,91	1,24	1,25	0,40	0,88	1,74	1,54
1,29	0,31	1,71	0,74	0,53	0,86	1,60	1,05	1,93
0,84	1,07	1,27	1,62	1,01	0,46	0,39	1,85	0,78
1,37	0,82	1,10	0,41	1,96	1,46	1,75	1,06	1,94
1,98	1,85	0,96	1,46	1,66	0,71	1,52	1,22	1,55
1,96	1,19	1,67	1,16	1,80	1,40	1,58	0,61	1,33
1,16	0,60	1,15	0,45	1,31	0,65	1,83	0,39	1,45
0,98	0,78	1,47	1,14	0,43	0,89	1,11	0,67	1,11
0,78	0,89	1,85	1,18	0,75	1,62	1,28	1,28	1,56
0,33	0,81	1,28	1,51	0,38	0,75	1,72	1,23	1,97
1,39	0,92	1,56	0,40	1,01	1,00	0,78	1,79	1,24
1,80	1,55	1,05	0,92	1,06	1,61	1,90	1,44	1,50
1,76	1,27	0,60	0,95	1,06	1,99	1,55	1,88	1,55
1,52	1,57	1,14	0,84	1,05	1,35	1,17	0,97	1,58
1,72	1,46	1,72	1,17	1,03	1,24	1,94	0,32	1,18
1,24	1,02	0,83	1,38	1,05	0,39	1,82	0,65	1,42
1,51	1,81	0,60	1,29	1,55	1,73	1,21	0,39	0,99
0,55	1,48	0,57	1,22	0,64	1,27	0,96	1,34	1,15
1,77	0,51	1,37	0,50	0,84	0,65	1,38	1,13	1,40
0,50	1,04	1,61	0,95	1,70	1,21	1,86	0,97	1,40
0,40	0,78	1,02	1,91	0,66	0,31	1,03	1,63	1,67
0,39	1,67	0,85	1,12	1,61	0,99	0,97	1,11	1,20
1,13	1,92	1,51	1,82	0,41	0,61	1,37	0,62	1,72
0,33	1,87	0,90	1,29	1,76	0,74	1,99	0,49	1,92
1,57	0,31	1,32	0,85	1,44	0,39	1,40	1,98	1,46
1,39	1,84	1,96	1,80	0,97	0,56	1,25	0,52	1,10
0,69	1,78	1,82	1,13	1,61	0,79	1,55	0,61	1,66
0,59	1,48	0,32	1,46	0,64	1,59	1,81	1,90	1,87
1,70	1,49	1,60	0,87	0,74	1,75	1,20	1,12	0,85
0,79	0,51	0,90	0,94	1,53	1,30	1,24	1,50	1,39
0,97	1,60	1,64	0,69	1,34	1,09	0,90	1,12	0,67
0,73	0,31	0,34	0,59	1,02	0,61	0,31	1,70	0,67
1,06	0,86	1,95	0,54	1,45	1,87	0,90	1,88	0,92
0,41	1,33	1,94	1,41	1,43	0,67	0,91	0,64	0,88
1,97	0,49	0,42	0,75	1,11	0,77	1,50	0,67	1,12
0,71	0,82	1,90	1,03	1,67	1,29	1,09	1,84	1,10
0,65	0,43	0,53	0,53	1,83	0,81	0,78	1,90	1,18
0,32	0,92	0,78	1,15	1,95	1,43	0,67	1,48	1,53
1,40	2,00	1,93	0,89	1,81	0,81	0,48	1,49	0,78
0,86	1,31	1,61	0,67	0,31	1,22	1,83	0,78	1,39
1,36	1,84	1,32	0,79	1,23	1,03	1,60	0,60	1,12
0,73	1,10	1,95	0,70	0,87	1,87	1,59	0,77	1,64
1,12	0,35	1,51	0,46	1,16	1,61	1,85	1,46	1,78
1,04	1,60	1,68	1,38	0,34	0,50	1,90	1,96	2,00
0,33	1,88	0,66	1,92	1,71	0,70	1,61	1,48	1,95
1,31	1,60	1,84	0,92	1,20	1,59	0,54	0,69	0,52
0,62	0,31	0,73	1,26	0,66	0,34	1,11	1,17	1,75

0,31	1,16	0,71	0,81	1,22	0,83	1,65	1,47	1,92
1,93	1,03	1,96	1,03	0,84	0,50	1,73	0,87	1,89
0,58	0,78	1,39	0,96	1,28	0,81	0,90	0,40	1,09
1,52	0,86	0,43	0,72	1,22	1,33	1,68	1,01	1,26
0,90	1,54	0,84	0,87	1,63	1,79	0,48	1,70	0,49
1,97	0,94	0,76	0,43	1,34	1,20	0,66	0,98	0,68
1,48	0,35	0,49	1,00	1,27	1,19	0,64	0,64	0,89
1,20	0,54	0,32	0,41	1,77	0,61	1,26	0,82	1,01
1,99	1,48	1,90	0,85	0,77	0,77	0,97	0,84	1,12
0,41	1,08	1,33	0,62	0,52	0,70	1,27	1,40	1,93
0,52	0,47	1,75	0,30	0,78	0,83	1,08	1,66	1,08
1,94	1,54	0,39	0,87	0,47	0,64	0,58	1,99	0,98
0,37	0,33	0,46	0,72	0,51	1,71	0,83	1,59	1,56
1,72	0,32	1,52	0,94	1,70	0,61	1,36	0,76	1,20
1,46	1,96	1,36	1,33	0,34	1,90	0,54	1,30	0,70
1,15	0,91	0,74	1,08	1,68	1,13	1,97	1,77	2,00
1,68	0,57	1,86	1,08	1,98	1,28	1,60	1,60	1,63
0,48	1,00	1,30	0,71	0,93	1,78	0,71	0,97	1,07
1,18	0,66	0,43	0,82	0,74	0,71	1,10	1,33	1,01
0,71	0,82	1,41	0,42	0,83	1,96	1,18	0,98	1,44
1,83	0,92	1,72	1,00	1,02	0,30	1,35	1,36	1,92
1,27	1,61	1,51	1,01	1,71	1,30	1,07	1,87	1,10
1,98	1,19	0,77	1,73	1,80	0,56	0,68	1,32	0,91
1,35	0,98	1,31	0,38	1,51	1,82	1,13	1,61	1,02
1,98	1,13	1,58	0,61	1,99	1,54	1,46	0,51	1,29
0,59	0,85	1,12	0,79	1,98	1,51	0,45	1,48	0,91
1,17	0,37	1,19	0,77	0,68	0,69	1,31	1,40	1,17
0,94	1,51	1,71	1,48	0,97	0,41	0,62	1,99	1,44
0,91	0,42	0,54	1,06	0,54	0,61	1,14	1,84	1,58
1,93	1,56	0,53	0,86	0,86	0,73	1,21	1,56	1,46
1,19	1,70	1,48	1,30	1,64	1,25	0,73	1,57	0,96
0,96	1,84	0,95	1,09	0,53	1,37	1,27	1,79	1,21
0,76	1,53	1,01	1,16	0,64	1,09	0,55	0,49	0,66
0,85	1,73	1,78	1,25	0,99	0,85	0,73	1,67	1,35
1,35	1,00	1,88	0,52	0,96	0,38	1,00	1,36	1,08
0,99	1,17	0,32	1,51	1,13	0,65	0,78	1,49	1,19
0,43	1,06	0,84	0,42	1,26	1,82	1,50	0,77	1,68
0,37	0,46	2,00	0,44	1,66	1,13	1,00	1,33	1,70
1,09	0,63	1,79	0,38	1,85	1,06	0,88	0,64	0,75
1,73	1,05	1,89	1,56	0,37	0,63	0,91	1,14	1,19
1,44	1,16	0,71	0,67	0,82	1,12	0,47	1,02	0,51
1,16	1,42	0,97	1,38	0,85	1,74	1,73	0,35	0,92
0,62	0,97	1,26	1,62	1,78	0,41	0,84	1,84	1,49
0,66	0,62	0,83	1,07	1,92	1,84	1,03	1,05	1,09
1,01	1,24	1,30	0,83	1,76	1,24	1,77	0,60	1,96
1,37	1,97	1,72	1,71	1,17	0,87	1,83	1,70	1,98
1,63	0,43	0,69	0,83	1,26	0,58	1,67	0,63	1,59

1,12	1,27	0,76	0,58	0,44	0,91	0,69	0,95	0,70
0,75	1,89	0,37	1,56	1,00	1,41	1,60	0,43	1,20
1,38	0,98	1,50	0,84	0,39	0,54	0,83	0,74	0,65
1,81	0,62	1,58	1,09	1,58	0,58	0,58	0,38	0,44
2,00	0,59	1,08	1,28	0,59	1,06	1,93	0,44	1,91
1,09	1,53	0,68	1,45	1,26	1,71	1,57	0,38	1,25
0,33	0,64	0,91	0,95	1,82	1,98	0,55	1,79	1,31
1,87	1,56	0,54	1,17	1,47	1,01	0,97	0,93	1,29
0,95	0,96	1,76	0,35	1,48	1,21	1,82	1,10	1,57
1,32	1,26	0,55	1,33	0,46	1,70	0,49	1,35	0,75
1,88	0,91	0,31	0,85	0,79	1,23	0,80	0,67	0,88
1,89	0,46	0,88	0,75	0,70	1,17	0,69	1,34	1,38
1,49	1,60	1,08	0,95	1,16	1,24	1,93	0,59	1,19
1,33	1,03	0,57	0,53	0,30	1,66	1,80	0,39	1,30
1,75	1,40	1,39	1,07	1,71	1,99	1,38	1,85	1,64
0,60	1,51	0,36	1,42	1,98	0,31	1,05	0,82	1,03
0,32	1,41	0,50	1,99	0,33	0,45	1,07	0,73	1,48
1,98	0,67	0,74	0,31	1,05	1,86	0,70	1,16	0,73
1,10	1,91	1,12	1,10	1,29	1,50	1,76	1,03	1,53
0,74	0,84	1,25	1,57	0,32	0,34	1,14	1,42	1,37
0,46	1,78	1,53	1,26	1,10	1,75	0,90	1,47	1,49
1,34	1,69	0,46	1,45	1,17	1,86	0,99	1,37	1,14
1,00	1,75	1,30	1,11	1,05	1,19	0,62	1,06	1,03
1,97	0,45	1,09	0,99	1,01	0,38	1,30	1,97	1,60
0,76	1,05	0,33	1,44	1,21	0,85	1,69	1,44	1,98
1,76	0,79	0,33	0,72	0,54	0,93	1,19	1,32	1,81
1,48	1,76	0,65	1,92	0,49	0,68	0,47	1,26	0,64
1,17	1,67	0,45	1,01	1,86	0,96	1,20	1,42	1,95
1,27	1,31	0,48	1,23	1,41	0,58	0,37	1,72	0,52
0,36	0,90	1,33	0,86	0,78	1,55	1,33	1,27	1,74
1,09	0,66	1,78	0,80	0,44	1,68	0,65	0,58	0,81
1,29	0,99	0,51	1,67	1,01	0,46	0,56	1,19	1,09
1,49	0,98	1,32	0,72	0,32	0,42	0,89	1,84	0,91
1,11	1,99	0,45	0,69	1,07	1,64	0,67	0,62	0,67
0,52	0,72	0,71	0,53	0,30	1,47	1,91	0,62	0,98
1,22	0,83	1,75	0,96	0,47	0,76	0,93	1,52	1,56
1,54	1,28	1,56	0,93	1,67	0,32	1,78	1,31	1,52
1,07	1,24	1,07	0,55	1,28	1,30	0,61	1,82	0,98
1,12	0,44	1,92	1,04	0,68	0,71	1,49	1,20	1,87
1,96	0,62	1,61	0,30	1,46	1,29	1,79	0,80	1,64
1,74	0,78	1,64	1,07	0,78	0,41	0,55	1,18	0,81
1,80	1,81	1,40	1,32	1,41	0,88	0,48	1,81	0,67
1,03	1,18	1,22	1,03	0,92	1,01	1,70	1,00	1,84
1,76	0,88	1,47	1,50	1,62	0,71	1,11	1,05	1,11
1,23	1,99	1,42	0,99	1,83	0,75	1,61	0,86	1,87
0,59	1,57	0,98	0,88	1,12	0,99	1,62	0,65	1,23
1,52	1,69	0,42	0,63	1,86	0,89	1,12	1,07	0,91

0,38	1,51	0,59	0,58	0,67	1,41	1,62	0,49	1,11
1,56	1,29	0,96	1,40	0,56	1,73	1,65	1,08	1,49
1,19	0,67	1,15	0,81	0,50	0,45	0,52	0,60	0,45
0,44	1,85	1,90	1,54	1,74	0,59	1,06	0,77	1,25
1,68	1,34	1,52	1,40	1,06	0,82	1,45	1,52	1,86
1,64	1,97	1,35	1,86	1,89	0,54	1,64	0,82	1,35
0,35	0,79	0,46	0,78	1,11	0,76	1,66	1,91	1,78
1,15	1,20	0,95	0,82	1,49	0,55	0,68	1,87	0,82
1,93	0,47	0,72	1,04	1,03	1,79	0,72	1,98	1,68
1,81	1,15	0,72	0,54	1,85	1,18	1,66	1,09	1,73
1,89	1,64	1,39	1,87	1,47	0,54	1,41	1,51	1,34
0,66	1,03	1,25	1,10	1,00	0,71	0,41	1,11	0,72
1,54	1,46	0,76	1,66	0,95	1,00	0,67	1,81	1,44
1,66	1,02	0,85	1,01	0,49	0,59	1,29	0,93	1,70
1,03	1,11	1,44	0,84	1,64	1,17	1,62	0,72	1,05
1,61	1,12	0,71	1,10	0,39	1,92	1,53	0,71	1,63
1,04	1,78	1,91	1,01	1,74	1,92	1,47	0,88	1,94
1,50	0,68	0,87	0,89	1,52	1,64	1,08	1,69	1,52
1,93	1,02	0,77	0,90	0,63	1,31	1,41	0,52	1,07
1,45	0,90	1,99	0,63	0,92	1,08	1,20	1,66	1,11
1,10	1,33	1,89	1,03	1,30	1,81	1,33	0,75	1,34
1,88	1,47	0,89	1,02	1,30	0,70	0,52	0,63	0,60
1,47	1,90	0,44	1,41	1,10	0,33	0,86	1,38	1,22
1,92	1,97	1,57	1,32	1,35	0,75	1,27	0,33	0,67
0,51	0,69	0,86	1,09	1,17	0,40	1,46	1,97	1,80
1,10	1,43	1,38	0,85	0,67	0,75	0,95	1,93	0,96
0,86	0,91	1,94	0,82	1,06	1,76	1,13	1,63	1,31
1,87	0,40	0,96	0,52	1,87	1,04	1,33	1,43	1,77
0,58	1,17	0,85	1,13	1,66	0,81	0,82	1,68	1,20
1,23	1,41	0,77	0,82	1,49	1,02	1,21	0,65	1,31
0,83	1,04	1,22	1,41	1,72	0,45	1,44	0,35	0,94
0,31	1,43	1,83	0,92	1,95	1,33	1,15	0,37	0,61
0,68	1,02	0,31	1,02	0,73	1,60	0,63	1,91	1,22
0,67	1,96	1,46	1,40	1,76	0,97	1,01	0,96	0,93
0,94	1,86	1,90	1,51	0,60	1,39	0,35	1,41	0,41
1,26	1,07	1,28	0,65	1,60	1,05	1,24	1,42	1,67
1,14	1,61	1,21	1,30	0,89	1,34	1,88	0,56	1,29
1,22	1,41	1,99	0,73	1,54	1,05	0,89	0,74	0,69
1,02	1,19	0,40	0,97	1,50	1,13	1,65	1,44	1,83
1,57	1,23	0,84	1,32	1,51	0,75	0,96	1,03	1,33
1,61	1,79	1,78	1,42	1,70	0,87	0,51	1,75	0,50
1,80	1,55	1,46	0,91	1,43	0,67	0,46	1,19	0,53
0,84	0,98	1,01	0,54	1,24	1,10	1,59	1,20	1,49
1,59	0,30	1,26	0,39	0,59	1,23	0,68	1,39	0,93
0,67	1,40	1,47	0,49	1,96	1,14	0,89	0,76	0,95
1,57	1,30	1,48	0,87	1,29	1,38	1,15	1,83	1,24
0,74	0,99	0,42	0,77	1,61	1,80	0,33	1,70	0,76

0,98	0,84	1,52	1,26	0,43	1,76	1,81	0,55	1,60
0,65	1,99	1,78	1,11	1,62	1,30	1,13	1,23	1,07
0,65	1,91	1,75	0,91	0,31	0,31	1,39	0,94	0,91
0,75	0,60	0,39	0,68	1,35	1,84	0,47	1,89	1,03
1,63	0,89	1,23	1,32	0,63	0,53	0,98	0,47	0,79
1,58	0,89	0,37	0,40	1,49	0,94	1,25	1,46	1,78
0,91	0,52	0,48	0,40	0,97	1,12	1,62	1,00	1,30
1,92	0,91	0,69	1,40	1,00	0,56	1,31	0,49	1,55
0,44	0,55	0,88	0,37	0,76	0,49	0,80	1,48	0,78
0,46	1,06	1,10	1,61	1,86	0,73	0,90	1,13	1,86
1,05	1,28	1,54	0,95	0,92	0,97	0,93	1,32	1,49
0,74	1,63	1,73	1,08	1,68	1,68	1,90	0,38	1,17
1,69	1,66	0,72	0,97	0,66	1,16	0,95	1,51	1,64
0,38	0,55	1,35	1,21	1,99	1,45	1,54	0,53	1,79
1,86	1,04	0,85	0,91	1,44	1,01	1,70	0,53	1,69
0,51	1,83	1,13	1,34	1,50	1,92	0,57	0,49	0,55
1,56	1,64	0,38	1,49	1,23	1,67	0,84	1,72	1,83
1,62	1,79	0,80	1,42	1,30	0,34	1,91	0,40	1,95
1,71	0,74	0,38	1,57	1,27	0,36	1,33	0,95	1,27
0,43	1,67	1,46	0,75	1,74	1,21	1,98	0,74	1,95
0,71	1,32	1,25	0,62	1,34	2,00	1,36	1,26	1,24
1,91	1,49	1,34	0,57	1,59	1,94	0,95	1,71	1,26
1,09	0,33	0,98	0,59	1,99	0,80	1,07	1,08	1,23
1,46	0,39	0,76	0,99	0,53	1,24	0,80	1,93	1,62
1,56	0,38	0,70	1,07	1,50	1,85	1,36	0,94	1,53
1,68	0,32	1,94	0,77	1,07	1,29	1,18	1,06	1,78
1,49	0,81	0,46	1,39	0,95	0,56	1,32	0,55	0,98
0,74	0,69	0,37	1,01	1,54	0,87	1,03	0,90	1,26
1,59	0,93	0,88	0,68	0,91	1,94	0,93	1,04	1,29
0,89	0,35	0,34	0,78	0,95	1,48	1,01	1,41	1,46
1,90	0,85	1,00	1,02	0,48	1,45	1,13	0,47	0,78
1,84	1,23	1,59	1,56	0,68	0,36	1,57	1,45	1,39
0,91	1,02	0,77	1,52	0,61	0,66	1,78	1,61	1,81
1,16	0,36	0,95	1,25	1,46	0,59	1,42	1,21	1,43
0,57	0,73	1,52	0,59	0,75	1,13	1,52	0,65	1,44
1,31	1,59	1,12	0,86	0,95	0,52	1,62	0,67	1,15
1,76	0,82	1,12	0,51	1,72	0,60	0,46	1,24	0,85
1,96	1,23	1,98	1,06	1,97	1,33	1,71	1,25	1,40
0,48	1,27	1,50	1,15	0,52	1,90	0,79	1,82	0,96
1,61	1,82	0,30	1,56	0,36	0,88	1,33	1,15	1,18
1,27	0,81	0,93	1,17	0,82	1,08	0,56	0,69	0,59
0,43	0,95	0,62	1,08	1,07	1,61	1,04	0,77	1,34
1,92	0,90	1,23	1,08	0,64	1,65	1,44	1,15	1,28
1,82	1,95	1,37	0,84	1,79	0,45	1,58	0,47	1,22
0,61	1,51	1,30	1,47	1,82	1,04	1,16	1,61	1,48
1,71	1,36	1,96	1,23	1,78	1,22	1,54	1,05	1,94
1,30	1,65	1,80	1,61	1,23	0,94	1,49	1,67	1,43

0,62	1,58	1,00	1,48	1,33	1,59	1,52	1,28	1,56
1,19	0,95	0,59	0,68	0,66	0,34	0,79	1,75	1,17
1,16	1,91	1,48	1,35	1,91	0,31	1,78	0,41	1,74
1,55	1,49	1,16	1,24	1,62	1,66	1,61	1,25	1,71
1,95	1,19	1,22	1,15	1,88	1,90	0,53	0,93	0,89
1,37	0,88	1,81	1,03	0,93	0,74	0,62	1,86	1,13
1,79	1,01	0,87	1,57	1,97	0,32	1,42	0,75	1,81
0,92	1,54	1,06	1,67	0,46	0,48	1,25	0,47	1,64
1,74	1,56	1,11	1,12	0,39	1,22	0,86	1,09	1,10
0,62	0,75	0,93	0,93	1,73	1,39	1,37	0,83	1,11
1,21	0,81	1,40	0,32	1,54	1,50	0,59	1,52	0,85
1,50	1,06	1,85	0,98	1,84	1,96	1,98	1,99	1,80
1,80	0,77	1,58	1,86	1,99	0,38	1,91	0,82	1,95
0,81	1,95	1,03	1,53	0,52	1,79	1,03	1,54	1,27
0,66	0,78	1,94	0,36	0,75	0,58	0,34	0,64	0,37
0,86	1,59	0,55	0,86	1,64	1,21	0,75	0,33	0,81
1,25	1,87	1,64	1,51	0,59	0,84	1,57	1,20	1,80
0,44	1,29	1,46	0,76	1,07	0,56	1,44	1,50	1,64
0,87	0,50	2,00	1,54	0,88	0,46	1,71	1,10	1,96
0,90	0,81	1,55	0,80	1,10	1,06	1,48	0,68	1,65
1,45	1,52	0,70	1,15	1,18	1,97	1,53	1,62	1,69
0,85	1,85	0,70	0,65	1,82	1,53	1,16	0,55	1,26
0,85	1,21	0,72	1,93	0,83	0,51	1,74	0,53	1,70
1,17	0,65	1,70	1,17	0,88	1,24	1,45	0,74	1,82
0,99	0,38	1,67	1,06	1,41	0,71	1,72	0,33	1,68
1,01	0,59	0,95	1,03	0,89	0,67	0,52	1,67	1,00
0,79	1,82	0,96	0,99	0,80	0,72	1,40	0,45	1,35
0,54	1,76	0,49	0,70	1,54	0,69	0,42	1,71	0,55
0,69	1,16	0,60	0,61	1,34	1,64	1,87	0,94	1,33
1,02	1,09	0,38	0,67	1,07	0,78	1,67	1,21	1,77
1,13	1,37	1,80	1,84	1,93	0,47	1,66	0,53	1,55
0,53	0,48	1,72	0,48	0,71	2,00	0,35	1,30	0,40
1,86	1,08	1,83	0,47	1,82	1,98	0,59	1,22	0,65

ORd - Control Population - 393 Human Ventricular Cell Models

G_{Na}	G_{NaL}	G_{to}	G_{Kr}	G_{Ks}	G_{K1}	G_{NCX}	G_{NaK}	G_{CaL}
0,50	1,81	0,67	0,69	1,49	1,12	0,92	0,69	0,85
0,98	0,89	1,12	1,18	0,86	1,62	1,35	1,08	1,95
0,78	1,63	1,46	1,15	1,97	1,20	1,33	1,02	1,24
0,61	0,43	1,91	1,39	1,08	1,45	0,88	0,58	0,67
0,61	1,25	1,90	1,53	0,98	0,53	1,49	1,85	1,89
0,68	1,48	1,67	0,51	1,39	1,96	1,08	0,70	0,78
1,76	1,23	0,61	0,86	1,97	1,49	1,49	0,98	1,62
0,73	1,60	1,71	1,24	1,27	1,55	1,29	0,82	1,52
0,94	1,84	0,43	1,06	0,48	1,20	1,33	1,79	1,98
0,66	1,99	1,14	1,41	0,63	1,06	1,39	0,87	1,16
0,74	1,02	1,53	1,10	1,34	1,46	1,09	1,40	1,59
0,70	1,02	1,99	1,26	1,95	1,59	1,86	0,78	1,80
1,10	1,88	0,46	1,33	1,57	1,65	0,86	1,65	1,68
0,86	1,38	1,13	1,15	1,82	1,54	0,85	0,62	1,03
1,77	1,12	0,86	1,21	0,36	1,32	1,20	1,02	1,43
0,78	1,04	0,53	1,71	1,67	0,63	1,35	1,39	1,73
1,51	1,49	0,45	0,88	0,73	0,44	0,98	1,03	1,06
1,21	0,30	0,76	0,41	1,17	1,88	1,24	0,41	1,02
0,77	1,84	1,72	1,08	1,08	0,39	1,90	1,38	1,91
1,66	1,55	0,58	1,10	0,81	1,08	1,57	0,66	1,55
0,50	1,58	1,66	0,55	1,92	0,46	1,66	1,39	1,97
0,58	0,91	1,90	0,96	0,57	1,21	1,15	0,99	0,75
0,57	0,43	1,59	1,12	1,48	0,94	0,79	1,85	1,21
0,60	0,75	1,13	1,07	0,57	0,35	0,75	0,88	0,83
1,22	0,81	1,87	0,66	1,56	0,60	1,78	1,16	1,38
1,40	1,57	0,83	0,69	1,73	1,52	1,00	1,42	1,34
1,02	1,55	1,17	1,64	1,69	1,16	0,71	0,70	0,98
1,04	1,79	0,47	1,10	1,82	1,06	0,90	1,91	1,78
0,75	1,42	1,77	0,54	1,95	0,53	1,45	1,47	1,10
1,83	1,58	1,72	1,67	0,53	0,58	1,14	1,60	1,68
1,30	1,32	1,20	1,22	0,40	1,35	0,91	1,79	1,10
1,33	1,41	1,72	0,83	1,91	0,39	0,52	1,33	1,03
0,81	1,77	0,82	1,13	1,14	0,60	1,53	0,89	1,37
0,71	1,96	1,09	1,47	1,75	0,63	1,96	1,25	1,64
1,43	1,78	1,80	1,58	1,79	0,60	1,85	0,79	1,40
0,51	1,58	1,19	1,48	1,75	1,01	1,06	0,59	0,71
1,66	1,77	1,76	1,19	1,26	1,54	1,42	0,57	1,81
1,38	0,31	1,47	0,53	0,74	0,79	1,18	1,83	1,77
0,89	1,59	1,24	0,65	0,75	1,86	1,61	1,48	1,43
0,46	0,94	0,75	1,02	1,99	1,52	0,85	1,47	1,45
0,79	1,16	1,49	0,87	1,25	1,44	0,62	1,51	0,97
0,52	1,50	1,73	1,45	1,33	0,89	1,91	1,94	1,86
1,44	0,55	0,32	0,94	0,76	1,55	1,25	1,09	1,67
0,71	1,05	1,41	1,46	1,01	0,67	1,09	1,84	1,26

1,72	0,41	1,74	0,60	0,50	0,98	1,20	1,42	1,85
1,62	1,95	1,06	0,63	2,00	1,66	1,83	0,45	1,58
1,60	1,85	1,59	1,46	0,39	0,73	0,39	0,50	0,43
0,68	0,49	1,13	1,51	1,24	1,05	0,98	1,56	1,58
0,86	1,16	0,43	0,75	0,55	1,11	0,87	1,73	1,45
0,71	1,32	1,21	0,45	1,48	0,88	1,51	0,92	1,12
1,57	0,55	0,72	0,47	1,89	0,64	0,49	0,71	0,77
0,89	1,01	0,42	0,87	1,14	1,83	1,77	0,32	0,88
0,96	0,57	1,22	1,58	0,48	1,42	1,65	0,41	1,53
1,70	1,94	0,47	1,69	0,72	0,70	1,62	0,35	1,34
1,82	1,02	1,91	1,72	0,68	1,34	1,45	0,84	1,74
1,56	0,60	1,38	1,21	1,58	0,38	1,58	0,46	1,17
1,48	1,96	1,81	0,84	1,21	0,78	1,07	1,48	1,80
0,81	0,63	1,92	1,45	1,53	0,53	0,84	0,36	0,67
1,02	0,31	0,36	1,29	1,92	0,41	0,77	1,42	0,93
1,21	1,17	0,93	1,55	1,64	1,00	1,51	1,01	1,50
1,84	1,64	1,97	0,65	1,92	0,54	1,69	0,65	1,38
1,30	1,54	1,74	0,58	0,67	1,00	1,53	1,45	1,37
1,33	1,21	1,28	1,92	1,35	0,33	1,00	1,14	1,25
1,44	1,58	1,44	1,68	0,72	1,63	1,94	0,32	1,85
1,92	1,13	1,38	1,00	0,64	1,69	1,10	0,73	1,61
1,97	0,70	1,71	1,07	0,75	1,46	0,66	0,48	0,66
1,25	1,16	0,44	0,87	1,62	0,73	0,87	1,43	0,97
1,72	0,89	0,96	1,62	1,24	0,34	1,34	0,63	1,82
1,44	1,85	0,91	1,51	1,28	1,84	0,74	0,71	0,76
1,65	0,73	1,98	1,34	1,51	1,12	1,27	0,33	1,52
1,90	1,12	1,60	1,82	1,05	0,78	1,88	0,57	1,97
1,31	0,79	1,34	1,04	0,33	2,00	1,42	1,89	1,51
0,53	0,65	1,45	1,67	1,16	0,86	1,57	1,33	1,26
1,34	1,23	1,74	1,04	1,23	1,17	0,91	0,95	0,95
1,60	1,72	1,17	0,96	1,39	0,99	1,03	1,33	1,37
1,77	0,44	1,24	0,41	0,54	1,82	0,81	1,82	1,70
1,06	0,85	0,78	1,14	0,91	1,44	1,84	0,80	1,35
1,33	1,42	1,66	0,60	0,72	1,07	1,72	1,24	1,86
0,65	0,91	1,01	1,41	0,51	0,70	1,77	0,41	1,05
1,75	1,88	1,87	1,51	1,22	0,76	0,82	1,98	1,21
0,54	1,14	0,93	1,25	1,65	0,44	1,77	1,48	1,63
0,64	0,62	0,50	1,08	0,44	0,34	0,89	1,30	0,92
1,56	0,68	0,49	1,40	0,93	0,61	0,99	0,62	1,19
0,95	0,55	0,57	1,85	1,74	0,30	1,63	0,49	1,93
1,48	1,06	1,06	0,89	1,54	0,42	1,07	1,05	1,39
1,37	1,75	1,95	0,91	0,56	0,62	1,29	1,37	1,58
1,97	0,63	1,60	1,53	1,74	1,58	1,38	1,38	1,98
1,90	0,43	1,88	1,64	1,32	0,57	0,81	1,73	1,73
1,92	0,80	1,65	0,40	1,31	1,80	0,44	1,85	1,11
0,49	1,99	0,57	1,57	0,50	0,86	1,04	1,19	1,54
1,84	0,46	1,26	0,55	1,37	1,88	1,02	1,69	1,82

0,56	0,63	0,95	1,61	0,96	1,36	1,30	0,62	1,64
1,15	1,46	1,58	0,81	0,70	1,70	1,74	0,92	1,95
1,43	0,36	1,32	1,37	1,88	1,39	1,17	1,20	1,64
0,67	1,19	0,89	0,78	1,32	0,39	0,89	1,98	1,37
0,99	1,31	1,08	0,62	1,43	1,06	1,22	1,30	1,40
1,58	1,47	1,16	0,64	1,81	1,44	1,99	1,39	1,82
1,73	0,85	0,85	1,42	1,50	1,91	0,96	1,89	1,63
1,20	0,79	1,03	0,67	1,84	0,69	0,81	0,34	0,88
1,77	1,52	0,74	1,13	1,45	0,64	1,03	1,03	1,68
1,92	1,33	0,73	1,12	1,13	1,15	0,38	0,78	0,58
0,45	1,73	0,47	0,93	0,43	1,47	1,22	1,86	1,90
1,28	1,53	0,80	1,73	1,86	0,35	0,87	0,62	1,23
1,56	1,63	1,00	0,66	1,36	1,02	0,66	1,63	1,44
1,74	0,44	1,48	0,81	1,25	0,32	1,28	1,00	1,42
0,58	1,57	1,06	0,92	1,49	0,85	0,62	0,58	0,40
1,34	1,47	0,53	0,72	1,39	0,69	0,61	1,21	0,90
1,99	1,16	1,09	0,54	0,43	0,86	0,89	1,81	1,57
0,64	1,26	1,48	1,23	1,04	0,88	0,88	1,42	1,46
1,88	1,73	1,94	0,69	1,96	1,75	1,04	0,54	0,95
0,64	1,92	0,45	1,31	1,38	0,91	0,70	1,11	1,23
1,56	1,67	1,58	1,43	1,11	1,46	1,56	1,18	1,71
1,66	1,38	1,92	0,84	1,18	0,70	0,56	1,81	1,01
1,54	0,83	0,44	1,80	1,18	0,66	0,71	0,77	1,40
0,63	1,91	1,58	1,02	0,56	1,03	1,67	0,33	1,02
0,95	0,36	1,14	0,46	0,49	0,63	0,62	1,66	0,99
1,90	1,64	1,81	1,79	0,83	0,51	1,63	0,44	1,29
0,85	1,13	0,42	1,76	0,44	0,79	0,97	1,85	1,10
1,75	1,72	1,27	0,84	0,98	1,78	1,61	0,94	1,49
1,11	0,87	1,78	1,17	1,63	1,55	1,21	0,96	1,33
0,97	0,73	0,74	1,52	1,21	0,74	0,75	1,77	0,85
0,76	1,62	0,81	0,69	0,99	1,92	1,12	0,51	1,07
1,38	1,30	0,86	1,17	0,78	0,75	0,76	0,86	1,42
1,40	1,18	0,73	0,49	1,95	1,84	1,90	0,52	1,84
1,18	0,41	1,16	1,59	1,59	0,93	0,93	0,90	1,30
0,82	1,84	0,48	1,37	0,71	0,41	1,29	1,32	1,67
1,08	1,03	1,11	1,24	1,35	1,54	1,07	1,99	1,23
1,40	1,74	1,91	0,42	0,80	1,77	0,49	1,00	0,65
1,35	0,44	0,79	0,62	0,78	1,36	1,46	1,82	1,63
0,44	0,84	0,68	0,95	1,65	0,62	1,12	1,30	0,77
1,26	0,55	0,58	0,78	1,34	1,89	1,75	0,63	1,17
0,69	1,10	0,80	1,18	1,51	1,13	1,57	0,86	1,34
1,81	0,62	1,58	1,57	1,26	1,14	1,20	0,97	1,28
1,49	1,56	1,31	1,91	1,14	0,88	1,75	0,61	1,76
1,80	1,59	1,63	1,54	0,67	0,67	1,82	0,36	1,24
0,84	0,79	0,34	1,27	1,55	0,99	1,12	1,54	1,67
1,36	1,83	1,39	0,72	1,43	1,12	1,33	1,64	1,79
0,53	0,50	1,54	0,48	1,73	0,83	1,83	1,11	1,19

1,09	1,46	1,12	1,22	1,32	1,73	1,96	1,34	1,72
0,53	1,25	0,47	0,58	0,79	0,75	0,71	1,95	0,88
1,32	1,61	1,25	1,58	0,94	0,44	0,96	1,53	1,61
1,02	0,33	1,83	0,78	0,77	1,57	1,26	0,69	1,33
1,18	0,34	1,76	1,32	1,57	1,30	0,45	1,92	0,97
1,23	0,92	0,76	1,25	0,96	1,41	1,24	1,19	1,55
1,02	0,69	1,91	0,83	0,87	1,16	0,32	1,20	0,41
1,28	1,30	1,60	1,88	0,34	0,78	1,10	0,62	1,84
1,31	1,05	0,65	0,91	1,28	1,95	0,85	0,34	0,93
1,88	0,98	1,55	0,78	0,48	1,02	0,74	0,92	0,89
1,10	1,02	1,57	0,70	0,82	1,35	0,44	1,59	0,82
0,91	1,87	1,37	0,79	1,92	0,83	1,26	1,41	1,34
1,64	0,32	1,51	0,75	0,35	0,67	1,88	1,09	1,74
1,98	1,58	1,06	1,76	1,71	1,45	1,77	1,07	1,93
0,90	0,57	0,75	0,73	0,99	0,36	0,56	1,81	0,74
0,67	1,42	1,11	1,27	1,98	0,96	0,71	0,45	0,75
1,13	0,43	1,00	1,32	0,32	1,42	1,14	0,75	1,62
0,55	1,25	0,67	0,50	1,62	0,86	0,40	1,95	0,44
1,64	0,37	1,37	0,91	0,58	0,83	0,95	0,67	0,99
1,89	1,82	0,52	0,66	1,75	1,93	0,42	0,50	0,72
1,03	1,93	0,80	1,04	0,69	1,75	1,71	0,85	1,93
0,93	0,84	0,50	1,48	0,32	0,43	0,46	1,00	0,92
1,41	0,60	0,57	0,59	0,73	1,28	1,97	1,27	1,86
1,45	1,76	1,61	0,53	1,94	0,98	0,69	1,48	1,13
1,61	1,91	0,84	1,43	1,90	0,61	1,67	1,43	1,97
1,38	0,33	0,63	1,32	1,57	0,51	1,35	1,28	1,75
0,63	1,84	1,68	0,59	0,95	1,81	1,04	1,56	1,39
1,41	0,65	0,51	1,07	0,37	1,29	0,88	0,97	1,42
1,01	1,98	1,89	1,11	1,82	1,99	1,30	1,07	1,82
1,85	1,72	1,85	0,51	0,54	1,88	1,94	0,64	1,37
0,99	0,85	0,77	0,93	1,09	0,58	1,38	0,40	1,44
1,69	0,34	0,87	0,90	0,60	1,87	0,36	1,76	1,04
1,20	0,67	0,61	1,24	0,44	0,84	0,68	1,21	1,44
1,11	1,44	0,93	1,24	1,34	0,64	0,82	1,77	1,70
1,42	1,59	1,09	0,73	0,64	1,84	1,50	0,60	1,48
1,44	0,66	0,76	0,63	1,06	0,89	0,78	1,65	0,99
0,72	1,83	1,69	0,37	1,95	1,69	1,71	1,22	1,50
0,72	1,33	0,89	0,37	1,37	0,85	0,79	1,15	0,62
1,02	1,83	1,60	0,81	1,40	0,49	0,53	1,29	0,79
1,37	0,51	1,66	0,83	0,70	1,09	1,10	1,35	1,51
0,49	1,42	1,04	1,19	0,78	0,54	1,79	0,67	1,15
1,80	0,99	1,83	1,26	1,28	1,44	1,03	0,68	0,97
1,76	1,92	1,28	0,68	0,33	0,76	1,97	0,76	1,53
1,32	1,66	1,62	0,48	1,14	1,33	1,50	0,52	1,18
1,41	1,36	0,70	1,00	0,65	1,05	0,83	1,21	1,60
0,45	1,01	1,89	1,35	1,80	1,42	0,55	1,43	0,57
1,74	1,88	0,53	1,87	0,68	0,97	1,04	1,11	1,51

1,90	1,94	0,70	1,04	1,83	0,46	0,52	0,99	1,12
1,13	1,89	0,48	0,53	0,92	1,95	1,25	1,58	1,32
0,62	1,89	0,32	0,90	1,70	1,82	1,14	1,96	1,99
0,64	0,74	0,34	1,21	1,50	0,76	1,89	1,27	1,92
0,96	1,97	0,58	1,47	1,35	0,43	0,38	1,82	0,56
1,40	1,56	1,77	0,46	0,68	1,86	1,47	1,08	1,39
1,30	0,93	1,70	1,50	1,72	1,08	0,86	0,36	1,15
0,90	0,71	1,57	1,05	0,43	1,75	0,69	1,59	0,89
1,03	1,14	0,84	1,56	0,74	1,04	1,70	1,93	1,88
0,68	1,63	1,01	1,68	0,78	1,42	1,68	0,31	1,17
1,54	1,56	1,64	1,38	0,92	0,93	1,87	1,16	1,74
1,18	0,77	0,53	0,69	1,62	1,52	1,13	1,56	1,78
0,97	1,79	1,32	0,46	1,68	1,95	0,95	1,77	1,24
0,95	0,96	0,37	1,67	0,49	0,61	1,18	0,39	0,99
1,54	0,32	1,29	1,05	0,87	0,95	1,11	0,90	1,77
0,44	1,84	1,17	1,40	0,44	0,58	1,05	1,90	1,37
0,87	0,34	1,56	0,70	0,79	0,96	1,28	0,67	0,92
1,49	0,62	0,50	1,77	1,40	0,94	0,72	1,20	1,66
1,47	1,43	1,99	1,24	1,00	0,30	1,16	0,61	1,03
1,83	0,64	1,55	0,67	1,71	1,72	1,24	0,78	1,83
1,76	1,25	1,66	0,51	1,31	1,22	0,55	1,01	0,71
0,76	0,83	1,44	0,45	0,86	1,39	0,81	1,97	1,47
1,08	0,82	0,57	1,56	0,40	1,65	1,05	1,25	1,75
0,52	1,79	1,80	1,22	0,41	1,38	1,85	1,31	1,70
1,61	0,48	0,63	1,49	0,55	1,68	0,76	1,41	1,42
1,33	1,63	0,83	1,55	1,73	1,75	1,35	0,88	1,21
0,60	0,73	1,75	0,82	0,99	0,84	1,97	0,72	1,43
1,13	1,63	1,31	1,53	0,97	1,41	0,61	1,01	0,60
1,01	0,68	1,18	1,09	1,85	1,96	0,76	1,45	1,09
1,11	1,25	0,97	1,17	1,67	1,06	1,54	1,94	1,90
1,03	0,37	1,36	1,01	1,53	1,47	0,32	1,91	0,63
0,45	1,39	1,89	1,26	0,47	0,36	1,08	0,76	0,96
1,26	0,63	0,94	0,95	1,68	1,50	0,35	1,79	0,82
1,77	0,34	0,62	0,82	0,66	0,52	1,19	1,96	1,75
1,09	1,32	0,97	1,47	1,69	0,80	0,52	1,62	0,87
1,63	1,69	0,51	1,67	0,56	1,62	1,23	1,12	1,33
0,74	1,85	0,82	0,74	0,31	1,86	1,92	1,54	1,43
1,25	0,73	1,06	1,40	1,51	1,02	1,14	0,65	1,83
0,77	1,15	0,50	0,60	1,03	0,92	1,93	0,80	1,52
1,53	0,85	1,29	1,88	1,51	0,50	1,66	0,60	1,40
1,73	1,95	0,43	1,36	1,59	1,10	1,20	1,18	1,71
1,40	1,98	1,61	1,74	1,55	1,05	1,31	1,10	1,67
1,92	0,76	1,64	1,18	1,53	0,43	0,77	1,94	1,31
1,31	1,49	1,60	0,88	1,75	0,68	1,57	1,31	1,34
0,79	0,69	0,75	1,03	0,34	1,01	1,43	0,74	1,65
1,97	1,65	1,29	0,61	0,76	1,49	0,36	1,26	0,74
1,87	1,27	1,88	1,36	1,02	0,81	0,52	0,36	0,72

0,69	1,86	1,05	0,62	1,33	0,40	1,89	0,99	1,36
1,12	1,61	0,57	0,85	0,91	1,13	0,71	1,73	1,15
1,37	1,54	1,80	1,53	0,91	1,43	0,94	1,39	1,15
1,06	1,55	0,53	0,77	1,71	0,53	0,58	1,93	0,84
1,61	1,53	1,92	1,16	1,33	0,81	0,82	0,82	1,09
1,25	1,58	1,26	1,07	0,33	1,54	0,88	1,12	1,08
1,46	1,52	0,94	0,83	1,60	0,90	0,95	1,46	1,80
1,65	0,90	0,89	0,97	1,11	1,10	0,73	0,73	1,21
0,54	1,11	0,64	1,02	1,47	0,40	1,42	1,92	1,55
0,78	1,79	1,48	0,81	1,16	0,41	1,03	1,15	0,93
1,76	1,14	1,67	1,11	1,78	1,16	1,23	0,76	1,42
1,51	1,11	1,30	1,88	1,03	0,78	1,16	1,87	1,64
0,89	1,36	1,94	0,61	1,87	1,29	1,54	0,47	0,96
1,21	1,69	0,46	1,23	1,55	0,85	0,77	0,88	0,86
0,47	0,65	1,21	1,54	0,93	0,82	1,11	0,42	0,79
1,14	0,72	1,07	1,35	1,31	0,42	1,61	0,66	1,88
1,01	1,31	0,44	0,70	1,32	0,31	0,55	1,59	0,76
1,76	0,78	1,32	0,41	0,89	1,31	1,09	1,48	1,31
1,74	1,29	1,42	1,19	1,78	0,98	0,44	1,80	0,77
1,03	0,35	0,93	0,77	0,90	0,52	0,73	1,04	0,75
1,49	0,77	1,64	0,89	1,10	1,58	1,92	0,64	1,53
1,07	1,28	1,63	0,68	0,36	1,74	1,35	0,44	0,90
0,52	1,01	0,90	1,14	0,87	1,22	1,52	1,11	1,59
1,85	1,44	1,83	1,07	0,52	0,66	1,60	0,71	1,30
0,77	0,69	1,78	0,34	1,55	0,94	0,98	1,28	1,33
0,88	0,78	1,06	1,35	1,43	1,31	1,04	0,38	1,14
1,03	0,56	0,38	0,85	0,82	1,46	1,88	0,93	1,48
1,45	1,65	0,79	1,07	1,04	1,52	1,24	1,15	1,54
1,79	1,03	0,82	0,95	0,82	0,76	1,69	0,51	1,50
0,72	0,83	1,68	1,44	0,83	0,41	1,38	1,31	1,46
1,75	0,96	0,57	0,70	0,96	1,12	0,90	1,29	1,43
1,73	1,42	1,64	1,03	0,38	1,19	1,54	1,88	1,86
1,88	1,87	1,04	0,81	1,99	2,00	0,95	0,63	0,91
1,15	0,94	1,59	1,30	1,67	0,96	1,28	0,95	1,77
1,22	1,44	1,21	0,77	0,47	1,62	1,71	0,39	1,13
1,50	1,59	0,66	1,18	0,50	1,63	0,99	0,51	1,05
1,47	0,50	1,76	1,39	0,60	1,78	1,43	1,51	1,50
0,85	1,94	1,15	1,18	1,62	0,33	0,66	1,03	0,99
1,77	1,70	0,77	0,87	1,46	1,81	1,46	1,33	1,91
0,85	1,36	0,71	1,00	1,03	0,67	0,66	1,15	1,22
1,05	0,68	0,41	0,99	0,51	0,76	1,18	0,35	0,94
0,95	1,14	1,20	1,70	2,00	0,71	1,28	0,45	0,91
1,69	1,66	1,48	1,02	1,56	1,72	1,74	0,67	1,52
0,87	1,67	1,54	1,82	1,26	0,80	0,92	1,38	0,92
1,20	1,57	0,90	0,94	1,41	0,38	0,56	0,35	0,56
0,80	1,93	1,04	0,63	1,89	1,29	1,74	0,48	1,07
0,66	0,94	1,10	1,60	0,38	0,60	1,27	1,50	1,14

1,10	1,91	1,08	0,62	1,77	0,69	1,39	1,13	1,96
1,14	1,89	1,00	1,39	1,26	1,47	1,71	0,43	1,83
0,54	1,05	1,62	1,86	1,52	0,69	0,96	1,02	1,53
0,58	1,26	0,30	1,44	1,24	1,12	0,51	1,21	0,89
0,55	0,33	1,76	0,66	1,39	0,41	1,56	0,39	1,08
0,71	0,56	1,24	1,22	1,72	1,01	0,43	1,54	0,84
1,58	1,22	1,79	1,23	1,83	1,29	1,30	0,86	1,33
0,50	1,41	1,39	1,10	1,48	0,55	1,96	1,70	1,64
1,98	0,91	1,76	0,46	1,64	1,91	0,69	0,71	0,83
0,89	1,78	0,85	0,92	0,42	1,61	1,05	1,88	1,48
1,30	0,93	1,98	0,33	1,11	1,81	1,25	0,49	0,77
1,43	0,99	0,99	1,46	0,32	1,88	0,79	1,02	1,47
0,99	0,69	1,46	0,69	0,58	0,74	1,25	1,41	1,04
0,48	1,82	1,26	1,06	0,58	0,93	1,08	1,77	1,63
1,60	0,70	1,07	1,55	1,25	1,07	0,84	0,70	1,21
1,98	1,45	1,19	1,85	1,45	0,99	0,76	0,39	1,31
1,21	1,24	0,40	0,60	0,96	1,27	1,51	1,74	1,56
0,87	1,34	1,44	0,57	0,59	1,49	0,93	1,13	1,03
1,38	0,53	0,88	0,82	1,96	0,45	1,56	1,46	1,88
1,02	1,67	1,04	1,82	0,79	0,52	1,80	0,36	1,68
0,89	1,44	0,31	0,68	0,32	1,07	1,59	0,94	1,69
1,66	1,43	1,27	2,00	0,97	0,55	1,39	0,98	1,98
1,89	0,50	0,49	1,40	1,56	0,49	1,95	0,74	1,91
1,32	1,64	1,94	1,73	0,54	0,81	1,12	1,43	1,75
1,09	1,91	1,63	1,58	1,69	1,56	1,06	1,61	1,63
0,62	1,15	1,14	1,36	0,61	1,09	1,69	0,57	1,52
1,34	0,79	0,38	0,80	1,93	1,15	0,83	1,14	1,49
1,69	0,47	1,05	0,58	1,90	1,34	1,51	0,44	1,44
0,58	1,44	1,53	0,51	1,34	1,55	0,84	1,06	0,64
0,51	0,98	1,77	0,92	0,78	1,42	1,25	0,91	1,49
0,72	0,88	0,81	1,25	0,96	1,09	1,34	1,64	1,41
1,01	0,48	2,00	1,13	0,52	1,63	1,38	1,57	1,93
1,85	0,78	0,48	0,90	1,02	1,77	0,52	1,90	1,22
1,76	1,45	0,89	1,31	1,03	0,64	1,02	0,36	1,19
0,93	1,40	1,68	1,15	1,15	1,70	0,54	1,62	1,13
0,64	0,92	1,79	1,49	1,94	1,37	0,87	1,89	1,64
0,57	0,51	1,28	1,47	0,73	1,50	1,62	1,16	1,88
0,93	1,35	0,73	1,11	1,74	1,71	1,73	1,30	1,96
1,83	0,45	1,10	1,05	1,67	1,84	0,72	1,37	1,66
0,59	1,10	0,77	1,69	1,04	0,44	1,82	0,57	1,38
1,39	0,40	0,61	1,12	0,72	1,19	0,59	0,78	1,01
1,19	0,68	0,52	0,76	0,79	0,43	0,75	1,70	0,97
1,97	1,82	1,80	1,62	1,22	0,64	1,72	0,55	1,52
1,17	1,26	0,42	1,23	1,46	1,42	1,15	1,61	1,41
1,51	0,66	1,71	0,95	1,41	1,37	0,68	1,70	1,34
1,66	0,74	0,44	0,79	0,84	1,22	0,98	1,24	1,61
1,05	1,52	0,45	1,33	1,63	1,73	1,55	0,38	1,75

1,18	1,78	1,95	0,46	1,41	0,98	0,82	1,45	1,07
1,93	0,36	0,46	1,30	0,80	0,92	1,41	1,38	1,75
1,55	1,19	1,69	0,82	0,71	1,02	1,74	0,61	1,59
0,86	1,72	0,59	0,96	0,69	1,97	1,46	1,64	1,74
1,89	0,61	1,79	0,64	1,08	1,53	0,96	0,51	1,23
0,65	0,43	0,46	1,17	0,76	1,19	1,41	0,74	1,56
0,55	1,86	1,20	1,28	0,71	1,66	1,17	0,68	0,75
0,56	1,07	0,55	1,01	1,70	0,51	0,84	1,61	1,29
1,00	1,13	1,74	1,27	1,97	1,78	0,83	1,90	1,74
1,11	2,00	0,96	0,61	1,54	0,57	1,20	0,89	0,89
1,43	1,73	0,91	1,89	1,70	0,35	1,61	1,83	1,90
1,78	0,33	1,46	0,56	0,85	1,37	0,82	1,06	1,44
1,40	0,95	1,02	1,39	1,33	0,49	0,75	1,03	1,57
0,66	1,04	1,77	1,02	1,07	1,92	0,90	1,58	0,77
1,70	1,51	0,44	1,58	0,33	1,84	1,30	0,67	1,73
1,09	1,24	1,41	0,67	0,99	1,03	1,82	0,64	1,06
0,62	1,83	1,83	1,37	0,54	1,48	0,87	1,94	1,55
1,46	0,59	1,09	1,03	1,96	0,52	1,94	0,30	1,65
0,92	0,41	1,22	0,53	0,91	1,89	1,01	1,68	1,90
1,35	1,62	0,93	0,54	1,12	0,91	1,27	1,46	1,97
1,34	1,31	1,02	1,72	1,76	1,02	0,88	1,79	1,14
1,13	1,32	0,48	1,79	1,15	1,18	1,78	0,40	1,51
1,47	0,87	1,40	1,31	1,80	0,71	1,15	0,73	1,56
0,47	0,40	1,76	0,93	1,38	1,32	1,06	1,96	1,51
1,45	1,97	1,32	0,81	1,86	0,75	1,00	1,45	1,27
0,45	0,78	1,49	0,64	0,32	1,94	1,31	1,71	1,20
0,59	1,43	1,35	0,87	1,95	1,50	1,67	1,08	1,48
0,78	1,53	0,46	1,36	1,05	0,33	0,93	1,33	1,88
0,61	0,46	1,20	0,74	0,77	1,06	1,09	1,70	1,38
1,71	1,56	1,50	1,18	0,87	1,32	1,76	1,60	1,95
0,55	1,17	0,97	0,75	0,85	1,57	1,04	0,99	1,16
0,80	0,67	1,47	0,71	0,61	1,98	1,13	0,63	0,87
1,25	1,67	1,34	1,33	1,66	1,66	0,78	0,58	1,20
0,94	0,58	0,31	0,59	0,45	1,30	1,32	1,93	1,32
1,48	1,66	0,60	0,68	1,36	1,27	1,73	0,93	1,61
1,05	0,66	0,83	1,12	0,65	0,48	1,08	0,59	1,29
1,52	1,90	1,05	1,50	1,17	1,34	0,79	0,38	1,16
0,97	1,07	0,94	1,36	0,84	1,70	0,42	1,11	0,51
1,53	1,29	1,55	1,09	1,09	1,30	1,84	0,40	1,44
0,74	1,30	1,27	1,04	1,10	1,65	1,64	1,08	1,69
1,21	1,98	0,47	0,85	1,25	0,38	1,93	0,46	1,23
1,37	1,13	1,52	1,59	1,11	1,17	1,24	0,90	1,66
0,85	1,39	1,63	1,20	1,63	0,55	1,75	0,80	1,56
0,55	1,63	0,54	1,48	0,30	0,77	1,35	1,23	1,79
0,61	1,12	0,65	1,09	1,73	1,28	0,91	0,37	1,05
1,54	0,57	1,86	0,98	1,72	1,14	1,28	1,94	1,66
1,94	1,23	1,94	1,19	1,54	1,61	0,72	1,99	1,32

1,98	1,09	1,23	1,08	0,85	1,12	0,60	1,05	1,11
1,65	0,80	0,66	0,88	0,90	0,72	0,89	1,04	1,86
1,47	1,68	0,86	1,05	0,84	0,87	0,86	1,49	1,47
1,18	1,21	0,93	0,56	1,44	1,67	0,67	0,34	0,67
0,79	0,59	0,42	1,30	1,17	0,90	1,03	0,56	1,18
1,03	0,89	0,99	1,47	0,95	0,34	1,27	1,66	1,40
0,86	0,97	1,11	1,22	1,23	1,05	1,91	1,12	1,89
0,87	1,81	1,78	1,46	1,26	0,42	0,59	1,69	1,12
0,73	0,65	0,67	0,70	1,45	1,85	1,56	1,81	1,66
0,75	0,92	0,61	0,91	1,30	1,72	1,30	1,13	1,05
1,42	0,88	0,39	1,76	0,61	1,02	1,45	0,50	1,36
1,76	1,19	0,82	1,26	0,41	1,71	0,84	1,29	1,72
1,50	1,79	1,59	1,06	1,93	0,46	0,87	1,02	1,46
1,64	0,58	1,83	1,62	0,69	0,90	0,91	0,87	1,14
1,37	0,77	0,37	1,01	1,52	1,20	1,08	0,37	0,97
0,85	1,29	1,28	0,49	1,44	1,59	1,76	1,83	1,85
1,29	0,76	1,93	1,79	1,71	0,70	1,39	0,51	1,56
1,21	0,34	0,85	0,67	0,47	1,53	1,96	1,16	1,64
1,69	1,99	0,52	2,00	0,57	0,87	0,86	1,74	1,96
1,67	1,61	1,89	1,51	1,95	0,80	1,59	0,55	1,76

ToR-ORd - High-Risk Population - 72 Human Ventricular Cell Models

G_{Na}	G_{NaL}	G_{to}	G_{Kr}	G_{Ks}	G_{K1}	G_{NCX}	G_{NaK}	G_{CaL}
1,74	1,74	1,12	0,98	0,77	1,83	1,58	0,80	1,24
1,52	1,45	1,71	0,59	0,47	1,31	1,10	0,58	1,18
1,59	1,91	0,10	0,97	0,81	0,73	1,24	0,76	1,38
1,38	1,28	1,94	0,65	0,63	1,87	1,47	0,73	1,11
1,66	1,06	1,01	0,80	0,06	0,82	1,40	0,74	1,50
1,28	1,72	0,95	0,66	0,21	0,66	1,11	0,64	1,17
1,96	1,48	1,54	0,89	0,26	0,92	1,39	0,72	1,47
1,82	1,01	0,53	0,83	0,24	1,31	1,92	0,64	1,52
1,65	1,63	1,78	0,94	0,24	1,35	1,62	0,62	1,49
1,41	1,63	0,77	0,61	0,63	1,57	1,15	0,98	1,38
1,14	1,66	0,40	0,90	0,36	0,67	1,30	0,71	1,35
1,44	1,57	0,31	0,84	0,64	0,56	1,57	0,70	1,32
1,60	1,23	0,82	0,82	0,94	0,56	1,73	0,99	1,27
1,70	1,82	0,74	0,65	0,45	1,74	1,32	0,67	1,16
1,53	1,72	0,45	0,93	1,00	1,82	1,32	0,94	1,15
1,05	1,46	1,50	0,87	0,99	0,71	1,88	0,79	1,30
1,72	1,73	0,95	0,86	0,02	0,54	1,35	0,65	1,02
1,95	1,19	1,70	0,72	0,16	1,18	1,75	0,84	1,69
1,56	1,11	1,50	0,70	0,71	0,63	1,90	0,75	1,10
1,82	1,10	1,03	0,93	0,27	0,87	1,21	0,89	1,45
1,54	1,65	0,13	0,89	0,38	1,63	1,75	0,86	1,65
1,62	1,12	1,42	0,73	0,05	0,65	1,78	0,81	1,39
1,52	1,25	0,71	0,86	0,44	0,61	1,13	0,72	1,41
1,87	1,64	1,40	0,98	0,97	1,66	1,36	0,68	1,04
1,79	1,62	1,86	0,78	0,70	1,19	1,58	0,91	1,55
1,07	1,05	1,55	0,64	0,96	1,73	1,06	0,60	1,01
1,68	1,32	0,93	0,83	0,86	1,89	1,79	0,83	1,36
1,64	1,26	1,18	0,83	0,87	1,56	1,60	0,86	1,46
1,89	1,90	1,25	0,84	0,84	0,98	1,53	0,96	1,42
1,17	1,93	0,24	0,85	0,82	1,97	1,18	0,95	1,35
1,66	1,83	0,56	0,86	0,31	1,76	1,65	0,65	1,58
1,73	1,37	0,48	0,93	0,44	1,80	1,33	0,69	1,07
1,79	1,50	0,90	0,90	0,25	1,64	1,46	0,73	1,56
1,36	1,89	1,26	0,94	0,47	0,89	1,97	0,89	1,12
1,49	1,27	1,13	0,77	0,20	0,64	1,21	0,66	1,29
1,31	1,35	1,84	0,94	0,62	1,79	1,45	0,64	1,47
1,03	1,26	1,06	0,87	0,65	0,96	1,74	0,71	1,28
1,06	1,14	1,52	0,63	0,92	1,72	1,68	0,99	1,43
1,12	1,17	1,20	1,00	0,28	1,64	1,98	0,76	1,66
1,92	1,15	0,63	0,66	0,92	1,37	1,16	0,86	1,04
1,25	1,01	0,57	0,81	0,56	1,36	1,65	0,63	1,33
1,59	1,57	1,14	0,96	0,99	1,04	1,59	0,78	1,23
1,92	1,52	0,82	0,99	0,66	1,70	1,54	0,85	1,16
1,40	1,61	1,99	0,62	0,17	1,69	1,09	0,77	1,17

1,89	1,24	0,78	0,94	0,30	0,82	1,14	0,73	1,03
1,80	1,80	0,16	0,75	0,09	1,05	1,39	0,97	1,26
1,87	1,33	1,09	0,92	0,51	0,50	1,08	0,79	1,01
1,60	1,12	1,47	0,83	0,35	0,58	1,12	0,88	1,14
1,61	1,54	1,74	0,71	0,74	1,23	1,61	0,59	1,12
1,42	1,47	0,48	0,64	0,12	1,93	1,41	0,77	1,32
1,42	1,04	0,83	0,76	0,26	1,29	1,36	0,67	1,09
1,51	1,18	0,67	0,64	0,91	1,96	1,59	0,78	1,19
1,98	1,79	1,73	0,96	0,83	0,93	1,83	0,51	1,13
1,69	1,07	1,76	0,70	0,46	0,77	2,00	0,58	1,05
1,50	1,16	0,79	0,81	0,13	1,67	1,12	0,81	1,23
1,23	1,59	1,27	0,75	0,30	0,91	1,52	0,81	1,11
1,43	1,31	0,11	0,90	0,55	1,02	1,76	0,94	1,82
1,44	1,40	1,68	0,78	0,86	1,81	1,35	0,78	1,34
1,09	1,37	0,03	0,95	0,53	1,01	1,23	0,82	1,28
1,93	1,31	0,26	0,69	0,29	1,62	1,30	0,58	1,08
1,67	1,89	1,28	0,82	0,58	0,86	1,34	0,97	1,43
1,33	1,67	0,44	0,58	0,96	1,28	1,04	0,70	1,05
1,74	1,00	1,44	0,90	0,54	1,15	1,82	0,50	1,07
1,26	1,21	0,18	0,64	0,90	1,74	1,38	0,74	1,03
1,19	1,03	0,32	0,88	0,08	1,41	1,80	0,55	1,31
1,13	1,66	0,20	0,62	0,09	1,78	1,02	0,88	1,21
1,25	1,48	0,02	0,75	0,91	0,88	1,19	0,62	1,06
1,00	1,34	1,60	0,80	0,07	1,99	1,53	0,51	1,36
1,45	1,07	0,52	0,57	0,69	1,24	1,42	0,56	1,25
1,70	1,13	1,33	0,95	0,93	0,70	1,74	0,98	1,71
1,21	1,29	0,27	0,87	0,65	1,02	1,64	0,68	1,27
1,40	1,54	0,84	0,85	0,57	1,07	1,70	0,80	1,44

ORd2-CiPA - High-Risk Population - 92 Human Ventricular Cell Models

G_{Na}	G_{NaL}	G_{to}	G_{Kr}	G_{Ks}	G_{K1}	G_{NCX}	G_{NaK}	G_{CaL}
1,17	1,15	1,81	0,88	0,89	1,32	1,57	0,73	1,07
0,86	1,71	0,07	0,79	0,17	1,46	1,26	0,73	1,13
1,13	1,12	0,71	0,56	0,46	0,89	1,51	0,54	1,06
0,76	1,28	1,58	0,85	0,98	1,58	1,89	0,74	1,28
1,44	1,48	0,22	0,98	0,39	1,91	1,77	0,53	1,79
0,70	1,46	1,04	0,77	0,83	1,20	1,87	0,66	1,61
1,72	1,74	0,53	0,97	0,67	1,29	1,42	0,69	1,61
1,47	1,11	0,62	0,72	0,54	1,49	1,35	0,97	1,02
1,95	1,92	1,07	0,88	0,93	1,13	1,43	0,65	1,43
1,96	1,27	0,76	0,86	0,15	1,19	1,37	0,74	1,12
1,42	1,67	1,04	0,65	0,66	1,97	1,60	0,52	1,30
1,61	1,68	0,29	0,85	0,86	1,09	1,98	0,77	1,22
1,92	1,38	1,36	0,83	0,09	1,89	1,23	0,53	1,20
1,57	1,02	0,97	0,89	0,34	0,60	1,73	0,78	1,30
1,85	1,24	0,11	0,62	0,52	1,95	1,72	0,51	1,09
1,48	1,65	0,25	0,96	0,88	0,56	1,20	0,75	1,32
0,53	1,72	1,85	0,85	0,73	1,01	1,36	0,93	1,19
1,97	1,61	1,54	0,71	0,56	1,81	1,75	0,96	1,08
1,35	1,63	1,81	0,71	0,44	1,87	1,29	0,64	1,27
1,74	1,05	1,15	0,70	0,77	0,52	1,70	0,62	1,66
1,12	1,90	1,75	0,90	0,51	1,34	1,74	0,67	1,32
0,61	1,47	1,26	0,69	0,92	0,94	1,33	0,82	1,53
1,31	1,85	0,51	0,98	0,70	0,57	1,67	0,67	1,63
0,91	1,62	1,95	0,98	0,62	1,09	1,71	0,52	1,13
1,00	1,20	0,58	0,80	0,21	1,11	1,61	0,90	1,57
0,69	1,66	0,45	0,73	0,70	1,32	1,47	0,52	1,42
0,66	1,40	0,77	0,99	0,88	1,34	1,61	0,73	1,94
1,32	1,00	0,03	0,76	0,65	1,86	1,02	0,61	1,08
0,72	1,79	0,94	0,73	0,73	1,27	1,10	0,71	1,09
0,83	1,43	1,02	0,73	0,58	1,61	1,94	0,79	1,65
1,37	1,08	0,19	0,63	0,14	0,76	1,34	0,92	1,16
0,57	1,21	1,69	0,90	0,68	1,83	1,92	0,59	1,38
0,78	1,26	0,45	0,82	0,18	1,03	1,39	0,81	1,43
1,54	1,33	1,10	0,99	0,83	0,99	1,46	0,66	1,63
1,21	1,17	0,79	0,75	0,49	1,96	1,74	0,92	1,60
0,89	1,57	1,53	0,94	0,86	1,27	1,54	0,81	1,44
1,90	1,23	0,49	0,60	0,41	1,25	1,56	0,94	1,03
0,73	1,34	0,68	0,93	0,68	0,59	1,45	0,97	1,60
0,58	1,27	1,31	0,51	0,85	0,78	1,77	0,72	1,39
1,14	1,24	0,92	0,97	0,75	1,85	1,88	0,76	1,66
1,77	1,21	0,99	0,99	0,08	1,65	1,40	0,89	1,21
1,47	1,37	1,08	0,92	0,87	1,47	1,62	0,66	1,31
1,34	1,29	1,48	0,82	0,77	1,78	1,24	0,89	1,50
1,46	1,05	1,89	0,81	0,64	1,84	1,06	0,85	1,03

0,80	1,96	0,50	0,90	0,82	1,77	1,84	0,54	1,14
0,71	1,51	1,11	0,82	0,17	1,75	1,93	0,57	1,24
1,58	1,30	1,88	0,93	0,03	1,92	1,98	0,89	1,20
0,55	1,44	0,78	0,71	0,28	1,55	1,75	0,55	1,54
1,23	1,65	0,14	0,98	0,72	1,53	1,27	0,94	1,36
0,82	1,11	0,98	0,77	0,85	1,16	1,76	0,98	1,91
1,40	1,32	1,87	0,66	0,61	0,55	1,53	0,84	1,72
0,50	1,50	0,39	0,94	0,65	1,03	1,80	0,77	1,37
1,24	1,25	0,05	1,00	0,21	1,21	1,69	0,99	1,21
1,39	1,67	0,36	0,95	0,75	1,14	1,91	0,93	1,67
1,76	1,63	0,97	0,76	0,74	0,68	1,08	0,89	1,26
0,78	1,59	0,16	0,92	0,39	1,37	1,96	0,64	1,53
0,80	1,86	0,09	1,00	0,40	1,53	1,07	0,85	1,11
1,05	1,03	1,56	0,88	0,11	1,77	1,95	0,56	1,33
1,71	1,08	0,17	0,66	0,47	1,17	1,36	0,88	1,54
0,53	1,32	1,99	0,84	0,30	0,71	1,85	0,68	1,29
0,64	1,20	1,06	0,72	0,15	1,43	1,39	0,86	1,59
1,99	1,01	1,27	0,69	0,69	0,69	1,84	0,86	1,70
1,80	1,80	0,82	0,89	0,50	1,04	1,92	0,54	1,35
1,20	1,16	0,46	0,51	0,43	1,25	1,40	0,68	1,14
0,81	1,83	0,41	0,91	0,82	1,11	1,48	0,87	1,48
1,01	1,42	1,31	0,68	0,13	1,93	1,23	0,57	1,10
1,83	1,54	1,83	0,81	0,72	0,81	1,32	0,82	1,22
1,67	1,03	0,27	0,79	0,53	1,08	1,97	0,69	1,35
1,44	1,48	1,48	0,89	0,74	1,39	1,82	0,90	1,97
0,90	1,31	1,88	0,70	0,78	1,18	1,16	0,83	1,02
0,68	1,58	0,23	0,87	0,40	0,63	1,02	0,97	1,25
1,03	1,12	1,84	0,85	0,94	1,13	1,38	0,74	1,28
1,98	1,22	0,00	0,89	0,42	1,22	1,88	0,65	1,50
1,29	1,04	0,35	0,66	0,50	0,95	1,68	0,70	1,07
1,08	1,34	1,24	0,96	0,19	0,73	1,78	0,51	1,04
0,52	1,35	1,72	0,95	0,97	1,30	1,19	0,57	1,12
1,90	1,41	1,49	0,73	0,37	1,88	1,56	0,95	1,23
1,62	1,81	1,11	0,78	0,60	1,86	1,09	0,81	1,05
1,37	1,46	0,28	0,81	0,06	1,95	1,82	0,79	1,17
0,96	1,26	0,72	0,96	0,63	0,83	2,00	0,90	1,78
1,55	1,72	1,75	0,78	0,79	1,80	1,90	0,64	1,72
1,95	1,81	1,96	0,83	0,89	0,67	1,71	0,78	1,00
1,14	1,66	0,85	0,93	0,61	0,74	1,42	0,55	1,49
0,92	1,99	0,33	0,91	0,80	0,58	1,17	0,60	1,01
0,73	1,13	0,81	0,91	0,90	0,82	1,34	0,88	1,41
1,30	1,01	0,40	0,92	0,10	1,02	1,27	0,85	1,10
1,49	1,19	0,90	0,68	0,27	0,97	1,21	0,87	1,39
0,88	1,57	1,44	0,99	0,81	0,81	1,25	0,79	1,19
0,62	1,18	0,94	0,76	0,38	1,50	1,17	0,99	1,29
0,89	1,50	1,34	0,69	0,76	1,07	1,96	0,74	1,36
0,98	1,09	0,91	0,86	0,20	1,69	1,47	0,82	1,40

0,99	1,78	1,70	0,86	0,91	1,18	1,59	0,75	1,15

ORd - High-Risk Population - 118 Human Ventricular Cell Models

G_{Na}	G_{NaL}	G_{to}	G_{Kr}	G_{Ks}	G_{K1}	G_{NCX}	G_{NaK}	G_{CaL}
1,55	1,86	0,76	0,71	0,61	1,21	1,20	0,51	1,07
1,31	1,18	0,51	0,74	0,13	1,50	1,35	0,63	1,09
1,99	1,12	1,46	0,63	0,16	1,02	1,99	0,77	1,63
1,70	1,07	0,79	1,00	0,75	1,19	1,44	0,94	1,89
0,99	1,09	1,18	0,63	0,78	0,89	1,33	0,85	1,32
1,30	1,57	1,25	0,98	0,92	1,08	1,69	0,70	1,62
1,94	1,41	1,60	0,80	0,64	1,56	1,42	0,76	1,50
0,98	1,84	1,93	1,00	0,10	1,99	1,86	0,88	1,25
0,76	1,59	0,91	0,67	0,95	1,85	1,12	0,58	1,07
1,63	1,14	0,69	0,94	0,89	1,26	1,43	0,71	1,29
1,01	1,32	0,87	0,68	0,64	1,48	1,57	0,95	1,65
0,51	1,82	1,25	0,71	0,17	1,51	1,97	0,69	1,69
1,79	1,60	1,56	0,66	0,21	1,59	1,50	0,96	1,77
1,40	1,62	1,44	0,90	0,12	0,56	1,95	0,77	1,73
1,38	1,78	1,94	0,81	0,44	0,73	1,63	0,68	1,88
1,00	1,53	0,37	0,77	0,91	1,15	1,23	0,84	1,78
1,66	1,21	1,71	0,81	0,00	1,55	1,15	0,79	1,59
1,67	1,01	1,68	0,73	0,33	0,85	1,07	0,79	1,50
1,15	1,96	0,96	0,62	0,70	1,43	1,30	0,96	1,36
1,93	1,56	0,89	0,82	0,71	1,89	1,56	0,76	1,64
1,36	1,03	0,25	0,97	0,91	1,08	1,48	0,98	1,96
1,63	1,88	0,70	0,79	0,83	1,70	1,97	0,75	1,97
0,91	1,89	0,61	0,53	0,87	1,34	1,65	0,89	1,55
0,77	1,02	0,25	0,89	0,23	0,56	1,75	0,80	1,05
1,26	1,86	0,52	0,83	0,77	1,66	1,58	0,63	1,45
1,98	1,97	0,34	0,71	0,39	1,94	1,36	0,95	1,87
1,62	1,75	0,85	0,72	0,88	0,78	1,61	0,61	1,53
0,66	1,01	1,30	0,98	0,62	0,85	1,78	0,70	1,02
0,81	1,33	0,58	0,86	0,26	0,95	1,64	0,95	1,46
1,57	1,03	1,78	0,91	0,94	0,60	1,45	0,53	1,24
1,60	1,77	1,30	0,72	0,36	1,23	1,20	0,80	1,36
1,09	1,37	0,33	0,69	0,83	1,78	1,29	0,86	1,82
0,97	1,94	1,84	0,86	0,50	1,24	1,61	0,71	1,72
1,42	1,55	1,14	0,86	0,27	1,33	1,94	0,62	1,35
1,86	1,60	1,28	0,60	0,93	1,57	1,54	0,65	1,40
1,65	1,99	1,01	0,78	0,20	1,67	1,98	0,98	1,83
0,85	1,24	0,43	0,81	0,73	0,72	1,40	0,79	1,40
1,58	1,78	1,62	0,97	0,61	1,70	1,32	0,99	1,37
0,72	1,68	0,76	0,98	0,58	1,03	1,63	0,60	1,16
0,87	1,42	0,53	0,76	0,74	1,13	1,67	0,67	1,67
1,19	1,95	1,23	0,69	0,26	1,38	1,89	0,85	1,59
0,88	1,76	0,41	0,80	0,54	1,64	1,54	0,92	1,09
0,63	1,48	1,97	0,56	0,15	1,76	1,17	0,68	1,02
1,35	1,59	0,18	0,75	0,56	0,90	1,13	0,68	1,26

1,73	1,81	1,73	0,82	0,31	1,61	1,84	0,63	1,63
0,86	1,55	0,65	0,90	0,72	1,52	1,96	0,67	1,11
1,85	1,10	0,93	0,88	0,07	1,09	1,22	0,58	1,17
0,75	1,52	0,58	0,61	0,81	0,66	1,36	0,70	1,13
1,80	1,71	0,04	0,81	0,89	1,91	1,49	0,67	1,49
0,52	1,11	1,74	0,57	0,37	1,27	1,74	0,79	1,26
0,97	1,72	1,77	0,99	0,80	1,35	1,86	0,94	1,22
1,97	2,00	1,63	0,76	0,96	1,32	1,32	0,91	1,72
1,61	1,40	0,08	0,84	0,48	0,71	1,37	0,62	1,80
0,83	1,58	1,81	0,82	0,35	1,53	1,90	0,51	1,51
1,03	1,70	1,83	0,78	0,07	1,81	1,38	0,78	1,51
1,93	1,04	1,75	0,75	0,11	0,80	1,00	0,95	1,34
1,65	1,91	1,55	0,92	0,44	1,04	1,76	0,61	1,25
0,88	1,61	0,82	0,73	0,82	1,76	1,19	0,51	1,21
1,87	1,16	1,43	0,90	0,66	0,80	1,53	0,75	1,20
1,19	1,25	0,30	0,67	0,82	1,86	1,41	0,96	1,85
0,95	1,50	0,02	0,97	0,40	1,92	1,72	0,89	1,57
1,89	1,56	1,47	0,73	0,70	0,53	1,82	0,73	1,67
0,66	1,42	0,47	0,66	0,45	0,59	1,26	0,88	1,24
1,49	1,17	0,46	0,80	0,85	1,96	1,71	0,73	1,31
1,16	1,34	0,44	0,65	0,42	1,93	1,07	0,65	1,04
1,84	1,23	1,03	0,61	0,34	1,39	1,11	0,64	1,15
0,82	1,24	0,18	0,60	0,66	0,70	1,77	0,80	1,30
1,45	1,83	1,68	0,96	0,16	1,43	1,15	0,85	1,61
1,34	1,11	1,36	0,96	0,59	0,59	1,28	0,98	1,22
0,56	1,84	0,88	0,91	0,76	1,49	1,89	0,59	1,18
1,32	1,98	1,12	0,72	0,99	1,05	1,66	0,60	1,08
1,04	1,46	1,03	0,95	0,47	1,68	1,27	0,56	1,27
0,68	1,43	1,88	0,62	0,80	1,83	1,92	0,87	1,82
0,62	1,91	0,08	0,88	0,22	1,40	1,71	0,87	1,55
1,20	1,63	1,48	0,55	0,85	1,12	1,53	0,90	1,41
1,08	1,22	0,15	0,95	0,90	1,21	1,27	0,81	1,10
0,93	1,12	1,14	0,66	0,98	1,36	1,35	0,72	1,44
0,57	1,43	0,92	0,74	0,59	0,76	1,98	0,82	1,49
1,49	1,36	0,28	0,93	0,22	0,66	1,50	1,00	1,75
1,01	1,35	0,10	0,96	0,60	1,80	1,18	0,87	1,30
1,23	1,54	0,22	0,95	0,57	1,28	1,52	0,88	1,66
1,09	1,20	1,59	0,89	0,20	0,50	1,70	0,89	1,52
1,41	1,08	1,78	0,82	0,84	0,82	1,68	0,55	1,21
1,59	1,79	0,59	0,84	0,28	1,30	1,03	0,83	1,61
1,04	1,85	0,35	0,92	0,19	1,96	1,64	0,76	1,95
0,93	1,82	1,76	0,59	0,87	1,58	1,79	0,73	1,00
1,08	1,50	1,57	0,70	0,74	0,83	1,39	0,60	1,23
0,65	1,39	0,23	0,92	0,60	0,88	1,22	0,90	1,79
1,70	1,26	1,12	0,86	0,57	0,67	1,72	0,69	1,48
0,77	1,74	1,49	0,96	0,39	1,25	1,06	0,74	1,31
1,24	1,31	1,98	0,90	0,34	0,92	1,81	0,91	1,39

1,77	1,64	1,43	0,75	0,52	1,81	1,21	0,66	1,20
0,94	1,71	1,39	0,61	0,65	1,20	1,16	0,84	1,42
1,06	1,76	1,18	0,93	0,96	1,77	1,88	0,59	1,38
1,43	1,90	0,37	0,88	0,36	0,82	1,09	0,53	1,11
1,39	1,25	0,80	0,52	0,81	1,66	1,68	0,67	1,78
1,66	1,69	0,11	0,95	0,08	1,95	1,60	0,66	1,60
1,52	1,06	0,54	0,97	0,27	0,96	1,28	0,59	1,12
1,74	1,51	0,84	0,66	0,37	1,41	1,73	0,86	1,37
1,24	1,53	1,87	0,77	0,04	0,93	1,24	0,94	1,62
1,21	1,85	0,48	0,80	0,53	1,69	1,59	0,55	1,19
1,17	1,06	1,95	0,53	0,79	1,07	1,08	0,66	1,12
1,22	1,83	0,68	0,85	0,47	1,00	1,56	0,50	1,39
0,90	1,57	1,23	0,71	0,41	0,69	1,23	0,90	1,58
1,33	1,02	0,26	0,57	1,00	0,94	1,87	0,97	1,45
1,38	1,93	0,00	0,75	0,69	0,75	1,33	0,82	1,68
1,11	1,26	0,53	0,61	0,29	1,52	1,34	0,97	1,27
1,72	1,64	1,05	0,84	0,02	1,17	1,47	0,99	1,47
1,71	1,29	1,00	0,63	0,52	1,37	1,14	0,75	1,57
1,47	1,29	1,71	0,65	0,12	1,72	1,45	0,57	1,19
1,28	1,48	0,15	0,94	0,40	1,58	1,25	0,87	1,38
0,91	1,17	1,21	0,99	0,15	1,30	1,34	0,84	1,56
0,62	1,79	1,04	0,91	0,68	1,18	1,76	0,58	1,03
1,95	1,47	0,66	0,77	0,30	1,54	1,66	0,97	1,65
0,73	1,51	0,22	0,83	0,29	1,11	1,52	0,81	1,14
1,83	1,62	0,39	0,64	0,09	1,74	1,48	0,77	1,76
1,78	1,05	1,56	0,64	0,54	1,27	1,43	0,56	1,08
1,02	1,13	0,57	0,76	0,93	1,84	1,96	0,84	1,94
