# Supplementary material for: Applying the CiPA approach to evaluate cardiac proarrhythmia risk of some antimalarials used off‐label in the first wave of COVID‐19
Source: Clin Transl Sci. 2021 Apr 9;14(3):1133–46. doi: 10.1111/cts.13011 (PMC8014548; doi:10.1111/cts.13011)
Supplement: Supplementary file 3 — Table S2 [file CTS-14-1133-s001.pdf]

Table S2: Effects of CLQ, OH-CLQ, AZI, and ERT, alone and in combination, on a selection of cardiovascular biomarkers, in control and high-risk populations of virtual human cardiomyocytes (Virtual Assay®), using the 3 different computational models (ToRd, ORd-CiPA, and ORd) and the 2 ion current datasets.

**ToR-ORd: Control Population**								
IC_{50} - Dataset #1								
	**CLQ**	**OH-CLQ**	**AZI**	**ERT**	**CLQ + AZI**	**CLQ + ERT**	**OH-CLQ + AZI**	**OH-CLQ + ERT**
**APD_{90}**	↑ 1 µM (33%)	↑ 5 µM (25%)	↑ 1 mM (37%)	↑ 300 µM (29%)	↑ 1 µM (34%)	↑ 1 µM (33%)	↑ 5 µM (25%)	↑ 5 µM (26%)
**Tri_{90-40}**	↑ 0.5 µM (28%)	↑ 3 µM (25%)	↑ 1 mM (26%)	↑ 300 µM (45%)	↑ 0.5 µM (29%)	↑ 0.5 µM (29%)	↑ 3 µM (25%)	↑ 3 µM (26%)
**dV/dt_{MAX}**	↓ 10 µM (-29%)	↓ 30 µM (-29%)	↓ 1 mM (-33%)	*No change*	↓ 10 µM (-31%)	↓ 10 µM (-30%)	↓ 30 µM (-31%)	↓ 30 µM (-29%)
**CTD_{90}**	↑ 10 µM (20%)	↑ 30 µM (24%)	*No change*	*No change*	↑ 10 µM (20%)	↑ 10 µM (20%)	↑ 30 µM (24%)	↑ 30 µM (25%)
**CT_{peak}**	↓ 10 µM (-28%)	↓ 30 µM (-41%)	↓ 1 mM (-35%)	*No change*	↓ 10 µM (-28%)	↓ 10 µM (-28%)	↓ 30 µM (-41%)	↓ 30 µM (-41%)
**EMw**	↓ 0.5 µM (-29%)	↓ 3 µM (-24%)	↓ 300 µM (-24%)	↓ 300 µM (-49%)	↓ 0.5 µM (-29%)	↓ 0.5 µM (-29%)	↓ 3 µM (-25%)	↓ 3 µM (-25%)
IC_{50} - Dataset #2								
	**CLQ**	**OH-CLQ**	**AZI**	**ERT**	**CLQ + AZI**	**CLQ + ERT**	**OH-CLQ + AZI**	**OH-CLQ + ERT**
**APD_{90}**	↑ 1 µM (29%)	↑ 3 µM (30%)	↑ 1 mM (68%)	↑ 100 µM (27%)	↑ 1 µM (29%)	↑ 1 µM (29%)	↑ 3 µM (30%)	↑ 3 µM (31%)
**Tri_{90-40}**	↑ 0.5 µM (23%)	↑ 3 µM (38%)	↑ 1 mM (41%)	↑ 100 µM (42%)	↑ 0.5 µM (23%)	↑ 0.5 µM (23%)	↑ 3 µM (38%)	↑ 3 µM (39%)
**dV/dt_{MAX}**	↓ 5 µM (-20%)	↓ 10 µM (-26%)	↓ 1 mM (-54%)	*No change*	↓ 5 µM (-20%)	↓ 5 µM (-20%)	↓ 10 µM (-26%)	↓ 10 µM (-83%)
**CTD_{90}**	↑ 5 µM (24%)	↑ 10 µM (27%)	*No change*	*No change*	↑ 5 µM (24%)	↑ 5 µM (25%)	↑ 10 µM (27%)	↑ 10 µM (27%)
**CT_{peak}**	↓ 3 µM (-24%)	↓ 3 µM (-23%)	↓ 1 mM (-66%)	*No change*	↓ 3 µM (-24%)	↓ 3 µM (-24%)	↓ 3 µM (-23%)	↓ 3 µM (-23%)
**EMw**	↓ 1 µM (-32%)	↓ 3 µM (-20%)	*No change*	↓ 100 µM (-45%)	↓ 1 µM (-32%)	↓ 1 µM (-32%)	↓ 3 µM (-20%)	↓ 3 µM (-21%)

ToR-ORd: High-Risk Population

IC 50 - Dataset #1

	CLQ	OH-CLQ	AZI	ERT	CLQ + AZI	CLQ + ERT	OH-CLQ + AZI	OH-CLQ + ERT
**APD_{90}**	↑ 1 µM (35%)	↑ 5 µM (24%)	↑ 1 mM (34%)	↑ 300 µM (30%)	↑ 1 µM (35%)	↑ 1 µM (35%)	↑ 5 µM (24%)	↑ 5 µM (24%)
**Tri_{90-40}**	↑ 0.5 µM (31%)	↑ 3 µM (26%)	*No change*	↑ 300 µM (48%)	↑ 0.5 µM (31%)	↑ 0.5 µM (32%)	↑ 3 µM (26%)	↑ 3 µM (27%)
**dV/dt_{MAX}**	↓ 10 µM (-46%)	↓ 30 µM (-28%)	↓ 1 mM (-32%)	*No change*	↓ 10 µM (-45%)	↓ 10 µM (-47%)	↓ 30 µM (-30%)	↓ 30 µM (-27%)
**CTD_{90}**	↑ 10 µM (21%)	↑ 30 µM (28%)	↑ 1 mM (26%)	*No change*	↑ 10 µM (23%)	↑ 10 µM (21%)	↑ 30 µM (28%)	↑ 30 µM (28%)
**CT_{peak}**	↓ 5 µM (-21%)	↓ 10 µM (-28%)	↓ 1 mM (-42%)	*No change*	↓ 5 µM (-20%)	↓ 5 µM (-21%)	↓ 10 µM (-28%)	↓ 10 µM (-28%)
**EMw**	↓ 0.5 µM (-43%)	↓ 1 µM (-20%)	↓ 300 µM (-41%)	↓ 100 µM (-32%)	↓ 0.5 µM (-43%)	↓ 0.5 µM (-43%)	↓ 1 µM (-20%)	↓ 1 µM (-20%)

IC 50 - Dataset #2

	CLQ	OH-CLQ	AZI	ERT	CLQ + AZI	CLQ + ERT	OH-CLQ + AZI	OH-CLQ + ERT
**APD_{90}**	↑ 1 µM (27%)	↑ 3 µM (27%)	↑ 1 mM (48%)	↑ 100 µM (28%)	↑ 1 µM (28%)	↑ 1 µM (27%)	↑ 3 µM (27%)	↑ 3 µM (27%)
**Tri_{90-40}**	↑ 0.5 µM (24%)	↑ 3 µM (32%)	*No change*	↑ 100 µM (45%)	↑ 0.5 µM (24%)	↑ 0.5 µM (24%)	↑ 3 µM (32%)	↑ 3 µM (32%)
**dV/dt_{MAX}**	↓ 10 µM (-28%)	↓ 10 µM (-25%)	↓ 1 mM (-51%)	*No change*	↓ 10 µM (-29%)	↓ 10 µM (-29%)	↓ 10 µM (-25%)	↓ 10 µM (-25%)
**CTD_{90}**	↑ 3 µM (24%)	↑ 5 µM (23%)	↑ 1 mM (48%)	*No change*	↑ 3 µM (24%)	↑ 3 µM (25%)	↑ 3 µM (20%)	↑ 3 µM (20%)
**CT_{peak}**	↓ 1 µM (-22%)	↓ 3 µM (-32%)	↓ 1 mM (-68%)	*No change*	↓ 1 µM (-22%)	↓ 1 µM (-22%)	↓ 3 µM (-32%)	↓ 3 µM (-32%)
**EMw**	↓ 0.5 µM (-24%)	↑ 30 µM (82%)	*No change*	↓ 30 µM (-24%)	↓ 0.5 µM (-24%)	↓ 0.5 µM (-25%)	↑ 30 µM (82%)	↑ 30 µM (64%)

ORd-CiPA: Control Population								
IC_{50} - Dataset #1								
	**CLQ**	**OH-CLQ**	**AZI**	**ERT**	**CLQ + AZI**	**CLQ + ERT**	**OH-CLQ + AZI**	**OH-CLQ + ERT**
**APD_{90}**	↑ 0.5 µM (20%)	↑ 3 µM (21%)	↑ 1 mM (42%)	↑ 300 µM (31%)	↑ 0.5 µM (20%)	↑ 0.5 µM (20%)	↑ 3 µM (21%)	↑ 3 µM (21%)
**Tri_{90-40}**	↑ 0.5 µM (31%)	↑ 3 µM (34%)	↑ 300 µM (24%)	↑ 300 µM (48%)	↑ 0.5 µM (31%)	↑ 0.5 µM (31%)	↑ 3 µM (34%)	↑ 3 µM (34%)
**dV/dt_{MAX}**	↓ 30 µM (-68%)	↓ 300 µM (-67%)	*No change*	*No change*	↓ 30 µM (-68%)	↓ 30 µM (-68%)	↓ 300 µM (-67%)	↓ 300 µM (-67%)
**CTD_{90}**	↑ 10 µM (26%)	↑ 30 µM (20%)	*No change*	*No change*	↑ 10 µM (25%)	↑ 10 µM (26%)	↑ 30 µM (21%)	↑ 30 µM (21%)
**CT_{peak}**	↓ 30 µM (-29%)	↓ 30 µM (-31%)	↓ 1 mM (-28%)	↓ 1 mM (-20%)	↓ 30 µM (-30%)	↓ 30 µM (-29%)	↓ 30 µM (-31%)	↓ 30 µM (-31%)
**EMw**	↓ 1 µM (-42%)	↓ 5 µM (-21%)	No change	↓ 300 µM (-31%)	↓ 1 µM (-34%)	↓ 1 µM (-34%)	↓ 5 µM (-22%)	↓ 5 µM (-22%)
IC_{50} - Dataset #2								
	**CLQ**	**OH-CLQ**	**AZI**	**ERT**	**CLQ + AZI**	**CLQ + ERT**	**OH-CLQ + AZI**	**OH-CLQ + ERT**
**APD_{90}**	↑ 1 µM (32%)	↑ 3 µM (34%)	↑ 1 mM (88%)	↑ 100 µM (29%)	↑ 1 µM (32%)	↑ 1 µM (32%)	↑ 3 µM (34%)	↑ 3 µM (35%)
**Tri_{90-40}**	↑ 0.5 µM (30%)	↑ 1 µM (25%)	↑ 300 µM (28%)	↑ 100 µM (44%)	↑ 0.5 µM (30%)	↑ 0.5 µM (30%)	↑ 1 µM (25%)	↑ 1 µM (25%)
**dV/dt_{MAX}**	*No change*	*No change*	*No change*	*No change*	*No change*	*No change*	*No change*	*No change*
**CTD_{90}**	↑ 5 µM (20%)	↑ 10 µM (22%)	↑ 1 mM (43%)	*No change*	↑ 5 µM (20%)	↑ 5 µM (20%)	↑ 10 µM (22%)	↑ 10 µM (22%)
**CT_{peak}**	↓ 5 µM (-25%)	↓ 5 µM (-20%)	↓ 1 mM (-60%)	↓ 300 µM (-20%)	↓ 5 µM (-25%)	↓ 5 µM (-25%)	↓ 5 µM (-20%)	↓ 5 µM (-20%)
**EMw**	↓ 1 µM (-23%)	↓ 5 µM (-20%)	No change	↓ 100 µM (-29%)	↓ 1 µM (-23%)	↓ 1 µM (-23%)	↓ 5 µM (-20%)	↓ 5 µM (-21%)

ORd-CiPA: High-Risk Population								
IC $_{50}$ - Dataset #1								
	**CLQ**	**OH-CLQ**	**AZI**	**ERT**	**CLQ + AZI**	**CLQ + ERT**	**OH-CLQ + AZI**	**OH-CLQ + ERT**
**APD_{90}**	↑ 0.5 µM (20%)	↑ 3 µM (21%)	↑ 1 mM (43%)	↑ 300 µM (32%)	↑ 0.5 µM (20%)	↑ 0.5 µM (20%)	↑ 3 µM (21%)	↑ 3 µM (21%)
**Tri_{90-40}**	↑ 0.5 µM (31%)	↑ 3 µM (33%)	↑ 300 µM (24%)	↑ 300 µM (50%)	↑ 0.5 µM (31%)	↑ 0.5 µM (32%)	↑ 3 µM (33%)	↑ 3 µM (33%)
**dV/dt_{MAX}**	*No change*	*No change*	*No change*	*No change*	*No change*	*No change*	*No change*	*No change*
**CTD_{90}**	↑ 3 µM (30%)	↑ 30 µM (37%)	↑ 1 mM (26%)	↑ 1 mM (23%)	↑ 3 µM (30%)	↑ 3 µM (29%)	↑ 30 µM (38%)	↑ 30 µM (39%)
**CT_{peak}**	*No change*	↓ 30 µM (-44%)	↓ 1 mM (-35%)	*No change*	*No change*	*No change*	↓ 30 µM (-44%)	↓ 30 µM (-46%)
**EMw**	↓ 0.5 µM (-29%)	↓ 3 µM (-24%)	↓ 300 µM (-21%)	↓ 100 µM (-20%)	↓ 0.5 µM (-29%)	↓ 0.5 µM (-29%)	↓ 3 µM (-24%)	↓ 3 µM (-24%)
IC $_{50}$ - Dataset #2								
	**CLQ**	**OH-CLQ**	**AZI**	**ERT**	**CLQ + AZI**	**CLQ + ERT**	**OH-CLQ + AZI**	**OH-CLQ + ERT**
**APD_{90}**	↑ 1 µM (33%)	↑ 3 µM (34%)	↑ 1 mM (87%)	↑ 100 µM (30%)	↑ 1 µM (33%)	↑ 1 µM (33%)	↑ 3 µM (34%)	↑ 3 µM (35%)
**Tri_{90-40}**	↑ 0.5 µM (29%)	↑ 1 µM (25%)	↑ 300 µM (28%)	↑ 100 µM (46%)	↑ 0.5 µM (29%)	↑ 0.5 µM (30%)	↑ 1 µM (25%)	↑ 1 µM (25%)
**dV/dt_{MAX}**	*No change*	*No change*	*No change*	*No change*	*No change*	*No change*	*No change*	*No change*
**CTD_{90}**	↑ 3 µM (29%)	↑ 5 µM (24%)	↑ 1 mM (56%)	↑ 300 µM (23%)	↑ 3 µM (29%)	↑ 3 µM (30%)	↑ 5 µM (24%)	↑ 5 µM (25%)
**CT_{peak}**	↓ 3 µM (-29%)	↓ 5 µM (-29%)	↓ 1 mM (-66%)	↓ 1 mM (-23%)	↓ 3 µM (-29%)	↓ 3 µM (-29%)	↓ 5 µM (-29%)	↓ 5 µM (-29%)
**EMw**	↓ 1 µM (-29%)	↑ 100 µM (22%)	No change	↓ 100 µM (-44%)	↓ 1 µM (-29%)	↓ 1 µM (-29%)	↑ 100 µM (22%)	↑ 300 µM (38%)

ORd: Control Population								
IC $_{50}$ - Dataset #1								
	**CLQ**	**OH-CLQ**	**AZI**	**ERT**	**CLQ + AZI**	**CLQ + ERT**	**OH-CLQ + AZI**	**OH-CLQ + ERT**
**APD_{90}**	↑ 0.5 µM (24%)	↑ 3 µM (26%)	↑ 1 mM (51%)	↑ 300 µM (34%)	↑ 0.5 µM (24%)	↑ 0.5 µM (24%)	↑ 3 µM (26%)	↑ 3 µM (26%)
**Tri_{90-40}**	↑ 0.5 µM (33%)	↑ 3 µM (35%)	↑ 300 µM (21%)	↑ 300 µM (50%)	↑ 0.5 µM (33%)	↑ 0.5 µM (33%)	↑ 3 µM (35%)	↑ 3 µM (35%)
**dV/dt_{MAX}**	↓ 30 µM (-69%)	↓ 30 µM (-39%)	↓ 300 µM (-22%)	*No change*	↓ 30 µM (-69%)	↓ 30 µM (-69%)	↓ 10 µM (-20%)	↓ 30 µM (-39%)
**CTD_{90}**	↑ 10 µM (24%)	↑ 100 µM (28%)	*No change*	*No change*	↑ 10 µM (24%)	↑ 10 µM (25%)	↑ 100 µM (27%)	↑ 100 µM (30%)
**CT_{peak}**	↓ 30 µM (-45%)	↓ 100 µM (-38%)	*No change*	*No change*	↓ 30 µM (-50%)	↓ 30 µM (-48%)	↓ 100 µM (-36%)	↓ 100 µM (-37%)
**EMw**	↓ 0.5 µM (-23%)	↓ 3 µM (-24%)	↓ 300 µM (-25%)	↓ 300 µM (-33%)	↓ 0.5 µM (-23%)	↓ 0.5 µM (-23%)	↓ 3 µM (-24%)	↓ 3 µM (-24%)
IC $_{50}$ - Dataset #2								
	**CLQ**	**OH-CLQ**	**AZI**	**ERT**	**CLQ + AZI**	**CLQ + ERT**	**OH-CLQ + AZI**	**OH-CLQ + ERT**
**APD_{90}**	↑ 0.5 µM (23%)	↑ 3 µM (42%)	↑ 300 µM (21%)	↑ 100 µM (32%)	↑ 0.5 µM (23%)	↑ 0.5 µM (23%)	↑ 3 µM (42%)	↑ 3 µM (43%)
**Tri_{90-40}**	↑ 0.5 µM (31%)	↑ 1 µM (25%)	↑ 300 µM (24%)	↑ 100 µM (46%)	↑ 0.5 µM (31%)	↑ 0.5 µM (31%)	↑ 1 µM (25%)	↑ 1 µM (25%)
**dV/dt_{MAX}**	↓ 3 µM (-20%)	↓ 5 µM (-24%)	↓ 300 µM (-26%)	*No change*	↓ 3 µM (-20%)	↓ 3 µM (-20%)	↓ 5 µM (-24%)	↓ 5 µM (-24%)
**CTD_{90}**	↑ 10 µM (24%)	↑ 30 µM (25%)	↑ 1 mM (29%)	↑ 1 mM (24%)	↑ 10 µM (24%)	↑ 10 µM (25%)	↑ 30 µM (24%)	↑ 30 µM (26%)
**CT_{peak}**	↓ 10 µM (-21%)	↓ 30 µM (-30%)	↓ 1 mM (-43%)	*No change*	↓ 10 µM (-21%)	↓ 10 µM (-22%)	↓ 30 µM (-30%)	↓ 30 µM (-30%)
**EMw**	↓ 0.5 µM (-20%)	↓ 3 µM (-30%)	↓ 300 µM (-21%)	↓ 100 µM (-32%)	↓ 0.5 µM (-20%)	↓ 0.5 µM (-20%)	↓ 3 µM (-30%)	↓ 3 µM (-31%)

ORd: High-Risk Population

IC_{50} - Dataset #1

	CLQ	OH-CLQ	AZI	ERT	CLQ + AZI	CLQ + ERT	OH-CLQ + AZI	OH-CLQ + ERT
**APD_{90}**	↑ 0.5 µM (22%)	↑ 3 µM (24%)	↑ 1 mM (50%)	↑ 300 µM (34%)	↑ 0.5 µM (22%)	↑ 0.5 µM (22%)	↑ 3 µM (24%)	↑ 3 µM (24%)
**$Tri_{90\text{-}40}$**	↑ 0.5 µM (36%)	↑ 3 µM (37%)	↑ 300 µM (22%)	↑ 300 µM (57%)	↑ 0.5 µM (36%)	↑ 0.5 µM (36%)	↑ 3 µM (37%)	↑ 3 µM (37%)
**dV/dt_{MAX}**	*No change*	↓ 30 µM (-39%)	↓ 300 µM (-22%)	*No change*	*No change*	*No change*	↓ 10 µM (-20%)	↓ 30 µM (-39%)
**CTD_{90}**	↑ 3 µM (35%)	↑ 30 µM (32%)	*No change*	↑ 1 mM (28%)	↑ 3 µM (34%)	↑ 3 µM (35%)	↑ 30 µM (32%)	↑ 30 µM (33%)
**CT_{peak}**	↓ 5 µM (-23%)	↓ 30 µM (-26%)	*No change*	*No change*	↓ 5 µM (-23%)	↓ 5 µM (-24%)	↓ 30 µM (-27%)	↓ 30 µM (-27%)
**EMw**	↓ 0.5 µM (-28%)	↓ 3 µM (-28%)	↓ 300 µM (-36%)	↓ 300 µM (-42%)	↓ 0.5 µM (-28%)	↓ 0.5 µM (-28%)	↓ 3 µM (-29%)	↓ 3 µM (-29%)

IC_{50} - Dataset #2

	CLQ	OH-CLQ	AZI	ERT	CLQ + AZI	CLQ + ERT	OH-CLQ + AZI	OH-CLQ + ERT
**APD_{90}**	↑ 0.5 µM (21%)	↑ 3 µM (41%)	↑ 300 µM (20%)	↑ 100 µM (32%)	↑ 0.5 µM (21%)	↑ 0.5 µM (21%)	↑ 3 µM (41%)	↑ 3 µM (41%)
**$Tri_{90\text{-}40}$**	↑ 0.5 µM (33%)	↑ 1 µM (26%)	↑ 300 µM (25%)	↑ 100 µM (53%)	↑ 0.5 µM (33%)	↑ 0.5 µM (33%)	↑ 1 µM (26%)	↑ 1 µM (26%)
**dV/dt_{MAX}**	↓ 3 µM (-21%)	↓ 5 µM (-24%)	↓ 300 µM (-25%)	*No change*	↓ 3 µM (-21%)	↓ 3 µM (-21%)	↓ 5 µM (-24%)	↓ 5 µM (-24%)
**CTD_{90}**	↑ 3 µM (30%)	↑ 5 µM (20%)	↑ 1 mM (47%)	↑ 300 µM (27%)	↑ 3 µM (30%)	↑ 3 µM (31%)	↑ 5 µM (20%)	↑ 5 µM (20%)
**CT_{peak}**	↓ 3 µM (-20%)	↓ 5 µM (-21%)	↓ 1 mM (-55%)	*No change*	↓ 3 µM (-20%)	↓ 3 µM (-21%)	↓ 5 µM (-21%)	↓ 5 µM (-22%)
**EMw**	↓ 0.5 µM (-22%)	↓ 3 µM (-34%)	↓ 300 µM (-30%)	↓ 100 µM (-40%)	↓ 0.5 µM (-22%)	↓ 0.5 µM (-22%)	↓ 3 µM (-34%)	↓ 3 µM (-35%)

Tables show the lowest concentration with effect, i.e. at which changes higher than 20% compared to control conditions were observed.
% change observed at this concentration is indicated in brackets (median values in the *in silico* populations were considered).
See main text for AP and CT biomarker descriptions.
