# Supplementary material for: Applying the CiPA approach to evaluate cardiac proarrhythmia risk of some antimalarials used off‐label in the first wave of COVID‐19
Source: Clin Transl Sci. 2021 Apr 9;14(3):1133–46. doi: 10.1111/cts.13011 (PMC8014548; doi:10.1111/cts.13011)
Supplement: Supplementary file 4 — Figure S1 [file CTS-14-1133-s004.pdf]

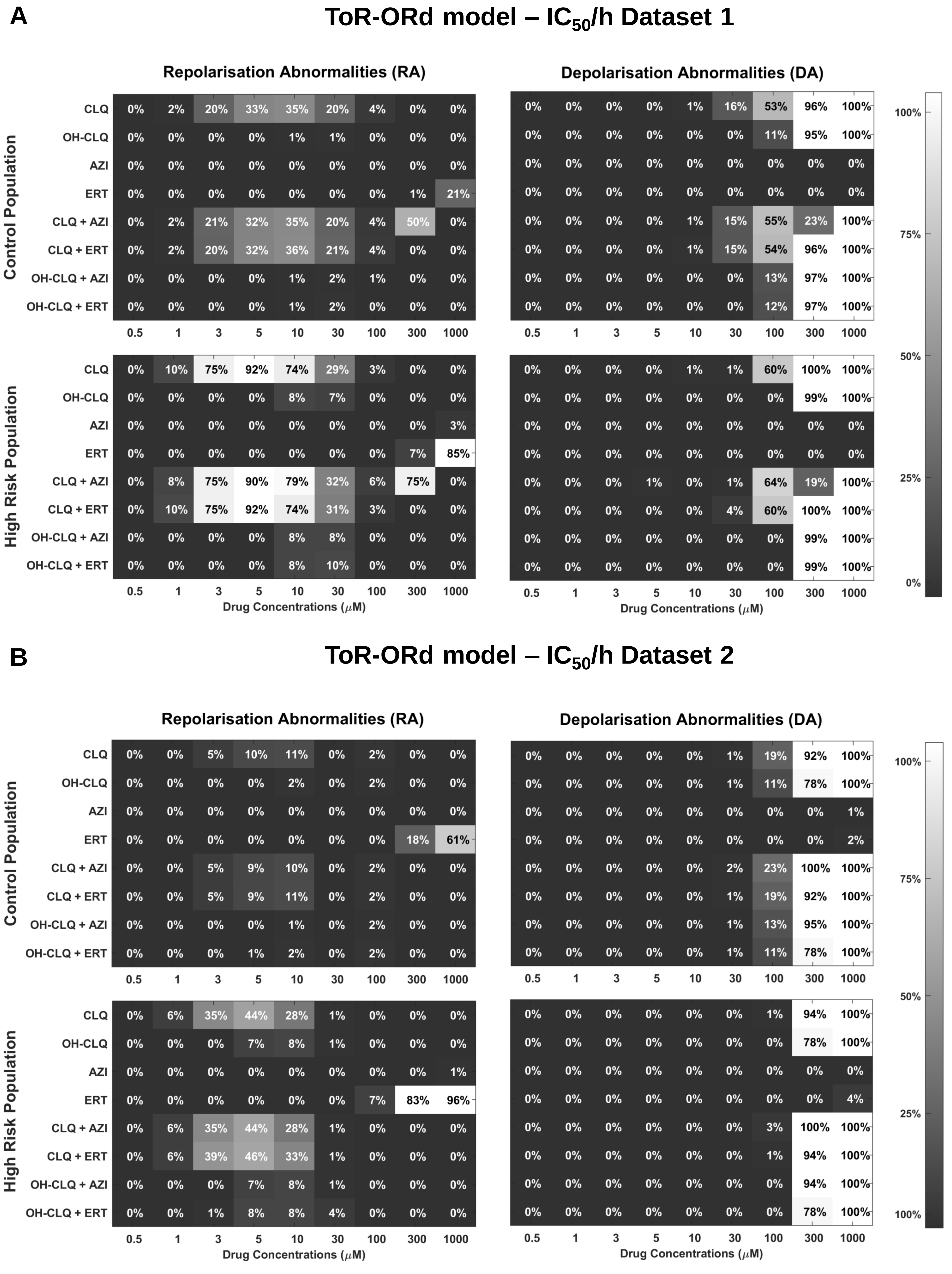
A
ToR-ORd model – IC50/h Dataset 1
Repolarisation Abnormalities (RA)
Depolarisation Abnormalities (DA)
Control Population
CLQ 0% 2% 20% 33% 35% 20% 4% 0% 0%
OH-CLQ 0% 0% 0% 0% 1% 1% 0% 0% 0%
AZI 0% 0% 0% 0% 0% 0% 0% 0% 0%
ERT 0% 0% 0% 0% 0% 0% 0% 1% 21%
CLQ + AZI 0% 2% 21% 32% 35% 20% 4% 50% 0%
CLQ + ERT 0% 2% 20% 32% 36% 21% 4% 0% 0%
OH-CLQ + AZI 0% 0% 0% 0% 1% 2% 1% 0% 0%
OH-CLQ + ERT 0% 0% 0% 0% 1% 2% 0% 0% 0%
0% 0% 0% 0% 1% 16% 53% 96% 100%
0% 0% 0% 0% 0% 0% 11% 95% 100%
0% 0% 0% 0% 0% 0% 0% 0% 0%
0% 0% 0% 0% 0% 0% 0% 0% 0%
0% 0% 0% 0% 1% 15% 55% 23% 100%
0% 0% 0% 0% 1% 15% 54% 96% 100%
0% 0% 0% 0% 0% 0% 13% 97% 100%
0% 0% 0% 0% 0% 0% 12% 97% 100%
0.5 1 3 5 10 30 100 300 1000
High Risk Population
CLQ 0% 10% 75% 92% 74% 29% 3% 0% 0%
OH-CLQ 0% 0% 0% 0% 8% 7% 0% 0% 0%
AZI 0% 0% 0% 0% 0% 0% 0% 0% 3%
ERT 0% 0% 0% 0% 0% 0% 0% 7% 85%
CLQ + AZI 0% 8% 75% 90% 79% 32% 6% 75% 0%
CLQ + ERT 0% 10% 75% 92% 74% 31% 3% 0% 0%
OH-CLQ + AZI 0% 0% 0% 0% 8% 8% 0% 0% 0%
OH-CLQ + ERT 0% 0% 0% 0% 8% 10% 0% 0% 0%
0% 0% 0% 0% 1% 1% 60% 100% 100%
0% 0% 0% 0% 0% 0% 0% 99% 100%
0% 0% 0% 0% 0% 0% 0% 0% 0%
0% 0% 0% 0% 0% 0% 0% 0% 0%
0% 0% 0% 1% 0% 1% 64% 19% 100%
0% 0% 0% 0% 0% 4% 60% 100% 100%
0% 0% 0% 0% 0% 0% 0% 99% 100%
0% 0% 0% 0% 0% 0% 0% 99% 100%
0.5 1 3 5 10 30 100 300 1000
Drug Concentrations (μM)
100% 75% 50% 25% 0%
B
ToR-ORd model – IC50/h Dataset 2
Repolarisation Abnormalities (RA)
Depolarisation Abnormalities (DA)
Control Population
CLQ 0% 0% 5% 10% 11% 0% 2% 0% 0%
OH-CLQ 0% 0% 0% 0% 2% 0% 2% 0% 0%
AZI 0% 0% 0% 0% 0% 0% 0% 0% 0%
ERT 0% 0% 0% 0% 0% 0% 0% 18% 61%
CLQ + AZI 0% 0% 5% 9% 10% 0% 2% 0% 0%
CLQ + ERT 0% 0% 5% 9% 11% 0% 2% 0% 0%
OH-CLQ + AZI 0% 0% 0% 0% 1% 0% 2% 0% 0%
OH-CLQ + ERT 0% 0% 0% 1% 2% 0% 2% 0% 0%
0% 0% 0% 0% 0% 1% 19% 92% 100%
0% 0% 0% 0% 0% 1% 11% 78% 100%
0% 0% 0% 0% 0% 0% 0% 0% 1%
0% 0% 0% 0% 0% 0% 0% 0% 2%
0% 0% 0% 0% 0% 2% 23% 100% 100%
0% 0% 0% 0% 0% 1% 19% 92% 100%
0% 0% 0% 0% 0% 1% 13% 95% 100%
0% 0% 0% 0% 0% 1% 11% 78% 100%
0.5 1 3 5 10 30 100 300 1000
High Risk Population
CLQ 0% 6% 35% 44% 28% 1% 0% 0% 0%
OH-CLQ 0% 0% 0% 7% 8% 1% 0% 0% 0%
AZI 0% 0% 0% 0% 0% 0% 0% 0% 1%
ERT 0% 0% 0% 0% 0% 0% 7% 83% 96%
CLQ + AZI 0% 6% 35% 44% 28% 1% 0% 0% 0%
CLQ + ERT 0% 6% 39% 46% 33% 1% 0% 0% 0%
OH-CLQ + AZI 0% 0% 0% 7% 8% 1% 0% 0% 0%
OH-CLQ + ERT 0% 0% 1% 8% 8% 4% 0% 0% 0%
0% 0% 0% 0% 0% 0% 1% 94% 100%
0% 0% 0% 0% 0% 0% 0% 78% 100%
0% 0% 0% 0% 0% 0% 0% 0% 0%
0% 0% 0% 0% 0% 0% 0% 0% 4%
0% 0% 0% 0% 0% 0% 3% 100% 100%
0% 0% 0% 0% 0% 0% 1% 94% 100%
0% 0% 0% 0% 0% 0% 0% 94% 100%
0% 0% 0% 0% 0% 0% 0% 78% 100%
0.5 1 3 5 10 30 100 300 1000
Drug Concentrations (μM)
100% 75% 50% 25% 100%

C

ORd-CiPA model – IC_{50}/h Dataset 1

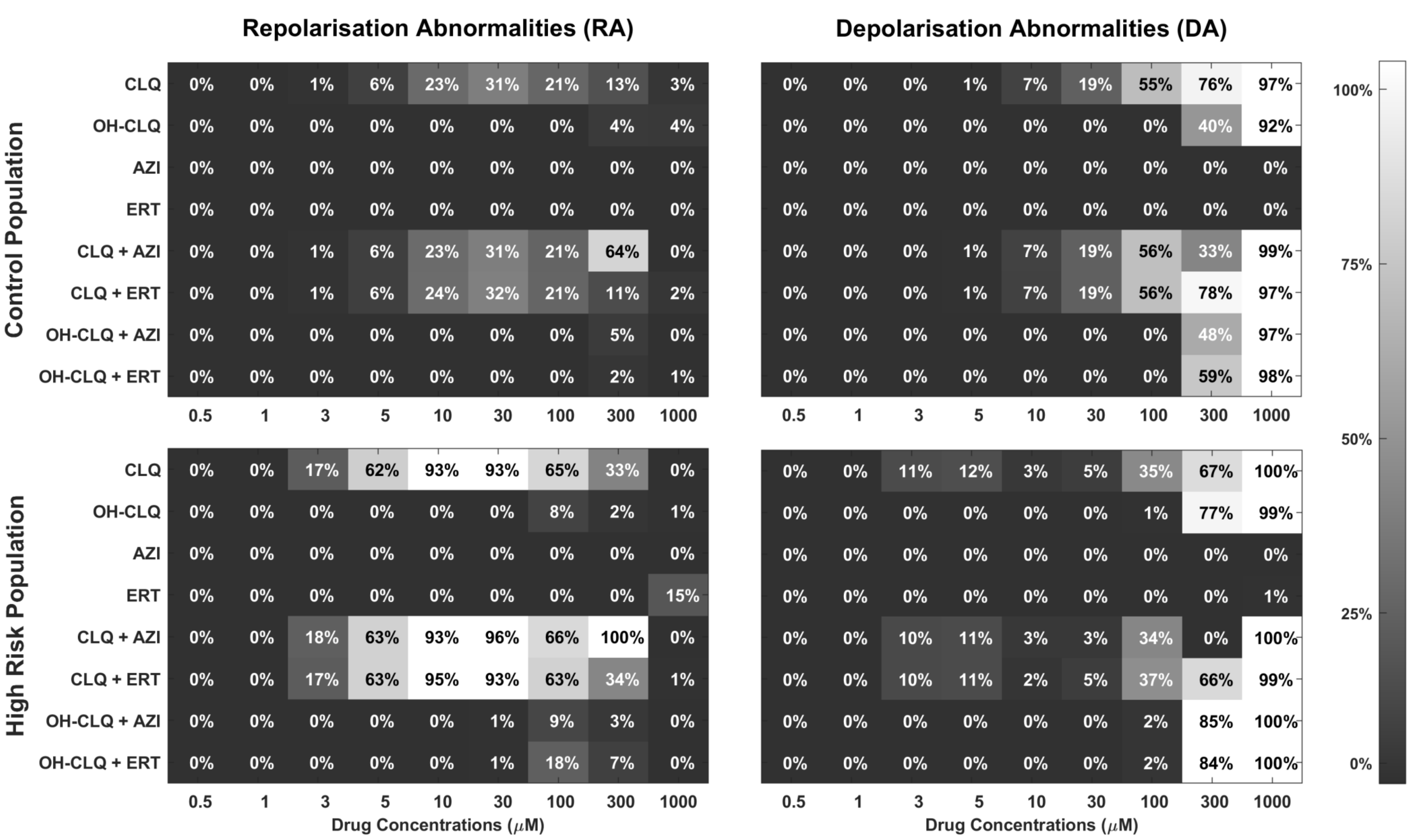

D

ORd-CiPA model – IC_{50}/h Dataset 2

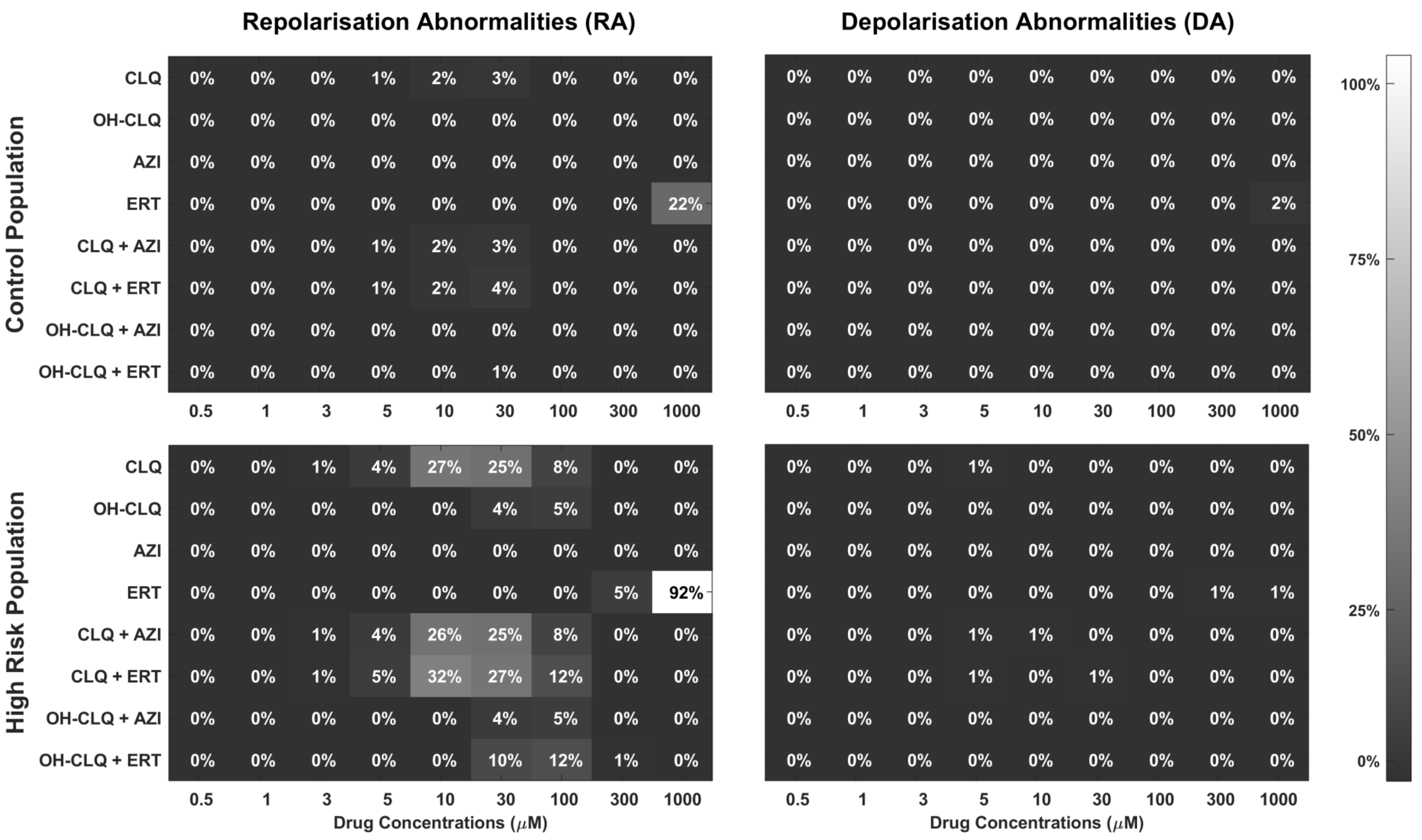

E **ORd model – IC_{50}/h Dataset 1**

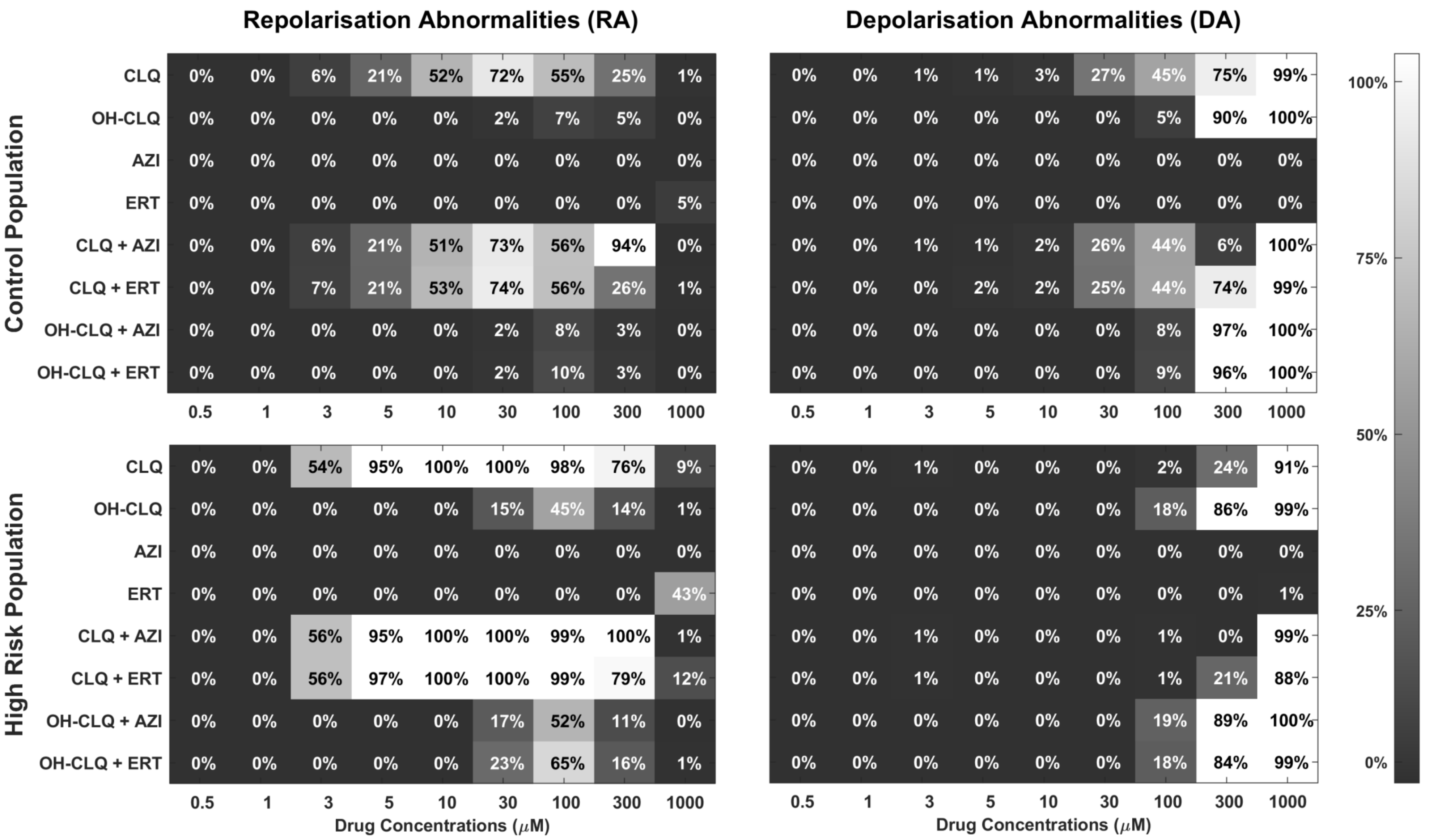

F **ORd model – IC_{50}/h Dataset 2**

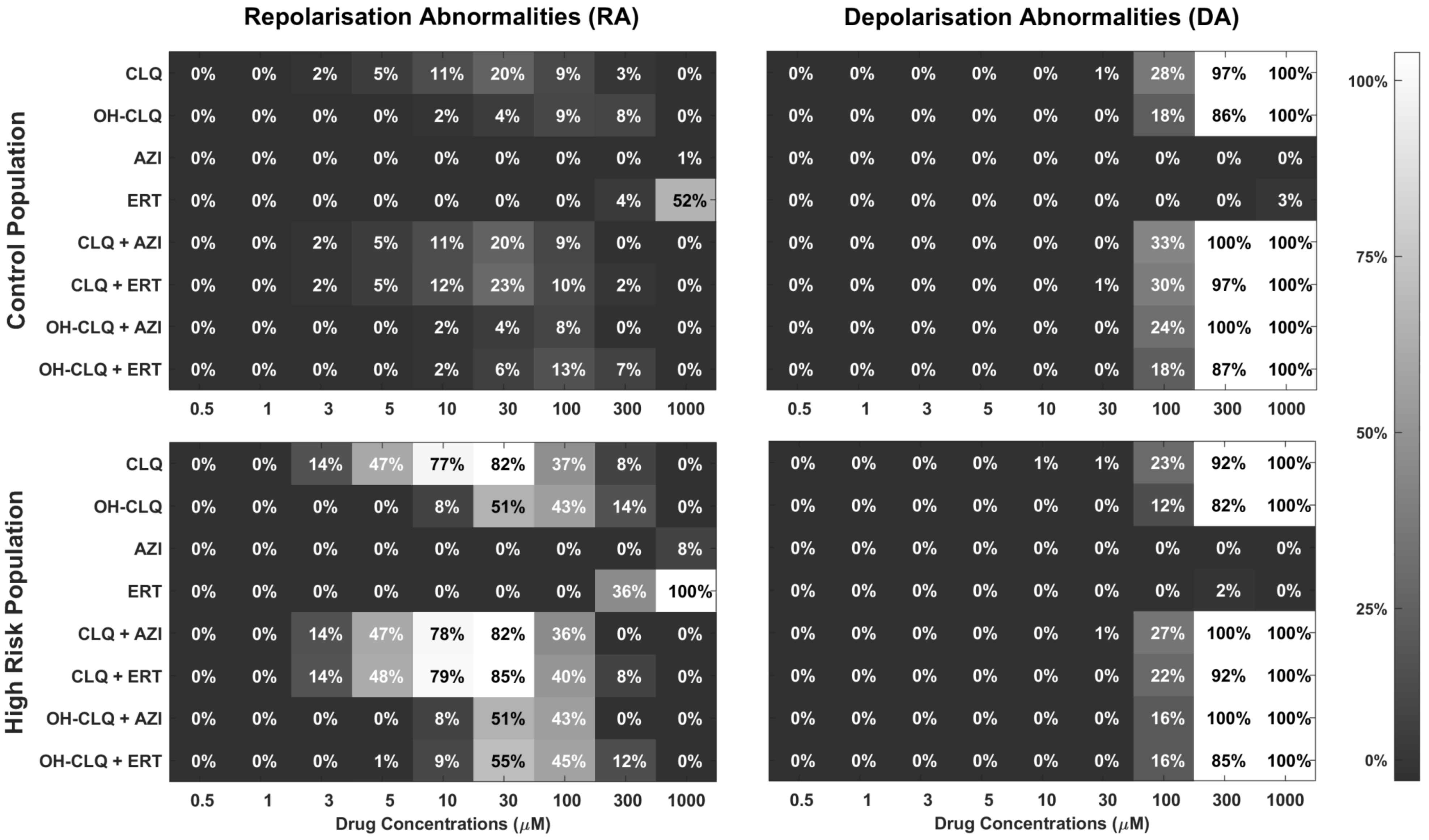

Figure S1: Occurrence of RA and DA for all 6 populations and both IC_{50}/Hill coefficient Datasets. As expected, RA are more prevalent in the high-risk compared to control populations. RA are also more prevalent when considering simulation with the Dataset #1 compared to Dataset #2 (Table 1). Results are similar across different models, even though RA tends to appear at lower concentrations when considering the ToR-ORd model.
